# Parkinsonism in Genetic Neurodevelopmental Disorders: A Systematic Review

**DOI:** 10.1002/mdc3.13577

**Published:** 2022-10-31

**Authors:** Emma N.M.M. von Scheibler, Agnies M. van Eeghen, Tom J. de Koning, Mark L. Kuijf, Janneke R. Zinkstok, Annelieke R. Müller, Thérèse A.M.J. van Amelsvoort, Erik Boot

**Affiliations:** ^1^ Advisium's Heeren Loo Zorggroep Amersfoort The Netherlands; ^2^ Department of Psychiatry and Neuropsychology Maastricht University Maastricht The Netherlands; ^3^ Emma Children's Hospital University of Amsterdam Amsterdam The Netherlands; ^4^ Department of Genetics University of Groningen Groningen The Netherlands; ^5^ Expertise Centre Movement Disorders Groningen University Medical Centre Groningen Groningen The Netherlands; ^6^ Pediatrics, Department of Clinical Sciences Lund University Lund Sweden; ^7^ Department of Neurology Maastricht University Medical Centre Maastricht The Netherlands; ^8^ Department of Psychiatry Radoud University Medical Centre Nijmegen The Netherlands; ^9^ Karakter child and adolescent psychiatry Nijmegen The Netherlands; ^10^ Department of Psychiatry and Brain Center University Medical Center Utrecht Utrecht The Netherlands; ^11^ The Dalglish Family 22q Clinic University Health Network Toronto Ontario Canada

**Keywords:** genetic, neurodevelopmental disorder, intellectual disability, parkinsonism, Parkinson's disease

## Abstract

**Background:**

With advances in clinical genetic testing, associations between genetic neurodevelopmental disorders and parkinsonism are increasingly recognized. In this review, we aimed to provide a comprehensive overview of reports on parkinsonism in genetic neurodevelopmental disorders and summarize findings related to genetic diagnosis, clinical features and proposed disease mechanisms.

**Methods:**

A systematic literature review was conducted in PubMed and Embase on June 15, 2021. Search terms for parkinsonism and genetic neurodevelopmental disorders, using generic terms and the Human Phenotype Ontology, were combined. Study characteristics and descriptive data were extracted from the articles using a modified version of the Cochrane Consumers and Communication Review Group's data extraction template. The protocol was registered in PROSPERO (CRD42020191035).

**Results:**

The literature search yielded 208 reports for data‐extraction, describing 69 genetic disorders in 422 patients. The five most reported from most to least frequent were: 22q11.2 deletion syndrome, beta‐propeller protein‐associated neurodegeneration, Down syndrome, cerebrotendinous xanthomatosis, and Rett syndrome. Notable findings were an almost equal male to female ratio, an early median age of motor onset (26 years old) and rigidity being more common than rest tremor. Results of dopaminergic imaging and response to antiparkinsonian medication often supported the neurodegenerative nature of parkinsonism. Moreover, neuropathology results showed neuronal loss in the majority of cases. Proposed disease mechanisms included aberrant mitochondrial function and disruptions in neurotransmitter metabolism, endosomal trafficking, and the autophagic‐lysosomal and ubiquitin‐proteasome system.

**Conclusion:**

Parkinsonism has been reported in many GNDs. Findings from this study may provide clues for further research and improve management of patients with GNDs and/or parkinsonism.

Although genetic brain disorders are traditionally dichotomized into neurodevelopmental and neurodegenerative disorders, it is becoming clear that some conditions are associated with both neurodevelopmental problems and neurodegeneration,^1^ and there are indications for a shared underlying genetic susceptibility.^2^, ^3^ Indeed, with advances in clinical genetic testing for neurological disease and an increase in life expectancy because of improved medical care, a growing number of genetic neurodevelopmental disorders (GNDs) has been associated with the development of Parkinson's disease and other forms of parkinsonism.^4^, ^5^, ^6^

Although individually rare, research in GNDs may help to better understand different pathophysiological mechanisms that are believed to play a role in the development of parkinsonism. An analogy may be found in Down syndrome that is associated with early‐onset Alzheimer's dementia. Research in Down syndrome has provided important insights into dementia etiology^7^ and has facilitated research into disease‐modifying treatments.^8^, ^9^ Genetic variants associated with GNDs are often identified in childhood, before neurologic symptoms emerge, and long‐term follow up of children and adolescents with GNDs may increase knowledge on disease trajectories. In addition to the potential to identify disease and/or mechanism‐specific treatment through animal models that are available for many genetic conditions, recognition of GNDs associated with parkinsonism may improve anticipatory care for patients with these GNDs. Therefore, knowledge about GNDs that may present with parkinsonism is important to optimize clinical practice and further research.

In this systematic literature review, a comprehensive overview is provided of studies that reported on parkinsonism in GNDs. We summarize findings related to patient characteristics, parkinsonian features, and proposed disease mechanisms and outline implications for clinical practice and future research.

## Methods

The study protocol was published in the PROSPERO International Register for Systematic Reviews (CRD42020191035). We made a few minor amendments to the initial protocol, including the addition of two co‐authors, a repeated search, and inclusion of non‐rare GNDs (eg, Down syndrome). We followed the Preferred Reporting Items for Systematic Reviews and Meta‐Analysis Protocol (PRISMA).^10^

### Search Strategy and Selection

We performed a comprehensive literature search in PubMed and Embase on June 15, 2021 (see the Supplementary Methods and Fig. S1). We used the Human Phenotype Ontology (HPO) (https://hpo.jax.org/) term “neurodevelopmental abnormality” (HP:0012759) for GNDs, in combination with database‐specific subheadings and text words for “Parkinson's disease/parkinsonism.”^11^ Two reviewers independently screened titles and abstracts for eligibility. Relevant records were screened against selection criteria for inclusion based on full‐text. In case of uncertainty, two other reviewers were consulted. Discrepancies were discussed until consensus was reached.

We included all reports on patients who met the inclusion criteria for a neurodevelopmental disorder (ie, listed in the HPO as “neurodevelopmental” and/or representing patients showing a deviation from normal of the neurological development in childhood, including any or all aspects of the development of personal, social, motor, and cognitive abilities, in the presence of a disease‐causing genetic variant) and parkinsonism (bradykinesia in combination with either rigidity, rest tremor or both),^12^ or who were likely to have Parkinson's disease, operationalized as a clear beneficial response to levodopa, reduced dopamine transporter binding with dopaminergic imaging, and/or neuropathological hallmarks of Parkinson's disease. We considered hypokinesia, akinesia, and hypomimia equal to bradykinesia if parkinsonism was diagnosed, in case bradykinesia was not explicitly noted. We excluded: (1) reports on patients with uncertain genetic etiology (ie, not molecularly confirmed or according to standard clinical diagnostic criteria); (2) ultra‐rare genetic conditions with less than three reported cases, because of difficulties determining whether these were associated with brain development and because significance of the variant was often uncertain; and (3) neurogenetic disorders with solely motor aspects of development in childhood, such as most spinocerebellar ataxias. Results were limited to studies written in English and reports that provided original data. We performed a thorough cross‐reference check.

### Quality Assessment

We critically appraised full‐text articles using the National Heart, Lung and Blood Institute (NIH‐NHLBI) study quality assessment tools for case–control and cohort studies.^13^

### Data Extraction

We extracted study characteristics and descriptive data from the included studies using a modified version of the Cochrane Consumers and Communication Review Group's data extraction template (https://cccrg.cochrane.org). Data extracted comprised: general information (eg, first author, year of publication, study design, and number of patients reported to have parkinsonism), patient characteristics (eg, age, sex, presence/absence and severity of intellectual disability, full scale intelligence quotient), other cognitive disorders or problems on other cognitive domains, genetics (eg, specific diagnosis and results of genetic testing including those related to Parkinson's disease disease‐causing and risk genes), and parkinsonian features (eg, age at motor onset, presence/absence of cardinal motor features and [proposed] pathophysiology).

### Data Presentation

**FIG. 1:**
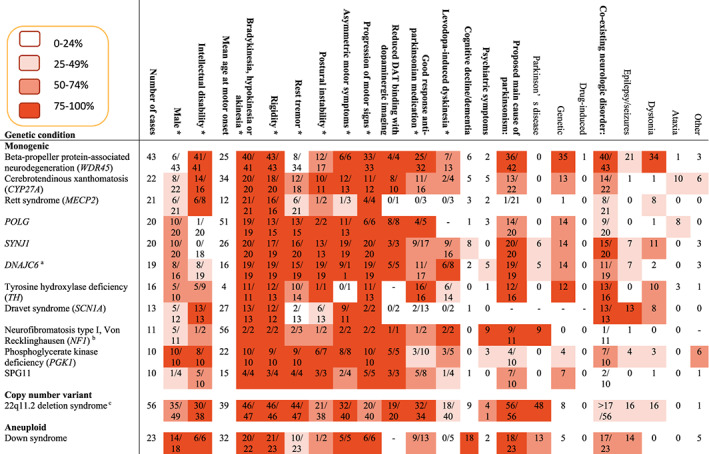
Patient characteristics and parkinsonian features in genetic neurodevelopmental disorders that were reported at least 10 times. The complete heat map, including conditions that were reported less than 10 times, is provided in Fig. S2. *The numerator represents how many patients were reported to present with a specific feature, and the denominator represents the number of patients with data available. Additional genetic variants with potential relevance to parkinsonism were reported: ^a^ *LRRK2*, ^b^ *PRKN*, ^c^ *PRKN*. ‐ = unknown.

We use descriptive summaries to present our findings. Patient characteristics and parkinsonian features per genetic disorder are presented in a heatmap, illustrating which features were most reported per genetic disorder in addition to the proposed underlying mechanisms (Fig. 1 and Fig. S2).

## Results

Of a total of 5269 identified records, 208 reports met the inclusion criteria: 186 case studies and 22 observational case–control or cohort studies (see flow‐chart, Fig. S1). The 208 reports contained individual data of 422 patients with 69 different GNDs. Sex was reported in 395 patients, and 212 (53.7%) were male. The median age at last examination in 362/422 of the patients was 35 (range, 0–77) years (for a list of included studies see Tables S1 and S2, and for excluded studies Table S3).

### Quality Assessments

The assessment of study quality for cohort and case–control studies is detailed in Tables S4 and S5. Of the 22 cohorts and case–control studies, 15 were rated good, six were rated fair and one case–control study was considered to be of poor quality because of a very limited description of aims and methods.

### Different GNDs that Presented with Parkinsonism

Most patients in the cohort had a monogenic disorder, with more than 10 patients, from most to least frequent, reported for each of the following: beta‐propeller protein‐associated neurodegeneration (BPAN), cerebrotendinous xanthomatosis, Rett syndrome, *POLG*, *SYNJ1*, *DNAJC6*, tyrosine hydroxylase deficiency, Dravet syndrome *(SCN1A)*, neurofibromatosis type I, phosphoglycerate kinase deficiency, and spastic paraplegia type 11. Between five and 10 patients had juvenile neuronal ceroid lipofuscinosis (JNCL), *PTRHD1*, *RAB39B*, X‐linked parkinsonism with spasticity, Alexander disease, dopa‐responsive dystonia‐parkinsonism (*NR4A2*), Fragile‐X syndrome or spastic paraplegia type 15 (SPG15). The only copy number variation (CNV) and aneuploid conditions with more than 10 reported patients were 22q11.2 deletion syndrome and Down syndrome, respectively. The only aneuploid condition with between five and ten patients reported was Klinefelter syndrome. Forty‐nine patients without genetic confirmation, but with a biochemical or clinical diagnosis were included, with five or more patients reported for: Rett syndrome, neurofibromatosis type I, Down syndrome, and glutaric aciduria type one. Less than five cases were reported for another 47 GNDs (see Fig. S2).

Thirteen of 422 patients (3.1%) were reported to have an additional genetic variant of potential clinical relevance for the development of parkinsonism, annotated in Figures 1 and S2.

### Parkinsonian Features

The median age at onset of motor symptoms, available for 349 of 422 patients, was 26 (range, 0–66) years. For those with motor symptoms reported, rigidity was the most prevalent parkinsonian feature after bradykinesia, being present in 330 of 346 patients (95.4%). Rest tremor was seen in 218 of 355 (61.4%), postural instability in 138 of 179 (77.1%), asymmetry in 176 of 212 (83.0%), and progression of motor symptoms in 239 of 267 (89.5%) patients with data. Reduced dopamine transporter (DAT)‐binding was seen in the majority of the 39 GNDs with available dopaminergic imaging results, but not in Rett syndrome, Dravet syndrome (SCN1A), dihydropteridine reductase deficiency, and *CLTC*. A clear and beneficial response to antiparkinsonian medication was reported in 187 of 262 patients (71.4%). GNDs with questionable or no response to antiparkinsonian medication in most cases were Rett syndrome, Dravet syndrome (*SCN1A)*, phosphoglycerate kinase deficiency, dystonia 16, Leigh syndrome, and Menkes disease. Levodopa‐induced dyskinesia was described in 74 of 157 patients (47.1%).

### Neurological and Psychiatric Comorbidity

Intellectual functioning was reported for 320 of 422 patients (75.8%), with intellectual disability present in 193 patients (60.3%). Cognitive decline or dementia was reported in 70 patients (16.6%). In 103 patients (24.4%) psychiatric comorbidity was present, such as depression, anxiety or psychosis. An additional neurologic disorder, not including dementia, was reported in 256 (60.7%) of 422 patients: dystonia in 128 (30.3%), epilepsy/seizures in 112 (26.5%) and ataxia in 38 patients (9.0%).

### Pathophysiology

**FIG. 2:**
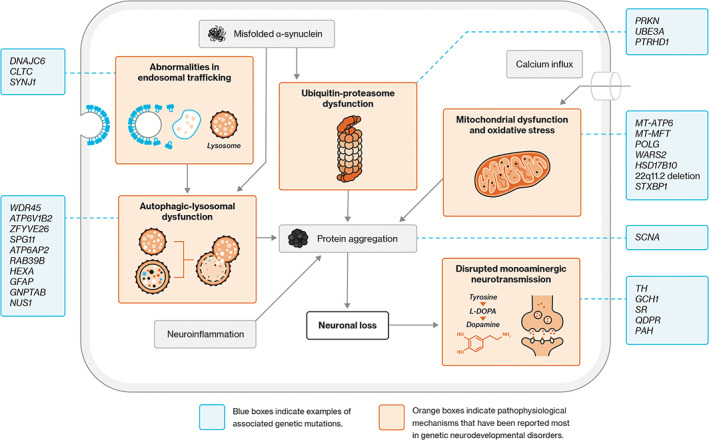
Schematic diagram depicting mechanisms that may underlie parkinsonism in genetic neurodevelopmental disorders, with examples of associated genetic variants.

The main causes of parkinsonism, as proposed in the articles, were the underlying genetic disorder, Parkinson's disease and drug‐induced parkinsonism, with the latter reported in only two patients. The proposed pathophysiological mechanism (summarized in Table 1 per genetic disorder and depicted in Fig. 2), from most to least frequent, included: abnormalities in mitochondrial function and oxidative stress, lysosomal‐autophagic function, endosomal trafficking, and ubiquitin‐proteasome system. Disruptions of monoaminergic neurotransmitter metabolism were also implicated in several GNDs that presented with parkinsonism.

**TABLE 1:** Pathophysiologic mechanisms that may underlie parkinsonism in genetic neurodevelopmental disorders

Genetic condition	Mechanisms/pathophysiology that may be considered
	**Mitochondrial dysfunction**
HSD10 (*HSD17B10*)	*HSD17B10* encodes an enzyme that is essential for mitochondrial maintenance.^48^ Pathogenic variants may affect enzyme function and result in mitochondrial dysfunction.
Leigh syndrome (*MT‐ATP6* and *MT‐MFT*)	Leigh syndrome is caused by over 50 different mitochondrial and nuclear encoded genes, most often affecting the respiratory chain and oxidative phosphorylation.^49^ Mitochondrial dysfunction may result in brain stem and basal ganglia lesions.
Leigh‐like syndrome (*MT‐TI*)	*MT‐TI* is a mitochondrial gene of which pathogenic variants may result in mitochondrial dysfunction and basal ganglia lesions, similar to what has been proposed for Leigh syndrome.
*MT‐CYB*	*MT‐CYB* is a mitochondrial gene that encodes for a component of the respiratory chain. Pathogenic variants may result in mitochondrial dysfunction and progressive basal ganglia lesions, as has been proposed for Leigh syndrome.
*POLG*	*POLG* encodes a DNA polymerase that is essential for replication of mitochondrial DNA. Mice that were homozygous for variants that may disrupt the function of POLG protein exhibited premature aging.^50^, ^51^
*WARS2*	Pathogenic variants in *WARS2*, which encodes for the WARS2 protein located in the mitochondrion, may result in respiratory chain defects and nigrostriatal degeneration.^52^
	**Mitochondrial dysfunction combined with other mechanisms**
22q11.2 deletion syndrome	The 22q11.2 deletion region encompasses several genes including *COMT*, essential for catecholamine degradation, and six mitochondrial genes.^53^, ^54^, ^55^ Haploinsufficiency of these genes may result in dopamine autotoxicity and mitochondrial dysfunction.^56^
Down syndrome	Mitochondrial dysfunction, neuroinflammation, oxidative stress, and lysosomal dysfunction have all been reported in Down syndrome.^57^, ^58^, ^59^
Early infantile epileptic encephalopathy 4 (*STXBP1*)	Pathogenic variants in *STXBP1* may cause significant impairment of complex I of the mitochondrial respiratory chain and may disrupt the self‐replicating aggregation of α‐synuclein.^60^
Glutaric aciduria type I (*GCDH*)	*GCDH* plays a key role in the catabolism of lysine, hydroxylysine, and tryptophan. Deficiency of *GCDH* leads to accumulation of glutaric acid and 3‐hydroxyglutaric acid that can induce neuronal death through excitotoxicity as well as mitochondrial dysfunction and altered neurotransmission.^61^
Mevalonic aciduria (*MVK*)	Pathogenic variants in *MVK* may result in mitochondrial dysfunction, impaired cholesterol biosynthesis, toxic basal ganglia mevalonate accumulation, and intracerebral inflammation.^62^, ^63^
*NR4A2*	Pathogenic variants in *NR4A2* are implicated in development and survival of dopaminergic neurons in the substantia nigra, and may lower expression of genes associated with mitochondrial function and oxidative phosphorylation.^44^
Pyruvate carboxylase deficiency (*PC*)	*PC* encodes for pyruvate carboxylase, a mitochondrial enzyme that catalyzes pyruvate to oxaloacetate, intermediates in the Krebbs cycle and is important for neurotransmitter synthesis.^64^
SPG10 (*KIF5A*)	Axonal transport defect of mitochondria has been shown in a *KIF5A* knockout mouse model.^65^
	**Autophagic‐lysosomal system**
Alexander disease (*GFAP*)	*GFAP* encodes for glial fibrillary acidic protein (GFAP), an intermediate filament protein in astrocytes. GFAP accumulation has been associated with autophagy in astrocytic cells.^66^
BPAN (*WDR45*)	Pathogenic variants of *WDR45*, encoding for WIPI4 protein, may cause iron accumulation in the basal ganglia by impeding autophagy,^67^ that may result in neuroinflammation and swelling of the substantia nigra.
Christianson syndrome (*SLC9A6*)	*SLC9A6* encodes the endosomal Na+/H+ exchanger 6 and is involved in endosomal luminal pH and trafficking, synapse development, and plasticity.^68^ Findings in Slc9a6 knock‐out mice were consistent with endosomal‐lysosomal dysfunction.^69^
DOORS syndrome (*ATP6V1B2*)	*ATP6V1B2* encodes a subunit of the lysosomal transmembrane proton pump. Altered lysosomal pH is associated with chronic changes in autophagy.^70^
JNCL (*CLN3*)	*CLN3* is involved in autophagic‐lysosomal function. *CLN3* is required for fusion of autophagosomes to lysosomes.^71^
Mucolipidosis type II (*GNPTAB*)	Pathogenic variants in *GNPTAB*, that encodes for GlcNAc‐1‐phosphotransferase, may cause lysosomal accumulation of nondegraded material, leading to neuronal dysfunction.^72^
*NUS1*	Deficiency of *NUS1*, encoding the Nogo B receptor, may result in lysosomal defects, most likely caused by lysosomal cholesterol accumulation.^73^
*RAB39B*	Ras‐related proteins play an essential role in neuronal maintenance, survival and synapse formation. It has been suggested that *RAB39B* plays a role in autophagy of dopaminergic neurons. Loss of function may impair the clearance of α‐synuclein.^74^, ^75^
SPG15 (*ZFYVE26*)	Pathogenic variants of *ZFYVE26* encoding spastizin, a protein mediating autophagic lysosome reformation, are believed to cause abnormal lysosomal storage.^76^
SPG11 (*SPG11*)	Pathogenic variants of *SPG11* encoding spatacsin, a protein mediating autophagic lysosome reformation may cause abnormal lysosomal storage.^77^
Tay‐Sachs disease (*HEXA*)	*HEXA* encodes for β‐hexosaminidase A, a lysosomal enzyme that degrades GM2 ganglioside. Deficiency of this enzyme A has been associated with GM2 ganglioside accumulation nerve cells.^78^
X‐linked parkinsonism with spasticity (*ATP6AP2*)	*ATP6AP2* encodes an accessory unit of an essential lysosomal enzyme. Haploinsufficiency of *ATP6AP2* may lead to autophagy defects, disrupted presynaptic transmission, and neurodegeneration.^79^
	**Disorders of neurotransmitter metabolism**
6p25 deletion, involving *FOXC1*	Pathogenic variants in *FOXC1* may affect genes involved in dopamine synthesis and dopaminergic neuronal development.^80^, ^81^
Dihydropteridine reductase deficiency (*QDPR*)	Deficiency of dihydropteridine reductase, that is required for resynthesis of tetrahydrobiopterin, an essential cofactor for the activity of phenylalanine‐, tryptophan, and tyrosine hydroxylases, may impair neurotransmitter synthesis.^82^
*DNAJC12*	*DNAJC12* has a critical role in chaperoning amino‐acid hydrolase interactions required for catecholamine synthesis.^83^
Dopamine transporter deficiency syndrome (*SLC6A3*)	*SLC6A3* encodes for the dopamine transporter (DAT). DAT deficiency syndrome may lead to impaired DAT activity, apoptotic neurodegeneration, and dopamine toxicity.^84^
Dravet syndrome (*SCN1A*)	*SCN1A*, that encodes a voltage‐gated sodium channel, may lead to impaired neurotransmitter release.^85^
GTP cyclohydrolase 1 deficiency, dopa‐responsive dystonia (*GCH1*)	GTP cyclohydrolase 1 is important for the biosynthesis of tetrahydrobiopterin, an essential cofactor for the activity of phenylalanine, tryptophan, and tyrosine hydroxylases. Deficiency of this enzyme may disrupt neurotransmitter synthesis.^86^
Neurofibromatosis type I (*NF1)*	*NF1*, a tumor suppressor gene, encodes for neurofibromin. Among other genes, it is involved in the activation of GTPase, mTOR signaling, learning (via impaired long‐term potentiation), and regulation of dopamine homeostasis.^87^
Phenylketonuria (*PAH*)	*PAH* encodes for phenylalanine hydroxylase. Deficiency results in a decreased conversion of phenylalanine to tyrosine. Phenylalanine inhibits dopamine and serotonin synthesis in the brain by inhibition of tyrosine and tryptophan transport, and inhibition of tyrosine and tryptophan hydroxylases.^88^, ^89^
*PPP2R5D*	*PPP2R5D* encodes a regulatory subunit of protein phosphatase‐2A (PP2A), an intracellular serine/threonine phosphatase. PP2A regulates phosphorylation of one site (S129) of α‐synuclein. Increased activity of PP2A influences tyrosine hydroxylase and subsequently may affect dopamine synthesis.^90^, ^91^
Sepiapterin reductase deficiency (*SR*)	Deficiency of sepiapterin reductase, essential for tetrahydrobiopterin biosynthesis, may result in disturbed dopaminergic and serotonergic neurotransmission.^92^
Tyrosine hydroxylase deficiency, dopa‐responsive dystonia (*TH*)	Deficiency of tyrosine hydroxylase, the rate‐limiting step in dopamine biosynthesis, may lead to a shortage of dopamine.^93^
	**Endosomal trafficking**
*CLTC*	A defective clathrin heavy chain polypeptide protein, caused by pathogenic variants of *CLTC*, may result in depletion of biogenic amines by altering their synaptic turnover.^94^
*DNAJC6*	*DNAJC6* encodes for auxilin, a neuronally expressed J‐chaperone protein involved in the uncoating of clathrin‐coated vesicles, which is necessary for the regeneration of synaptic vesicles. Impaired uncoating is thought to lead to neurotransmission deficits.^95^
*SYNJ1*	*SYNJ1* encodes a phosphoinositide phosphatase called synaptojanin1 and plays an important role in early endosomal compartments and clathrin‐mediated endocytosis.^96^
	**Ubiquitin‐proteasome system**
Angelman syndrome, involving *UBE3A*	*UBE3A* encodes the ubiquitin‐protein ligase E3A, part of the ubiquitin‐proteolytic pathway,^97^ that has been suggested to be involved in the clearance of alpha‐synuclein.^98^
Partial 6q trisomy, involving *PRKN*	Pathogenic variants of *PRKN* are associated with early‐onset autosomal recessive Parkinson's disease.^99^ *PRKN* encodes the E3 ubiquitin‐protein ligase Parkin, involved in mitophagy, and possibly in the formation of Lewy bodies.^100^
*PTRHD1*	*PTRHD1* encodes for peptidyl‐tRNA hydrolase that belongs to the PTH2 family. The deduced ubiquitin‐like domain‐binding protein is thought to suppress ubiquitin‐mediated protein degradation.^101^ α‐Synuclein homeostasis is maintained by proper function of the ubiquitin‐proteasome system.
	**Other**
Cerebrotendinous xanthomatosis (*CYP27A*)	Accumulation of cholesterol and cholestanol cause neurotoxicity and axonopathy.^102^
Incontinentia pigmenti (*IKBKG*)	It has been suggested that pathogenic variants in *IKBKG*, involved in neuronal anti‐apoptotic signaling, may cause neurodegeneration.^103^
Klinefelter syndrome	Melatonin may have a neuroprotective role in Parkinson's disease. It has been suggested that reduced melatonin levels in Klinefelter syndrome may play a role in the development of parkinsonism.^104^, ^105^
L2‐hydroxyglutaric aciduria (*L2HGDH*)	*L2HGDH* encodes for l‐2‐hydroxyglutarate (L2HG) dehydrogenase that oxidizes L‐2‐hydroxyglutarate to α‐ketoglutarate. L2HG deficiency may result in impaired hippocampal neurogenesis and neurodegeneration in adult mouse brains.^106^
Menkes disease (*ATP7A*)	Haploinsufficiency of *ATP7A*, encoding a transmembrane copper‐transporting ATPase, may result in dysregulation of copper metabolism in the basal ganglia.^107^, ^108^
Molybdenum cofactor deficiency type B (*MOCS2*)	*MOCS2* encodes for molybdenum cofactor. Deficiency leads to loss of sulfite oxidase activity, resulting in cumulative metabolic effects on the basal ganglia.^109^ Elevated concentrations of S‐sulfocysteine and toxic sulfite may trigger neuronal apoptosis.^110^
Partial 4q trisomy, involving *SNCA*	*SNCA* encodes α‐synuclein, the primary component of Lewy bodies. The patient with partial 4q trisomy had a de novo *SNCA* duplication. Other genes in the duplicated region may have contributed to the phenotype.^111^
Phosphoglycerate kinase deficiency I (*PGK1*)	Phosphoglycerate kinase is an important enzyme in the glycolytic pathway. It has been suggested that neuronal damage occurs as a consequence of energy failure.^112^
SCA27 (*FGF14*)	*FGF14*, expressed in axons of the striatopallidal and striatonigral pathways, encodes a regulatory protein of voltage‐gated sodium channels (Nav1.6). Haploinsufficiency of *FGF14* may alter expression of sodium channels with impaired firing of Purkinje neurons.^113^
Smith‐Magenis syndrome (*RAI1*)	Pathogenic variants of *RAI1* may result in an inversion of circadian melatonin secretion with a lack of nocturnal melatonin, which may play a role in the development of parkinsonism.^104^, ^105^, ^114^

### Neuropathology

Neuropathological findings were reported for 16 patients (11 male; 73.3%) at median age 57 (range, 2–74) years: 22q11.2 deletion syndrome (n = 3), Down syndrome (n = 2), *POLG* (n = 2), BPAN (n = 1), CTX (n = 1), Cornelia de Lange syndrome (n = 1), *DNAJC12* (n = 1), DOORS syndrome (n = *1*), 5, 10‐methylenetetrahydrofolate reductase deficiency (n = 1), *NR4A2* (n = 1), *PPP2R5D* (n = 1), and *RAB39B* (n = 1) (Table 2). In 14 patients, the presence of Lewy bodies was examined, with positive findings in six cases (42.9%): 22q11.2 deletion syndrome (n = 2/3), Down syndrome (*n* = 2/2), *NR4A2* (n = 1/1) and *RAB39B* (n = 1/1). Neurites were examined in nine patients and present in seven: 22q11.2 deletion syndrome (n = 2/3), Cornelia de Lange syndrome (n = 1/1), *DNAJC12* (n = 1/1), Down syndrome (n = 1/1), *NR4A2* (n = 1/1) and *RAB39B* (n = 1/1). Neuronal loss was reported in all, but one patient (92.9%), the latter who had DOORS syndrome.

**TABLE 2:** Neuropathological findings in patients with genetic neurodevelopmental disorders and parkinsonism

Genetic neurodevelopmental disorder	Age, y	Sex	LB	LN	Neuronal loss
22q11.2 deletion syndrome^17^	56	F	+	+	+
	58	M	+	+	+
	61	M	−	−	+
BPAN^115^	27	F	−	NR	+
CTX^102^	56	M	NR	NR	+
Cornelia de Lange syndrome^116^	38	M	−	+ *	+
*DNAJC12* ^47^	74	M	−	+ *	+
DOORS syndrome^70^	72	M	−	NR	−
Down syndrome^117^, ^118^	54	M	+	+ *	+
	49	M	+	NR	+
MTHFR deficiency^119^	2	F	NR	NR	+
*NR4A2* ^120^	74	NR	+	+	+
*POLG* ^45^	61	F	−	−	+
	60	M	−	−	+
*PPP2R5D* ^121^	61	M	−	−	+
*RAB39B* ^122^	48	M	+	+	+

Abbreviations: −, absent; + *, neurites (unspecified); +, present; BPAN, beta‐propeller protein associated neurodegeneration; CTX, cerebrotendinous xanthomatosis; F, female; LB, Lewy bodies; LN, Lewy neurites; M, male; MTHFR, 5,10‐methylenetetrahydrofolate reductase; NR, not reported; y, years.

## Discussion

**FIG. 3:**
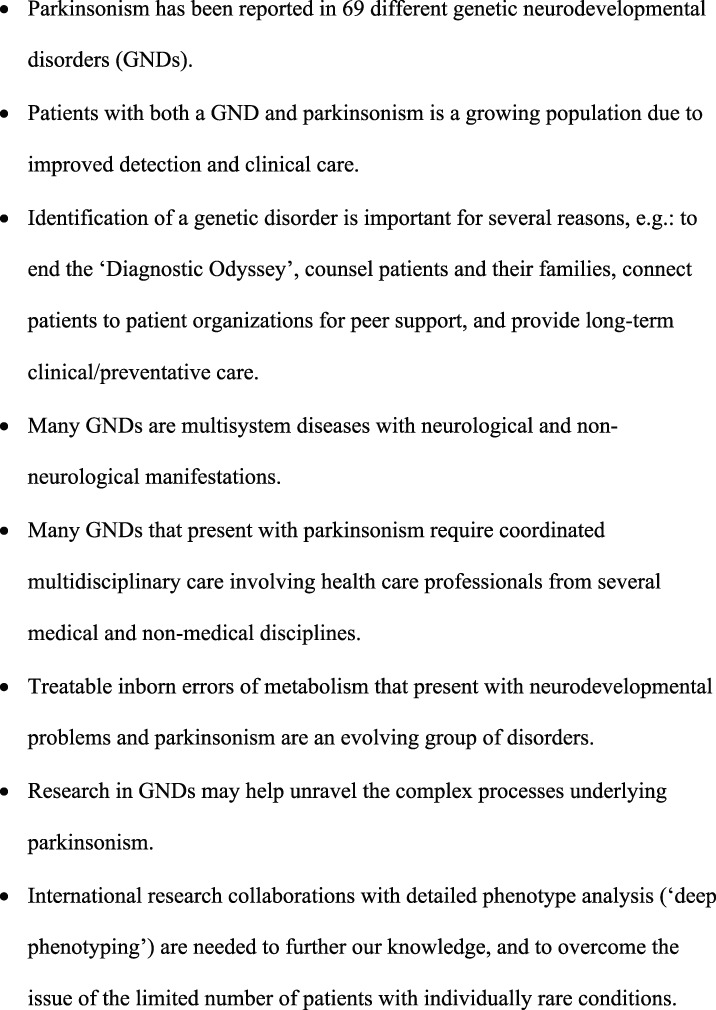
Main messages of the review.

In this first systematic literature review on parkinsonism in patients with GNDs, we provide a comprehensive overview of phenotypic characteristics and proposed pathophysiology in 69 different GNDs, based on a total of 422 patients. The main messages and implications of this review can be found in Figure 3.

Parkinsonism has been reported in a growing number of GNDs. This number may be expected to further increase given advances in clinical genetic testing and an increase in life expectancy because of improved medical care for patients with GNDs. Parkinsonism in these populations, however, appears to be under recognized in clinical practice and to be an understudied research topic.^14^, ^15^, ^16^ An explanation may be that, on the one hand, patients with GNDs may not always be aware of motor symptoms and may not be capable to express their symptoms adequately. Professionals, on the other hand, may attribute motor symptoms to manifestations of the GND, antipsychotic medication‐induced parkinsonism, or co‐existing neurologic disorders, whereas in fact they may miss a treatable condition of parkinsonism.^17^

### Phenotypic Characteristics

Many patients showed the typical phenotypic characteristics found for patients with Parkinson's disease: presence of cardinal motor signs, a good response to antiparkinsonian medication, and reduced DAT binding with dopaminergic imaging. However, there were some notable findings. For example, almost half of the patients included in our review were female, atypical for idiopathic Parkinson's disease sex distribution,^18^ and the median age at motor onset was 26 years, much younger compared to age‐related parkinsonism in the general population.^19^, ^20^ Many patients had co‐existing neurologic disorders, of which dystonia, seizures, and ataxia were most common.^21^, ^22^

Although previous research has suggested that patients with neurodevelopmental disorders may be susceptible to drug‐induced parkinsonism,^14^, ^23^ drug‐induced parkinsonism as the main cause was reported in only two patients.

### Pathophysiologic Mechanisms

The group of GNDs in this review was diverse, consistent with the multiple pathways and mechanisms involved in the pathogenesis of both neurodevelopmental and neurodegenerative disorders, including autophagic, lysosomal and mitochondrial function, endosomal trafficking, and the ubiquitin‐proteasome system.^24^, ^25^, ^26^ Dysregulation of these cellular processes, that are especially important in long‐lived cells such as neurons, may affect neurogenesis,^26^, ^27^ synaptic function,^28^, ^29^ neuroplasticity,^30^ and neuronal survival increasing the vulnerability for neurological and psychiatric disorders across a patient's lifespan.^3^, ^31^ These mechanisms may interact and when disrupted, contribute to a vicious cycle including the formation of protein aggregates, as has been implicated in Parkinson's disease.^32^, ^33^ Studying parkinsonism in patients with GNDs, and related animal models, may be useful to unravel the complex processes underlying parkinsonism.

Interestingly, in almost all GNDs with available data on neuropathology, neuronal loss was found, consistent with growing recognition that neurodevelopmental and neurodegenerative disorders overlap, rather than should be seen as opposite conditions.^2^, ^3^

### Clinical Implications

The large number of GNDs that may present with parkinsonism prompts questions about prevention, diagnosis, and management. Given the young onset and the co‐occurrence of other movement disorders seen in GNDs, early motor signs should be carefully monitored in patients with these conditions. Periodic standardized motor evaluations (eg, Movement Disorder Society‐Unified Parkinson's Disease Rating Scale [MDS‐UPDRS] and video‐recordings), and involvement of a movement disorder specialist may be considered. When parkinsonism presents and progresses, dopaminergic imaging may assist distinguishing medication‐induced parkinsonism from degenerative parkinsonism, which may be particularly difficult in patients with GNDs and complex neuropsychiatric expression.^34^

Clinicians treating patients with early‐onset parkinsonism and intellectual disabilities or other neurodevelopmental problems, should consider ordering a genetic test. Identification of an underlying diagnosis can be important for long‐term clinical management. It allows the opportunity for preventive and treatment strategies, may reduce the burden on patients and their families searching for answers (sometimes referred to as the “Diagnostic Odyssey”), inform disease risks for family members, and help patients and their families connecting with patient organizations for peer support.^35^ It is important to realize that patients with GNDs may develop multiple early‐ and late‐onset comorbidities that require proactive attention depending on the condition, which are typically not restricted to neurological problems, but may involve all body systems.^36^, ^37^ Increasingly, expert clinics specializing in specific GNDs are available, typically providing multidisciplinary care to improve patient outcomes and quality of life.

Next generation sequencing techniques, such as whole exome sequencing (WES), which may reveal a monogenic disorder,^35^ or chromosomal microarray analysis, that identifies genome wide CNVs,^38^ may be used as first line diagnostic tests. Certain features, including congenital anomalies and “dysmorphic facial features,” may point to the presence of a specific syndrome, but are absent in many patients. Here, it should also be noted that conventional karyotyping will not detect CNVs or monogenic disorders and that most currently available Parkinson's disease genetic diagnostic panels typically do not include GNDs. Therefore, an additional panel for intellectual disability may need to be ordered. It should also be realized that family history is often not a good predictor and many patients with a GND have a de novo mutation. Selecting the most appropriate genetic test can however be difficult and choosing the right test can require consultation with a clinical geneticist. Limitations of most genetic tests include the high costs and these tests are not readily available for many people, or inaccessible.

With recent advances in diagnosis and treatment of GNDs, targeted disease‐modifying therapies have become available for an increasing number of patients diagnosed with a GND (ie, for those with an inborn error of metabolism or tuberous sclerosis complex).^39^, ^40^, ^41^ Early detection of such disorders allows for timely interventions to prevent further brain damage and/or disease progression. Prominent examples of treatable inborn errors of metabolism in this review include phenylketonuria and other conditions that lead to disruptions in monoaminergic neurotransmitter metabolism (eg, *TH* and *DNAJC12*).^39^ These patients may receive great benefit from specific nutritional and/or pharmacological interventions with improvement in parkinsonism.^42^ Other examples include disorders of lipid metabolism (like cerebrotendinous xanthomatosis and X‐linked adrenoleukodystrophy) and 5,10‐methylenetetrahydrofolate reductase.^39^ Discoveries continue to be made regarding the development of treatment in inborn errors of metabolism that may have been associated with parkinsonism, making it an important and evolving group of disorders.

Management of parkinsonism requires a coordinated multidisciplinary approach in view of the comorbidity in many GNDs, which may involve clinical experts from many subspecialties in addition to the family doctor and movement disorder specialist.

### Research Implications

Phenotypic heterogeneity may be significant in GNDs given incomplete penetrance of genetic variants, variable expression, and pleiotropy,^43^ and may be particularly relevant in those GNDs involving multiple genes, such as is the case in 22q11.2 deletion syndrome. Detailed phenotype analysis (“deep‐phenotyping”) is crucial to further our knowledge on the complex relationships between genetics, pathophysiological mechanisms, environmental factors, and the phenotypic characteristics of parkinsonism in GNDs. International collaborative research is needed, to overcome the issue of the limited number of patients with individually rare conditions. In addition to routine assessments, including careful history taking that focusses on parkinsonian/neurological features and comorbid conditions of the genetic variant, and structured physical examinations (eg, MDS‐UPDRS), this should include careful family history taking. Periodic video assessments may also be considered. Despite the challenges, GNDs are often diagnosed at an early age, long before the onset of motor and non‐motor Parkinson's disease related symptoms, facilitating early‐stage research of parkinsonism. In addition to human studies, recognizing GNDs make it possible to use cell and animal models, available for many GNDs, to expand the possibilities of studying the pathophysiologic mechanisms, identify potential biomarkers, and design rational interventions.^44^, ^45^ Disease‐specific intervention strategies have been suggested in several human and animal models of GNDs and clearly demonstrate the benefits of these kinds of studies.^46^, ^47^

With an increased life expectancy for many GNDs, future research should also focus on overlap between GNDs and other neurodegenerative disorders (eg, major neurocognitive disorder) to understand shared underlying mechanisms and improve clinical care.

### Strengths and Limitations

The strengths of this systematic review include the pre‐registration of the protocol, the comprehensive search strategy, and the extensive data extraction on key characteristics of parkinsonism and its proposed pathophysiologic mechanisms. Several limitations should also be mentioned. The data need to be considered in view of the retrospective nature of most studies. Notably, given the large number and wide spectrum of genetically and clinically heterogeneous disorders, the absence of a perfect classification system that would prevent any inconsistency in inclusion/exclusion of reports, and differences in availability of information among genetic neurodevelopmental disorders, we cannot rule out the possibility of some inconsistencies in inclusion/exclusion of reports. Furthermore, because non‐English reports, studies lacking detailed information on the genetic disorder and/or criteria for parkinsonism, and studies reporting on genetic conditions with less than three reported cases were excluded, this review may not have captured all relevant publications. Moreover, because we included all conditions listed in HPO as “neurodevelopmental abnormality,” we may have included conditions that should not be considered to affect brain development. Heterogeneity in reporting made summarization of results difficult, hampering comparability between GND phenotypic characteristics. For example, the percentages depicted in the heatmap color scheme were based on the availability of data, which greatly varied from one report to the other (Fig. 1 and Fig. S2). Publication bias will have influenced the findings. Variable strength of the evidence linking genes to phenotypes and the preliminary nature of some findings should be taken into account.

## Conclusion

Parkinsonism has been reported in many GNDs. Findings from this study may provide clues for further research and improve management of patients with GNDs and/or parkinsonism.

## Author Roles

(1) Research project: A. Conception, B. Organization, C. Execution; (2) Statistical Analysis: A. Design, B. Execution, C. Review and Critique; (3) Manuscript: A. Writing of the First Draft, B. Review and Critique.

E.N.M.M.v.S.: 1A, 1B, 1C, 3A

E.B.: 1A, 1B, 1C, 3B

A.M.v.E.: 1A, 1C, 3B

T.J.d.K.: 3B

M.L.K.: 3B

J.R.Z.: 1C, 3B

A.R.M.: 1C, 3B

T.A.M.J.v.A: 1A, 3B

## Disclosures

### Ethical Compliance Statement

We confirm that we have read the Journal's position on issues involved in ethical publication and affirm that this work is consistent with those guidelines. The authors confirm that the approval of an institutional review board was not required for this work. The authors confirm that patient consent was not required for this work.

### Funding Sources and Conflict of Interest

This work was supported financially by Stichting Wetenschappelijk Onderzoek's Heeren Loo (2210100). The funder had no role in the design and conduct of the study, preparation of the review, or approval of the manuscript. The authors report no competing interests.

### Financial disclosures for the previous 12 months not related to this manuscript

E.B. has received compensation for clinical consultations for the Centre for Consultation and Expertise (CCE) and has received a research grant from the Dutch National Institutes of Health (ZonMw). T.J.d.K. is medical advisor for three non‐profit charities: Stofwisselingskracht foundation (Stichting Stofwisselingskracht), North Sea myoclonus foundation (Stichting Noordzeeziekte) and Janivo Foundation (Janivo Stichting). T.J.d.K. is also medical advisor of Ancora Health BV (profit), a Dutch company delivering lifestyle advice, of which he also holds shares. A.M.v.E is medical advisor of Jazz pharmaceuticals and received funding for "PCOM4RARE; Personalized Outcome Measures for patients with Intellectual Disability" from's Heeren Loo Care Group. J.R.Z. received a research grant from ZonMw and T.A.M.J.v.A. received a research grant from the National Institutes of Health. The other authors have no disclosures to report.

## Supporting information


**Appendix S1.** Additional details for the methods of this review.
**Figure S1**. Flow diagram depicting the different phases of the review.
**Figure S2.** Complete heat map with patient characteristics and parkinsonian features per genetic disorder
**Table S1.** List of included reports.
**Table S2.** List of included reports.
**Table S3.** Studies excluded from data‐extraction.
**Table S4.** Quality assessments of included studies.
**Table S5.** Quality assessments of included studies.

## Data Availability

The data that supports the findings of this study are available in the Supporting information of this article. The template that was used for data‐extraction is available on request.

## References

[1] Hickman RA , O'Shea SA , Mehler MF , Chung WK . Neurogenetic disorders across the lifespan: From aberrant development to degeneration. Nat Rev Neurol 2022;18(2):117–124. 10.1038/s41582-021-00595-5. 34987232 PMC10132523

[2] Saudubray JM , Garcia‐Cazorla A . An overview of inborn errors of metabolism affecting the brain: From neurodevelopment to neurodegenerative disorders. Dialogues Clin Neurosci 2018;20(4):301–325. 10.31887/DCNS.2018.20.4/jmsaudubray. 30936770 PMC6436954

[3] Morato Torres CA , Wassouf Z , Zafar F , Sastre D , Outeiro TF , Schüle B . The role of alpha‐Synuclein and other Parkinson's genes in neurodevelopmental and neurodegenerative disorders. Int J Mol Sci 2020;21(16):5724. 10.3390/ijms21165724. 32785033 PMC7460874

[4] Puschmann A . New genes causing hereditary Parkinson's disease or parkinsonism. Curr Neurol Neurosci Rep 2017;17(9):66. 10.1007/s11910-017-0780-8. 28733970 PMC5522513

[5] Morales‐Briceno H , Mohammad SS , Post B , Fois AF , Dale RC , Tchan M , VSC F . Clinical and neuroimaging phenotypes of genetic parkinsonism from infancy to adolescence. Brain 2019;143(3):751‐770. 10.1093/brain/awz345. 31800013

[6] Niemann N , Jankovic J . Juvenile parkinsonism: Differential diagnosis, genetics, and treatment. Parkinsonism Relat Disord 2019;67:74–89. 10.1016/j.parkreldis.2019.06.025. 31272925

[7] Wiseman FK , Al‐Janabi T , Hardy J , et al. A genetic cause of Alzheimer disease: Mechanistic insights from Down syndrome. Nat Rev Neurosci 2015;16(9):564–574. 10.1038/nrn3983. 26243569 PMC4678594

[8] Finucane BM , Myers SM , Martin CL , Ledbetter DH . Long overdue: Including adults with brain disorders in precision health initiatives. Curr Opin Genet Dev 2020;65:47–52. 10.1016/j.gde.2020.05.001. 32544666 PMC7736248

[9] Fortea J , Vilaplana E , Carmona‐Iragui M , et al. Clinical and biomarker changes of Alzheimer's disease in adults with down syndrome: A cross‐sectional study. Lancet 2020;395(10242):1988–1997. 10.1016/s0140-6736(20)30689-9. 32593336 PMC7322523

[10] Moher D , Shamseer L , Clarke M , et al. Preferred reporting items for systematic review and meta‐analysis protocols (PRISMA‐P) 2015 statement. Syst Rev 2015;4:1. 10.1186/2046-4053-4-1. 25554246 PMC4320440

[11] Köhler S , Carmody L , Vasilevsky N , et al. Expansion of the human phenotype ontology (HPO) knowledge base and resources. Nucleic Acids Res 2019;47(D1):D1018–d1027. 10.1093/nar/gky1105. 30476213 PMC6324074

[12] Postuma RB , Berg D , Stern M , et al. MDS clinical diagnostic criteria for Parkinson's disease. Mov Disord 2015;30(12):1591–1601. 10.1002/mds.26424. 26474316

[13] National Institutes of Health (2014). Study Quality Assessment Tools.. Accessed April, 2020. https://www.nhlbi.nih.gov/health-topics/study-quality-assessment-tools

[14] Scheifes A , Walraven S , Stolker JJ , Nijman HLI , Tenback DE , Egberts TCG , Heerdink ER . Movement disorders in adults with intellectual disability and behavioral problems associated with use of antipsychotics. J Clin Psychopharmacol 2016;36(4):308–313. 10.1097/jcp.0000000000000528. 27300250

[15] Boot E , Butcher NJ , Udow S , et al. Typical features of Parkinson disease and diagnostic challenges with microdeletion 22q11.2. Neurology 2018;90(23):e2059–e2067. 10.1212/wnl.0000000000005660. 29752303 PMC5993183

[16] Carvalho V , Ferreira JJ , Correia GL . Tremor and parkinsonism in Chromosomopathies—a systematic review. Mov Disord 2021;36(9):2017–2025. 10.1002/mds.28663. 34056754

[17] Butcher NJ , Kiehl TR , Hazrati LN , Chow EWC , Rogaeva E , Lang AE , Bassett AS . Association between early‐onset Parkinson disease and 22q11.2 deletion syndrome: Identification of a novel genetic form of Parkinson disease and its clinical implications. JAMA Neurol 2013;70(11):1359–1366. 10.1001/jamaneurol.2013.3646. 24018986 PMC4464823

[18] Gillies GE , Pienaar IS , Vohra S , Qamhawi Z . Sex differences in Parkinson's disease. Front Neuroendocrinol 2014;35(3):370–384. 10.1016/j.yfrne.2014.02.002. 24607323 PMC4096384

[19] Noyce AJ , Schrag A , Masters JM , Bestwick JP , Giovannoni G , Lees AJ . Subtle motor disturbances in PREDICT‐PD participants. J Neurol Neurosurg Psychiatry 2017;88(3):212–217. 10.1136/jnnp-2016-314524. 27986830 PMC5529958

[20] Rees RN , Noyce AJ , Schrag A . The prodromes of Parkinson's disease. Eur J Neurosci 2019;49(3):320–327. 10.1111/ejn.14269. 30447019 PMC6492156

[21] Tolosa E , Compta Y . Dystonia in Parkinson's disease. J Neurol 2006;253(Suppl 7):Vii7–Vii13. 10.1007/s00415-006-7003-6. 17131231

[22] Son AY , Biagioni MC , Kaminski D , Gurevich A , Stone B , di Rocco A . Parkinson's disease and cryptogenic epilepsy. Case Rep Neurol Med 2016;2016:3745631. 10.1155/2016/3745631. 27688919 PMC5027309

[23] Butcher NJ , Boot E , Lang AE , Andrade D , Vorstman J , McDonald‐McGinn D , Bassett AS . Neuropsychiatric expression and catatonia in 22q11.2 deletion syndrome: An overview and case series. Am J Med Genet A 2018;176(10):2146–2159. 10.1002/ajmg.a.38708. 29777584 PMC6209527

[24] Stavoe AKH , Holzbaur ELF . Autophagy in Neurons. Annu Rev Cell Dev Biol 2019;35:477–500. 10.1146/annurev-cellbio-100818-125242. 31340124 PMC6996145

[25] Luza S , Opazo CM , Bousman CA , Pantelis C , Bush AI , Everall IP . The ubiquitin proteasome system and schizophrenia. Lancet Psychiatry 2020;7(6):528–537. 10.1016/s2215-0366(19)30520-6. 32061320

[26] Khacho M , Harris R , Slack RS . Mitochondria as central regulators of neural stem cell fate and cognitive function. Nat Rev Neurosci 2019;20(1):34–48. 10.1038/s41583-018-0091-3. 30464208

[27] Tuoc TC , Stoykova A . Roles of the ubiquitin‐proteosome system in neurogenesis. Cell Cycle 2010;9(16):3174–3180. 10.4161/cc.9.16.12551. 20852390

[28] Ghavami S , Shojaei S , Yeganeh B , et al. Autophagy and apoptosis dysfunction in neurodegenerative disorders. Prog Neurobiol 2014;112:24–49. 10.1016/j.pneurobio.2013.10.004. 24211851

[29] Andres‐Alonso M , Kreutz MR , Karpova A . Autophagy and the endolysosomal system in presynaptic function. Cell Mol Life Sci 2021;78(6):2621–2639. 10.1007/s00018-020-03722-5. 33340068 PMC8004491

[30] Mattson MP , Gleichmann M , Cheng A . Mitochondria in neuroplasticity and neurological disorders. Neuron 2008;60(5):748–766. 10.1016/j.neuron.2008.10.010. 19081372 PMC2692277

[31] Zoghbi HY . Postnatal neurodevelopmental disorders: Meeting at the synapse? Science 2003;302(5646):826–830. 10.1126/science.1089071. 14593168

[32] Kurtishi A , Rosen B , Patil KS , Alves GW , Møller SG . Cellular Proteostasis in Neurodegeneration. Mol Neurobiol 2019;56(5):3676–3689. 10.1007/s12035-018-1334-z. 30182337

[33] Poewe W , Seppi K , Tanner CM , et al. Parkinson disease. Nat Rev Dis Primers 2017;3:17013. 10.1038/nrdp.2017.13. 28332488

[34] Catafau AM , Tolosa E . Impact of dopamine transporter SPECT using 123I‐Ioflupane on diagnosis and management of patients with clinically uncertain parkinsonian syndromes. Mov Disord 2004;19(10):1175–1182. 10.1002/mds.20112. 15390019

[35] Manickam K , McClain MR , Demmer LA , et al. Exome and genome sequencing for pediatric patients with congenital anomalies or intellectual disability: An evidence‐based clinical guideline of the American College of Medical Genetics and Genomics (ACMG). Genet Med 2021;23(11):2029–2037. 10.1038/s41436-021-01242-6. 34211152

[36] Crawford K , Bracher‐Smith M , Owen D , et al. Medical consequences of pathogenic CNVs in adults: Analysis of the UK Biobank. J Med Genet 2019;56(3):131–138. 10.1136/jmedgenet-2018-105477. 30343275

[37] van Eeghen AM , Bruining H , Wolf NI , Bergen AA , Houtkooper RH , van Haelst MM , van Karnebeek CD . Personalized medicine for rare neurogenetic disorders: can we make it happen? Cold Spring Harb Mol Case Stud 2022;8(2):a006200. 10.1101/mcs.a006200. PMC8958924 35332073

[38] Durmaz AA , Karaca E , Demkow U , Toruner G , Schoumans J , Cogulu O . Evolution of genetic techniques: Past, present, and beyond. Biomed Res Int 2015;2015:461524. 10.1155/2015/461524. 25874212 PMC4385642

[39] Hoytema van Konijnenburg EMM , Wortmann SB , Koelewijn MJ , et al. Treatable inherited metabolic disorders causing intellectual disability: 2021 review and digital app. Orphanet J Rare Dis 2021;16(1):170. 10.1186/s13023-021-01727-2. 33845862 PMC8042729

[40] Ebrahimi‐Fakhari D , van Karnebeek C , Münchau A . Movement disorders in treatable inborn errors of metabolism. Mov Disord 2019;34(5):598–613. 10.1002/mds.27568. 30557456

[41] Overwater IE , Rietman AB , van Eeghen AM , de Wit MCY . Everolimus for the treatment of refractory seizures associated with tuberous sclerosis complex (TSC): Current perspectives. Ther Clin Risk Manag 2019;15:951–955. 10.2147/tcrm.S145630. 31440057 PMC6666377

[42] Tufekcioglu Z , Cakar A , Bilgic B , Hanagasi H , Gurvit H , Emre M . Adult‐onset phenylketonuria with rapidly progressive dementia and parkinsonism. Neurocase 2016;22(3):273–275. 10.1080/13554794.2016.1142567. 26962957

[43] Magrinelli F , Balint B , Bhatia KP . Challenges in Clinicogenetic correlations: One gene—many phenotypes. Mov Disord Clin Pract 2021;8(3):299–310. 10.1002/mdc3.13165. 33816657 PMC8015894

[44] Decressac M , Volakakis N , Björklund A , Perlmann T . NURR1 in Parkinson disease‐‐from pathogenesis to therapeutic potential. Nat Rev Neurol 2013;9(11):629–636. 10.1038/nrneurol.2013.209. 24126627

[45] Luoma P , Melberg A , Rinne JO , et al. Parkinsonism, premature menopause, and mitochondrial DNA polymerase gamma mutations: Clinical and molecular genetic study. Lancet 2004;364(9437):875–882. 10.1016/s0140-6736(04)16983-3. 15351195

[46] Manti F , Nardecchia F , Barresi S , et al. Neurotransmitter trafficking defect in a patient with clathrin (CLTC) variation presenting with intellectual disability and early‐onset parkinsonism. Parkinsonism Relat Disord 2019;61:207–210. 10.1016/j.parkreldis.2018.10.012. 30337205

[47] Straniero L , Guella I , Cilia R , et al. DNAJC12 and dopa‐responsive nonprogressive parkinsonism. Ann Neurol 2017;82(4):640–646. 10.1002/ana.25048. 28892570

[48] Zschocke J . HSD10 disease: Clinical consequences of mutations in the HSD17B10 gene. J Inherit Metab Dis 2012;35(1):81–89. 10.1007/s10545-011-9415-4. 22127393

[49] Lake NJ , Bird MJ , Isohanni P , Paetau A . Leigh syndrome: Neuropathology and pathogenesis. J Neuropathol Exp Neurol 2015;74(6):482–492. 10.1097/nen.0000000000000195. 25978847

[50] Trifunovic A , Wredenberg A , Falkenberg M , et al. Premature ageing in mice expressing defective mitochondrial DNA polymerase. Nature 2004;429(6990):417–423. 10.1038/nature02517. 15164064

[51] Rahman S , Copeland WC . POLG‐related disorders and their neurological manifestations. Nat Rev Neurol 2019;15(1):40–52. 10.1038/s41582-018-0101-0. 30451971 PMC8796686

[52] Burke EA , Frucht SJ , Thompson K , et al. Biallelic mutations in mitochondrial tryptophanyl‐tRNA synthetase cause levodopa‐responsive infantile‐onset parkinsonism. Clin Genet 2018;93(3):712–718. 10.1111/cge.13172. 29120065 PMC5828974

[53] Maynard TM , Meechan DW , Dudevoir ML , et al. Mitochondrial localization and function of a subset of 22q11 deletion syndrome candidate genes. Mol Cell Neurosci 2008;39(3):439–451. 10.1016/j.mcn.2008.07.027. 18775783 PMC2729512

[54] Boot E , Booij J , Zinkstok J , et al. Disrupted dopaminergic neurotransmission in 22q11 deletion syndrome. Neuropsychopharmacology 2008;33(6):1252–1258. 10.1038/sj.npp.1301508. 17653112

[55] Devaraju P , Zakharenko SS . Mitochondria in complex psychiatric disorders: Lessons from mouse models of 22q11.2 deletion syndrome: Hemizygous deletion of several mitochondrial genes in the 22q11.2 genomic region can lead to symptoms associated with neuropsychiatric disease. Bioessays 2017;39(2). 10.1002/bies.201600177. PMC5304421 28044359

[56] Boot E , Bassett AS , Marras C . 22q11.2 deletion syndrome‐associated Parkinson's disease. Mov Disord Clin Pract 2019;6(1):11–16. 10.1002/mdc3.12687. 30746410 PMC6335527

[57] Vacca RA , Bawari S , Valenti D , et al. Down syndrome: Neurobiological alterations and therapeutic targets. Neurosci Biobehav Rev 2019;98:234–255. 10.1016/j.neubiorev.2019.01.001. 30615933

[58] Jiang Y , Sato Y , Im E , et al. Lysosomal dysfunction in down syndrome is APP‐dependent and mediated by APP‐βCTF (C99). J Neurosci 2019;39(27):5255–5268. 10.1523/jneurosci.0578-19.2019. 31043483 PMC6607756

[59] Valenti D , Tullo A , Caratozzolo MF , Merafina RS , Scartezzini P , Marra E , Vacca RA . Impairment of F1F0‐ATPase, adenine nucleotide translocator and adenylate kinase causes mitochondrial energy deficit in human skin fibroblasts with chromosome 21 trisomy. Biochem J 2010;431(2):299–310. 10.1042/bj20100581. 20698827

[60] Lanoue V , Chai YJ , Brouillet JZ , Weckhuysen S , Palmer EE , Collins BM , Meunier FA . STXBP1 encephalopathy: Connecting neurodevelopmental disorders with alpha‐synucleinopathies? Neurology 2019;93(3):114–123. 10.1212/wnl.0000000000007786. 31221716

[61] Jafari P , Braissant O , Bonafé L , Ballhausen D . The unsolved puzzle of neuropathogenesis in glutaric aciduria type I. Mol Genet Metab 2011;104(4):425–437. 10.1016/j.ymgme.2011.08.027. 21944461

[62] van der Burgh R , Ter Haar NM , Boes ML , Frenkel J . Mevalonate kinase deficiency, a metabolic autoinflammatory disease. Clin Immunol 2013;147(3):197–206. 10.1016/j.clim.2012.09.011. 23110805

[63] Zyss J , Vlaicu M , Gerber S , Rodallec M , Gauthier C , Zuber M . Movement disorders in mevalonic aciduria. Mov Disord 2011;26(SUPPL. 2):S256. 15th International Congress of Parkinson's Disease and Movement Disorders. Toronto, ON Canada. (var.pagings). 10.1002/mds.23764.

[64] Marin‐Valencia I , Roe CR , Pascual JM . Pyruvate carboxylase deficiency: Mechanisms, mimics and anaplerosis. Mol Genet Metab 2010;101(1):9–17. 10.1016/j.ymgme.2010.05.004. 20598931

[65] Kawaguchi K . Role of kinesin‐1 in the pathogenesis of SPG10, a rare form of hereditary spastic paraplegia. Neuroscientist 2013;19(4):336–344. 10.1177/1073858412451655. 22785106

[66] Tang G , Yue Z , Talloczy Z , et al. Autophagy induced by Alexander disease‐mutant GFAP accumulation is regulated by p38/MAPK and mTOR signaling pathways. Hum Mol Genet 2008;17(11):1540–1555. 10.1093/hmg/ddn042. 18276609 PMC2902290

[67] Hor CHH , Tang BL . Beta‐propeller protein‐associated neurodegeneration (BPAN) as a genetically simple model of multifaceted neuropathology resulting from defects in autophagy. Rev Neurosci 2019;30(3):261–277. 10.1515/revneuro-2018-0045. 30204590

[68] Xu M , Ouyang Q , Gong J , et al. Mixed Neurodevelopmental and Neurodegenerative Pathology in Nhe6‐Null Mouse Model of Christianson Syndrome. eNeuro 2017;4(6):e0388‐17.2017. 10.1523/eneuro.0388-17.2017. PMC5771691 29349289

[69] Strømme P , Dobrenis K , Sillitoe RV , et al. X‐linked Angelman‐like syndrome caused by Slc9a6 knockout in mice exhibits evidence of endosomal‐lysosomal dysfunction. Brain 2011;134(Pt 11):3369–3383. 10.1093/brain/awr250. 21964919 PMC3212719

[70] Zadori D , Szalardy L , Maszlag‐Torok R , et al. Clinicopathological relationships in an aged case of DOORS syndrome with a p.Arg506X mutation in the ATP6V1B2 gene. Front Neurol 2020;11:767. 10.3389/fneur.2020.00767. 32849222 PMC7427051

[71] Yasa S , Modica G , Sauvageau E , Kaleem A , Hermey G , Lefrancois S . CLN3 regulates endosomal function by modulating Rab7A‐effector interactions. J Cell Sci 2020;133(6):jcs234047. 10.1242/jcs.234047. 32034082

[72] Schweizer M , Markmann S , Braulke T , Kollmann K . Ultrastructural analysis of neuronal and non‐neuronal lysosomal storage in mucolipidosis type II knock‐in mice. Ultrastruct Pathol 2013;37(5):366–372. 10.3109/01913123.2013.810687. 24047352

[73] Yu SH , Wang T , Wiggins K , et al. Lysosomal cholesterol accumulation contributes to the movement phenotypes associated with NUS1 haploinsufficiency. Genet Med 2021;23(7):1305–1314. 10.1038/s41436-021-01137-6. 33731878 PMC8263489

[74] Tang BL . Rabs, membrane dynamics, and Parkinson's disease. J Cell Physiol 2017;232(7):1626–1633. 10.1002/jcp.25713. 27925204

[75] Koss DJ , Campesan S , Giorgini F , Outeiro TF . Dysfunction of RAB39B‐mediated vesicular trafficking in Lewy body diseases. Mov Disord 2021;36:1744–1758. 10.1002/mds.28605. 33939203

[76] Renvoisé B , Chang J , Singh R , et al. Lysosomal abnormalities in hereditary spastic paraplegia types SPG15 and SPG11. Ann Clin Transl Neurol 2014;1(6):379–389. 10.1002/acn3.64. 24999486 PMC4078876

[77] Pozner T , Regensburger M , Engelhorn T , Winkler J , Winner B . Janus‐faced spatacsin (SPG11): Involvement in neurodevelopment and multisystem neurodegeneration. Brain 2020;143(8):2369–2379. 10.1093/brain/awaa099. 32355960 PMC7447516

[78] Fernandes Filho JA , Shapiro BE . Tay‐Sachs disease. Arch Neurol 2004;61(9):1466–1468. 10.1001/archneur.61.9.1466. 15364698

[79] Dubos A , Castells‐Nobau A , Meziane H , et al. Conditional depletion of intellectual disability and parkinsonism candidate gene ATP6AP2 in fly and mouse induces cognitive impairment and neurodegeneration. Hum Mol Genet 2015;24(23):6736–6755. 10.1093/hmg/ddv380. 26376863 PMC4634377

[80] Desplats P , Patel P , Kosberg K , et al. Combined exposure to Maneb and Paraquat alters transcriptional regulation of neurogenesis‐related genes in mice models of Parkinson's disease. Mol Neurodegener 2012;7:49. 10.1186/1750-1326-7-49. 23017109 PMC3502617

[81] Fan SP , Lee NC , Lin CH . Novel phenotype of 6p25 deletion syndrome presenting juvenile parkinsonism and brain calcification. Mov Disord 2020;35:1457–1462. 10.1002/mds.28079. 32369633

[82] Porta F , Mussa A , Concolino D , Spada M , Ponzone A . Dopamine agonists in dihydropteridine reductase deficiency. Mol Genet Metab 2012;105(4):582–584. 10.1016/j.ymgme.2012.01.013. 22325981

[83] Bouchereau J , Huttlin EL , Guarani V , Pichard S , Anikster Y , Schiff M . DNAJC12: A molecular chaperone involved in proteostasis, PKU, biogenic amines metabolism and beyond? Mol Genet Metab 2018;123(3):285–286. 10.1016/j.ymgme.2018.01.006. 29396030

[84] Ng J , Barral S , de La Fuente BC , et al. Gene therapy restores dopamine transporter expression and ameliorates pathology in iPSC and mouse models of infantile parkinsonism. Sci Transl Med 2021;13(594):eaaw1564. 10.1126/scitranslmed.aaw1564. PMC7612279 34011628

[85] Catterall WA . Dravet syndrome: A Sodium Channel Interneuronopathy. Curr Opin Physiol 2018;2:42–50. 10.1016/j.cophys.2017.12.007. 30123852 PMC6091224

[86] Tassin J , Durr A , Bonnet AM , et al. Levodopa‐responsive dystonia. GTP cyclohydrolase I or parkin mutations? Brain 2000;123(Pt 6):1112–1121. 10.1093/brain/123.6.1112. 10825351

[87] Bergoug M , Doudeau M , Godin F , Mosrin C , Vallée B , Bénédetti H . Neurofibromin Structure, Functions and Regulation. Cells 2020;9(11):2365. 10.3390/cells9112365. PMC7692384 33121128

[88] Curtius HC , Niederwieser A , Viscontini M , et al. Serotonin and dopamine synthesis in phenylketonuria. Adv Exp Med Biol 1981;133:277–291. 10.1007/978-1-4684-3860-4_16. 6119011

[89] Boot E , Hollak CEM , Huijbregts SCJ , et al. Cerebral dopamine deficiency, plasma monoamine alterations and neurocognitive deficits in adults with phenylketonuria. Psychol Med 2017;47(16):2854–2865. 10.1017/s0033291717001398. 28552082

[90] Hetzelt K , Kerling F , Kraus C , et al. Early‐onset parkinsonism in PPP2R5D‐related neurodevelopmental disorder. Eur J Med Genet 2021;64(1):104123. 10.1016/j.ejmg.2020.104123. 33338668

[91] Peng XM , Tehranian R , Dietrich P , Stefanis L , Perez RG . α‐Synuclein activation of protein phosphatase 2A reduces tyrosine hydroxylase phosphorylation in dopaminergic cells. J Cell Sci 2005;118(15):3523–3530. 10.1242/jcs.02481. 16030137

[92] Zielonka M , Makhseed N , Blau N , Bettendorf M , Hoffmann GF , Opladen T . Dopamine‐responsive growth‐hormone deficiency and central hypothyroidism in Sepiapterin reductase deficiency. JIMD Rep 2015;24:109–113. 10.1007/8904_2015_450. 26006722 PMC4582026

[93] de Rijk‐Van Andel JF , Gabreels FJ , Geurtz B , et al. L‐dopa‐responsive infantile hypokinetic rigid parkinsonism due to tyrosine hydroxylase deficiency. Neurology 2000;55(12):1926–1928. 10.1212/wnl.55.12.1926. 11134401

[94] Manti F , Barresi S , Venditti M , et al. Neurotransmitter trafficking defect in a patient with the clathrin (CLTC) alteration presenting with hyperphenylalaninemia and Parkinsonism. Journal of Inherited Metabolic Disease 2018;41(Supplement 1):S42–S43. 56th Annual Symposium of the Society for the Study of Inborn Errors of Metabolism, SSIEM 2018. Greece. 10.1007/s10545-018-0233-9.

[95] Gorenberg EL , Chandra SS . The role of co‐chaperones in synaptic Proteostasis and neurodegenerative disease. Front Neurosci 2017;11:248. 10.3389/fnins.2017.00248. 28579939 PMC5437171

[96] Fasano D , Parisi S , Pierantoni GM , et al. Alteration of endosomal trafficking is associated with early‐onset parkinsonism caused by SYNJ1 mutations. Cell Death Dis 2018;9(3):385. 10.1038/s41419-018-0410-7. 29515184 PMC5841278

[97] Matsuura T , Sutcliffe JS , Fang P , et al. De novo truncating mutations in E6‐AP ubiquitin‐protein ligase gene (UBE3A) in Angelman syndrome. Nat Genet 1997;15(1):74–77. 10.1038/ng0197-74. 8988172

[98] Mulherkar SA , Sharma J , Jana NR . The ubiquitin ligase E6‐AP promotes degradation of alpha‐synuclein. J Neurochem 2009;110(6):1955–1964. 10.1111/j.1471-4159.2009.06293.x. 19645749

[99] Namihira T , Hattori N , Shiroma S , Miyazato Y . Autosomal recessive juvenile Parkinson's disease with partial trisomy of chromosome 6q syndrome: A case report. Psychiatry Clin Neurosci 2004;58(6):672–673. 10.1111/j.1440-1819.2004.01321.x. 15601396

[100] Fishman PS , Oyler GA . Significance of the parkin gene and protein in understanding Parkinson's disease. Curr Neurol Neurosci Rep 2002;2(4):296–302. 10.1007/s11910-002-0004-7. 12044248

[101] Ishii T , Funakoshi M , Kobayashi H . Yeast Pth2 is a UBL domain‐binding protein that participates in the ubiquitin‐proteasome pathway. Embo j 2006;25(23):5492–5503. 10.1038/sj.emboj.7601418. 17082762 PMC1679763

[102] Pilo de la Fuente B , Ruiz I , Lopez de Munain A , Jimenez‐Escrig A . Cerebrotendinous xanthomatosis: neuropathological findings. J Neurol 2008;255(6):839–842. 10.1007/s00415-008-0729-6. 18458861

[103] Chen JY , Oza VS , Gopi R , Christine CW . Rapidly progressive Parkinsonism in a patient with incontinentia pigmenti. Movement Disorders 2015;30(SUPPL. 1):S476–S477. 19th International Congress of Parkinson's Disease and Movement Disorders. San Diego, CA United States. (var.pagings). 10.1002/mds.26295.

[104] Tamtaji OR , Reiter RJ , Alipoor R , Dadgostar E , Kouchaki E , Asemi Z . Melatonin and Parkinson disease: Current status and future perspectives for molecular mechanisms. Cell Mol Neurobiol 2020;40(1):15–23. 10.1007/s10571-019-00720-5. 31388798 PMC11448849

[105] Lee KS , Lee JE , Cho AH , Kim JS , Oh YS . A case of Parkinson's disease in a patient with Klinefelter's syndrome. Acta Neurol Belg 2019;120:971–972. 10.1007/s13760-019-01232-1. 31667793

[106] Ma S , Sun R , Jiang B , et al. *L2hgdh* Deficiency Accumulates l‐2‐Hydroxyglutarate with Progressive Leukoencephalopathy and Neurodegeneration. Mol Cell Biol 2017;37(8):e00492‐16. 10.1128/mcb.00492-16. PMC5376639 28137912

[107] Buongarzone G , Minafra B , Errichiello E , et al. 13.3.1. Movement disorders in a family carrying ATP7A variant. Movement Disorders 2020;35(SUPPL 1):S89. MDS International Congress. Virtual. 10.1002/mds.28268.

[108] Vulpe C , Levinson B , Whitney S , Packman S , Gitschier J . Isolation of a candidate gene for Menkes disease and evidence that it encodes a copper‐transporting ATPase. Nat Genet 1993;3(1):7–13. 10.1038/ng0193-7. 8490659

[109] Alkufri F , Harrower T , Rahman Y , et al. Molybdenum cofactor deficiency presenting with a parkinsonism‐dystonia syndrome. Mov Disord 2013;28(3):399–401. 10.1002/mds.25276. 23436702

[110] Jakubiczka‐Smorag J , Santamaria‐Araujo JA , Metz I , et al. Mouse model for molybdenum cofactor deficiency type B recapitulates the phenotype observed in molybdenum cofactor deficient patients. Hum Genet 2016;135(7):813–826. 10.1007/s00439-016-1676-4. 27138983

[111] Garraux G , Caberg JH , Vanbellinghen JF , Jamar M , Bours V , Moonen G , Dive D . Partial trisomy 4q associated with young‐onset dopa‐responsive parkinsonism. Arch Neurol 2012;69(3):398–400. 10.1001/archneurol.2011.802. 22410449

[112] Morales‐Briceno H , Ha AD , London K , Farlow D , Chang FCF , Fung VSC . Parkinsonism in PGK1 deficiency implicates the glycolytic pathway in nigrostriatal dysfunction. Parkinsonism Relat Disord 2019;64:319–323. 10.1016/j.parkreldis.2019.04.004. 30975619

[113] Shakkottai VG , Xiao M , Xu L , Wong M , Nerbonne JM , Ornitz DM , Yamada KA . FGF14 regulates the intrinsic excitability of cerebellar Purkinje neurons. Neurobiol Dis 2009;33(1):81–88. 10.1016/j.nbd.2008.09.019. 18930825 PMC2652849

[114] Poisson A , Nicolas A , Bousquet I , Raverot V , Gronfier C , Demily C . Smith‐Magenis Syndrome: Molecular Basis of a Genetic‐Driven Melatonin Circadian Secretion Disorder. Int J Mol Sci 2019;20(14):3533. 10.3390/ijms20143533. PMC6679101 31330985

[115] Hayflick SJ , Kruer MC , Gregory A , et al. Beta‐propeller protein‐associated neurodegeneration: A new X‐linked dominant disorder with brain iron accumulation. Brain 2013;136(Pt 6):1708–1717. 10.1093/brain/awt095. 23687123 PMC3673459

[116] Fernandez HH , Friedman JH , Famiglietti EV . Probable Cornelia de Lange syndrome with progressive parkinsonism and dystonia. Mov Disord 2000;15(4):749–751. 10.1002/1531-8257(200007)15:4<749::aid-mds1028>3.0.co;2-p. 10928594

[117] Bodhireddy S , Dickson DW , Mattiace L , Weidenheim KM . A case of Down's syndrome with diffuse Lewy body disease and Alzheimer's disease. Neurology 1994;44(1):159–161. 10.1212/wnl.44.1.159. 8290054

[118] Marui W , Iseki E , Kosaka K , Kato M , Adachi Y , Ueda K . An autopsied case of down syndrome with Alzheimer pathology and alpha‐synuclein immunoreactivity. Neuropathology 1999;19(4):410–416. 10.1046/j.1440-1789.1999.00265.x.

[119] Clayton PT , Smith I , Harding B . Subacute combined degeneration of the cord, dementia and parkinsonism due to an inborn error of folate metabolism. J Neurol Neurosurg Psychiatry 1986;49(8):920–927. 3755752 10.1136/jnnp.49.8.920 PMC1028954

[120] Sleiman PM , Healy DG , Muqit MM , et al. Characterisation of a novel NR4A2 mutation in Parkinson's disease brain. Neurosci Lett 2009;457(2):75–79. 10.1016/j.neulet.2009.03.021. 19429166 PMC4763922

[121] Kim CY , Wirth T , Hubsch C , et al. Early‐onset parkinsonism is a manifestation of the PPP2R5D p.E200K mutation. Ann Neurol 2020;88(5):1028–1033. 10.1002/ana.25863. 32743835 PMC9052555

[122] Wilson GR , Sim JC , McLean C , et al. Mutations in RAB39B cause X‐linked intellectual disability and early‐onset Parkinson disease with alpha‐synuclein pathology. Am J Hum Genet 2014;95(6):729–735. 10.1016/j.ajhg.2014.10.015. 25434005 PMC4259921

