# Supplementary material for: Parkinsonism in Genetic Neurodevelopmental Disorders: A Systematic Review
Source: Mov Disord Clin Pract. 2022 Oct 31;10(1):17–31. doi: 10.1002/mdc3.13577 (PMC9847320; doi:10.1002/mdc3.13577)
Supplement: Supplementary file 1 — Appendix S1. Additional details for the methods of this review. Figure S1. Flow diagram depicting the different phases of the review. Figure S2. Complete heat map with patient characteristics and parkinsonian features per genetic disorder Table S1. List of included reports. Table S2. List of included reports. Table S3. Studies excluded from data‐extraction. Table S4. Quality assessments of included studies. Table S5. Quality assessments of included studies. [file MDC3-10-17-s001.docx]

Index Supplemental Material

[sMethods 1. Search strategy 2](#_Toc112664393)

[**PubMed** 2](#_Toc112664394)

[**Embase** 2](#_Toc112664395)

[Figure S1. Flow diagram depicting the different phases of the systematic review 4](#_Toc112664396)

[Figure S2. Patient characteristics and parkinsonian features per genetic disorder 5](#_Toc112664397)

[sTable 1. List of included reports describing data on an individual level 9](#_Toc112664398)

[sTable 2. List of included reports describing data at group level 22](#_Toc112664399)

[sTable 3. Studies excluded from data-extraction 25](#_Toc112664400)

[sTable 4. Quality assessment of observational cohort and cross-sectional studies 30](#_Toc112664401)

[sTable 5. Quality assessment of case-control studies 31](#_Toc112664402)

[sReferences 32](#_Toc112664403)

# **sMethods 1. Search strategy**

**Description**

General search terms related to “genetic disease”, “neurodevelopmental disorder” and “Parkinson’s disease/parkinsonism” were included in the search. Mesh terms in the first subheading of “genetic disease, inborn” were included if they have been associated with a neurodevelopmental disorder. General search terms were combined with a list of genetic disorders associated with a neurodevelopmental abnormality. This list of search terms was composed using the human phenotype ontology (HPO) database on <http://www.orphadata.org/cgi-bin/index.php>. All terms describing a rare genetic disease assigned to the subontology neurodevelopmental abnormality, defined by HPO as ‘*A deviation from normal of the neurological development of a child, which may include any or all of the aspects of the development of personal, social, gross or fine motor, and cognitive abilities*’ were included; collectively, the ‘HPO/ND-list’.

## **PubMed**

**General search terms**

***Parkinson/parkinsonism***

"Parkinsonian Disorders"[Mesh] OR Parkinson*[tiab] OR "lewy body dementia"[tiab] OR lewy body disease*[tiab]

AND

***Genetic disease/neurodevelopemental disorder***

"Genetic Diseases, Inborn"[Mesh:NoExp] OR genetic disease*[tiab] OR genetic disorder*[tiab] OR hereditary disease*[tiab] OR hereditary disorder*[tiab] OR single-gene defect*[tiab] OR "Chromosome Disorders"[Mesh] OR chromosome disorder*[tiab] OR chromosomal disorder*[tiab] OR "Metabolism, Inborn Errors"[Mesh] OR inborn error metabolism*[tiab] OR inborn metabolism error*[tiab] OR "Intellectual Disability"[Mesh] OR intellectual disabilit*[tiab] OR mental retardation*[tiab] OR Intellectual Development Disorder*[tiab] OR Intellectual Developmental Disorder*[tiab] OR mental deficienc*[tiab] OR neurodevelopmental delay[tiab] OR neurodevelopment delay[tiab] OR "Alagille Syndrome"[Mesh] OR syndrome Alagille*[tiab] OR Alagille syndrome*[tiab] OR Arteriohepatic Dysplasia*[tiab] OR Cardiovertebral Syndrome*[tiab] OR Arteriohepatic Dysplasia*[tiab] OR Watson miller syndrome*[tiab] OR Hepatic Ductular Hypoplasia*[tiab] OR alagille-watson*[tiab] OR "CHARGE Syndrome"[Mesh] OR CHARGE syndrome*[tiab] OR hall hittner syndrome*[tiab] OR Charge association*[tiab] OR "Costello Syndrome"[Mesh] OR Costello syndrome*[tiab] OR FCS syndrome*[tiab] OR Faciocutaneoskeletal Syndrome*[tiab] OR "Genetic Diseases, X-Linked"[Mesh] OR X-linked genetic disease*[tiab] OR "Lennox Gastaut Syndrome"[Mesh] OR lennox gastaut syndrome*[tiab] OR "Oculocerebrorenal Syndrome"[Mesh] OR lowe syndrome*[tiab] OR oculocerebrorenal syndrome*[tiab] OR Lowe Bickel Syndrome*[tiab] OR lowe diseas*[tiab] OR "Orofaciodigital Syndromes"[Mesh] OR orofaciodigital syndrome*[tiab] OR Dysplasia Linguofacialis[tiab] OR Gorlin Psaume Syndrome*[tiab] OR Mohr Syndrome*[tiab] OR "Ataxia Telangiectasia"[Mesh] OR ataxia telangiectas*[tiab] OR Louis Bar Syndrome*[tiab] OR "Pain Insensitivity, Congenital"[Mesh] OR Congenital Analgesia*[tiab] OR "Congenital Insensitivity To Pain"[tiab] OR Congenital Pain Indifference*[tiab] OR Congenital Pain Insensitivit*[tiab] OR "Congenital Indifference to Pain"[tiab] **OR**  **#(list ND/HPO for PubMed)**

NOT

"Wolff-Parkinson-White Syndrome"[Mesh] OR "wolff-parkinson-white" OR WPW syndrome* OR "Fragile X Tremor Ataxia Syndrome"[Supplementary Concept] OR "Fragile X Tremor Ataxia" OR "FXTAS" OR "premutation" OR "Gaucher" OR "Gaucher Disease"[Mesh] OR Glucocerebrosidase deficienc* OR GBA deficienc* OR Wilson’s dis* OR Wilson dis* OR "Hepatolenticular Degeneration"[Mesh] OR "hepatolenticular Degeneration"

## **Embase**

**General search terms**

***Parkinson/parkinsonism***

*Parkinson disease/ or *parkinsonism/ or Parkinson*.ti,ab,kw.

AND

***Genetic disease/neurodevelopmental disorder***

genetic disorder/de or exp chromosome disorder/ or exp sequence of congenital defects/ or multiple malformation syndrome/ or intellectual impairment/de or exp mental deficiency/ or "inborn error of metabolism"/ or exp X chromosome linked disorder/ or ("genetic disease*" or "genetic disorder*" or "hereditary disease*" or "hereditary disorder*" or "single-gene defect*" or "chromosome disorder*" or "chromosomal disorder*" or "inborn error metabolism*" or "inborn metabolism error*" or "intellectual disabilit*" or "mental retardation*" or "Intellectual Development Disorder*" or "Intellectual Developmental Disorder*" or "mental deficienc*" or "neurodevelopmental delay*" or "neurodevelopment delay*" or "X-linked genetic disease*").ti,ab,kw. **OR** #**(list ND/HPO for Embase)**

NOT

exp Wolff Parkinson White syndrome/ or exp Wilson disease/ or exp Gaucher disease/ or ("wolff-parkinson-white" or WPW syndrome* or "Fragile X Tremor Ataxia" or "FXTAS" or "premutation" or Wilson dis* or Wilson`s dis* or "hepatolenticular degeneration" or "Gaucher" OR GBA deficienc* or glucocerebrosidase deficienc*).ti,ab,kw.

**List of genetic disorders associated with neurodevelopmental abnormality (ND/HPO-list)**

***Adjustments to the original HPO/ND-list in order to increase the number of relevant results***

1. Removal of every “-“, e.g. “cerebro-oculo-nasal syndrome” 🡪 “cerebro oculo nasal syndrome”
2. Removal of “microdeletion/duplication” and “syndrome” from all notations concerning copy number variations (CNVs), e.g. “22q11.2 ~~microduplication~~ ~~syndrome~~”
3. Removal of “syndrome” from all notations concerning sex chromosomal abnormalities, e.g. "49,XXXYY ~~syndrome~~"
4. Removal of “syndrome” and “disease” at the end of diseases named after persons, e.g. “Prader Willi ~~syndrome~~”, in exception of e.g. “Down syndrome”, “Weaver syndrome” and “Cohen syndrome” to avoid hits with the word “down”, “weaver” (index/technique) and “Cohen” (-kappa) in another meaning.
5. Removal of “syndrome” after the term “intellectual disability”, e.g. "Spastic paraplegia glaucoma intellectual disability ~~syndrome~~"
6. Simplification of descriptive search terms:

"Partial deletion of the short arm of chromosome 7" 🡪 “7p deletion”

"Partial trisomy/tetrasomy of the short arm of chromosome 9" 🡪"trisomy 9p" OR "tetrasomy 9p"

Terms not found in the search database and replaced** by synonym (n=4) derived from Orphanet:

“Thyrocerebrorenal syndrome” 🡪 “Cutler Bass Romshe”

“Amelocerebrohypohidrotic syndrome”🡪 “Kohlschütter Tönz”

**"Autosomal recessive** cerebelloparenchymal disorder type 3" 🡪 “**Autosomal recessive spinocerebellar ataxia type 2”**

"Osteoglophonic dysplasia" 🡪"Osteoglophonic dwarfism"

***An exception for these disorders was made because of an error that resulted in the search of only the word “syndrome” or “disorder” or “dysplasia”*

# **Figure S1. Flow diagram depicting the different phases of the systematic review**

**Identification of studies via databases**

**Identification of studies via cross-reference check**

Records identified from:

Citation searching (n = 28)

Records removed before screening:

Duplicate records removed (n = 1171)

Records identified from databases (n = 5241)

PubMed 1641; Embase 3600

**Identification**

Records excluded after title/abstract screening (n = 3738)

Records screened

(n =4070)

Reports sought for retrieval (n = 28)

Reports not retrieved

(n = 2)

Reports sought for retrieval (n = 332)

**Screening**

Reports assessed for eligibility

(n = 28)

Reports excluded (n = 150)

- genetic etiology undefined
- condition not considered to be a neurodevelopmental disorder
- unclear if criteria for parkinsonism were met
- no original data
- other

Reports assessed for eligibility (n = 330)

Studies included in review (n = 208)

**Included**

Adapted from the PRISMA 2020 flow diagram.^1^

| **Figure S2. Patient characteristics and parkinsonian features per genetic disorder** | | | | | | | | | | | | | | | | | | | | | | | | |
| --- | --- | --- | --- | --- | --- | --- | --- | --- | --- | --- | --- | --- | --- | --- | --- | --- | --- | --- | --- | --- | --- | --- | --- | --- |
| 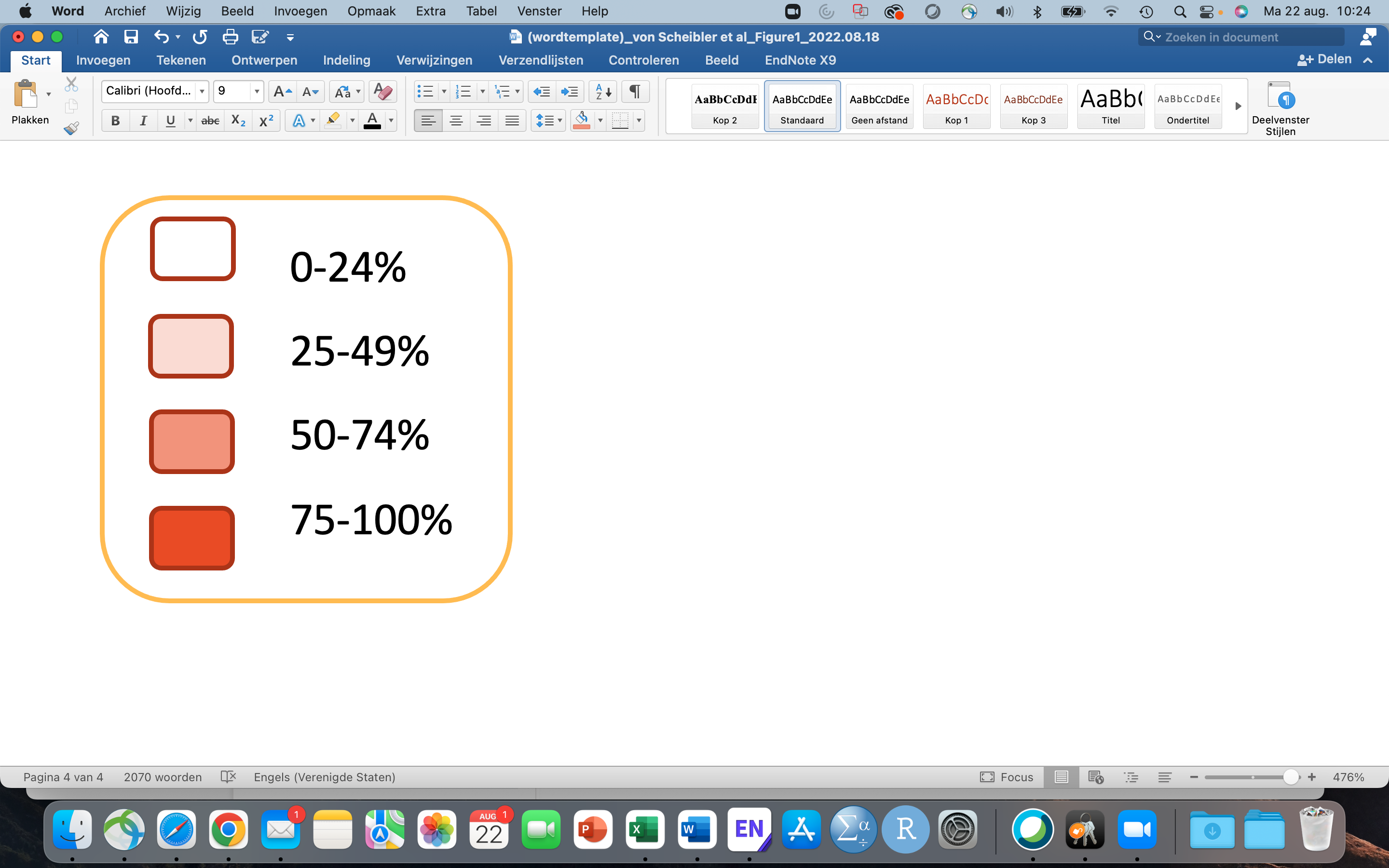  **Genetic condition** | **Number of cases** | **Male *** | **Intellectual disability *** | **Mean age at motor onset** | **Bradykinesia, hypokinesia or akinesia *** | **Rigidity *** | **Rest tremor *** | **Postural instability *** | **Asymmetric motor symptoms *** | **Progression of motor signs *** | **Reduced DAT binding with dopaminergic imaging** | **Good response anti-parkinsonian medication *** | **Levodopa-induced dyskinesia *** | **Cognitive decline/dementia** | **Psychiatric symptoms** | **Proposed main cause of parkinsonism:** | Parkinson’s disease | Genetic | Drug-induced | **Co-existing neurologic disorder:** | Epilepsy/seizures | Dystonia | Ataxia | Other |
| **Monogenic** |  |  |  |  |  |  |  |  |  |  |  |  |  |  |  |  |  |  |  |  |  |  |  |  |
| Beta-propeller protein-associated neurodegeneration (*WDR45*) | 43 | 6/  43 | 41/  41 | 25 | 40/41 | 43/43 | 8/  34 | 12/17 | 6/6 | 33/33 | 4/4 | 25/  32 | 7/  13 | 6 | 2 | 36/  42 | 0 | 35 | 1 | 40/43 | 21 | 34 | 1 | 3 |
| Cerebrotendinous xanthomatosis (*CYP27A*) | 22 | 8/  22 | 14/16 | 34 | 20/20 | 18/20 | 12/18 | 10/11 | 12/13 | 11/12 | 8/  10 | 11/  16 | 2/4 | 5 | 5 | 13/  22 | 0 | 13 | 0 | 14/22 | 1 | 1 | 10 | 6 |
| Rett syndrome (*MECP2*) | 21 | 6/  21 | 6/8 | 12 | 21/21 | 16/16 | 6/  21 | 1/2 | 1/3 | 4/4 | 0/1 | 0/3 | 0/3 | 3 | 2 | 1/21 | 0 | 1 | 0 | 8/  21 | 0 | 8 | 0 | 0 |
| *POLG* | 20 | 10/20 | 1/  20 | 51 | 19/19 | 13/15 | 13/15 | 2/2 | 11/13 | 6/6 | 8/8 | 4/5 | - | 1 | 3 | 14/  20 | 0 | 14 | 0 | 9/  20 | 0 | 1 | 8 | 0 |
| *SYNJ1* | 20 | 10/20 | 0/  18 | 26 | 20/20 | 17/19 | 16/20 | 13/13 | 19/19 | 20/20 | 3/3 | 9/17 | 9/  16 | 8 | 0 | 20/  20 | 6 | 14 | 0 | 15/20 | 7 | 11 | 0 | 3 |
| *DNAJC6* ^a^ | 19 | 8/  16 | 8/  19 | 16 | 19/19 | 19/19 | 15/19 | 19/19 | 9/11 | 19/19 | 5/5 | 11/  17 | 6/8 | 2 | 5 | 19/  19 | 5 | 14 | 0 | 11/19 | 7 | 2 | 0 | 3 |
| Tyrosine hydroxylase deficiency (*TH*) | 16 | 5/  10 | 5/9 | 4 | 11/11 | 12/13 | 10/14 | 1/1 | 0/1 | 11/13 | - | 16/  16 | 6/  14 | 0 | 1 | 12/  16 | 0 | 12 | 0 | 13/16 | 0 | 10 | 3 | 1 |
| Dravet syndrome (*SCN1A*) | 13 | 5/  12 | 13/13 | 27 | 13/13 | 12/12 | 2/  13 | 6/  13 | 9/  11 | 2/2 | 0/2 | 2/13 | 0/2 | 1 | 0 | - | - | - | - | 13/13 | 13 | 8 | 0 | 0 |
| Neurofibromatosis type I, Von Recklinghausen (*NF1*) ^b^ | 11 | 5/  11 | 1/2 | 56 | 2/2 | 2/2 | 2/3 | 1/2 | 2/2 | 2/2 | 1/1 | 1/2 | 2/2 | 0 | 9 | 9/  11 | 9 | 0 | 0 | 1/  11 | 1 | 0 | 0 | - |
| Phosphoglycerate kinase deficiency (*PGK1*) | 10 | 10/10 | 8/  10 | 22 | 10/10 | 9/  10 | 9/  10 | 6/7 | 8/8 | 10/10 | 5/5 | 3/10 | 3/5 | 0 | 3 | 4/  10 | 0 | 4 | 0 | 7/  10 | 4 | 3 | 0 | 6 |
| SPG11 | 10 | 1/4 | 5/  10 | 15 | 4/4 | 3/4 | 4/4 | 3/3 | 2/4 | 5/5 | 3/3 | 5/8 | 1/4 | 1 | 0 | 7/  10 | 0 | 7 | 0 | 2/10 | 0 | 1 | 0 | 1 |
| Neuronal ceroid lipofuscinosis, juvenile (*CLN3*) | 8 | 8/8 | 0/8 | <  19 | 8/8 | 8/8 | 0/8 | 8/8 | 0/8 | 8/8 | 8/8 | - | - | 0 | 0 | - | - | - | - | 8/8 | 8 | 0 | 0 | 0 |
| *PTRHD1* ^c^ | 7 | 4/7 | 6/7 | 24 | 6/6 | 3/7 | 4/6 | 6/6 | 5/5 | 7/7 | 1/1 | 6/6 | 2/6 | 1 | 3 | 7/7 | 0 | 7 | 0 | 5/7 | 1 | 1 | 0 | 3 |
| *RAB39B* | 7 | 7/7 | 7/7 | 38 | 7/7 | 5/5 | 6/6 | 1/1 | 3/3 | 6/6 | 3/7 | 6/7 | 3/6 | 1 | 4 | 7/7 | 1 | 6 | 0 | 5/7 | 0 | 1 | 0 | 4 |
| X-linked parkinsonism with spasticity (*ATP6AP2*) | 7 | 7/7 | 2/7 | 18 | 7/7 | 1/2 | 4/7 | 2/2 | 1/1 | - | - | - | - | 0 | 0 | 7/7 | 0 | 7 | 0 | 5/7 | 2 | 0 | 0 | 4 |
| Alexander disease (*GFAP*) | 5 | 2/5 | 1/5 | 55 | 5/5 | 5/5 | 1/2 | 0/1 | 2/2 | 1/1 | 1/2 | 1/1 | - | 2 | 0 | 3/5 | 0 | 2 | 1 | 4/5 | 0 | 0 | 3 | 1 |
| Dopa-responsive dystonia-Parkinsonism (*NR4A2*) | 5 | 3/4 | 3/5 | 41 | 5/5 | 5/5 | 4/5 | 2/2 | 5/5 | 5/5 | 3/3 | 4/4 | 1/2 | 1 | 1 | 5/5 | 2 | 3 | 0 | 3/5 | 1 | 3 | 0 | 0 |
| Fragile X syndrome (*FMR1*) ^d^ | 5 | 5/5 | 1/1 | 58 | 5/5 | 1/1 | 0/1 | 1/1 | - | 1/1 | - | - | - | 5 | 5 | 5/5 | 4 | 1 | 0 | 1/5 | 0 | 1 | 0 | - |
| SPG15 (*ZFYVE26*) | 5 | 4/5 | 3/4 | 11 | 5/5 | 4/5 | 2/5 | - | 5/5 | 5/5 | 4/4 | 3/4 | 1/2 | 2 | 0 | 3/5 | 0 | 3 | 0 | 2/5 | 0 | 0 | 0 | 2 |
| Christianson syndrome – NHE6 (*SLC9A6*) ^e^ | 4 | 2/4 | 0/3 | 44 | 4/4 | 3/3 | 1/2 | 3/4 | 3/3 | 2/2 | 1/1 | 1/1 | - | 2 | 0 | 4/4 | 0 | 4 | 0 | 2/4 | 0 | 1 | 0 | 1 |
| Dihydropteridine reductase deficiency (*QDPR*) | 4 | 3/3 | 2/2 | 13 | 2/2 | 3/3 | 3/4 | - | - | 4/4 | 0/2 | 3/3 | 2/4 | 0 | 3 | 2/2 | 0 | 2 | 0 | 1/4 | 1 | 0 | 0 | 1 |
| *DNAJC12* | 4 | 2/4 | 4/4 | 27 | 2/2 | 1/1 | 1/4 | - | - | 1/4 | 1/3 | 4/4 | 3/3 | 0 | 2 | 4/4 | 0 | 4 | 0 | 1/4 | 0 | 1 | 0 | 0 |
| Dopa-responsive dystonia (*GCH1*) | 4 | 2/4 | 0/4 | 40 | 3/3 | 2/2 | 2/2 | 1/1 | 1/2 | 2/2 | 1/1 | 3/3 | 2/3 | 1 | 0 | - | - | - | - | 2/4 | 0 | 2 | 0 | 0 |
| Dystonia 16 (*PRKRA*) | 4 | 3/4 | 0/4 | 11 | 3/3 | 1/1 | 1/1 | - | 3/3 | 3/3 | - | 0/3 | - | 0 | 0 | - | - | - | - | 4/4 | 0 | 4 | 0 | 0 |
| Glutaric aciduria type 1 | 4 | 3/4 | - | <  21 | 4/4 | 4/4 | - | - | - | - | - | - | - | 0 | 0 | - | - | - | - | 4/4 | 0 | 4 | 0 | 1 |
| Phenylketonuria (*PAH*) | 4 | 1/4 | 3/4 | 34 | 4/4 | 3/4 | 3/3 | 1/1 | 1/1 | 1/2 | 1/2 | 3/3 | 0/2 | 0 | 0 | 2/4 | 1 | 1 | 0 | 1/4 | 1 | 0 | 0 | 0 |
| *PPP2R5D* | 4 | 2/4 | 4/4 | 27 | 3/3 | 3/3 | 3/4 | 2/2 | 3/3 | 4/4 | 2/2 | 4/4 | 1/4 | 1 | 2 | 4/4 | 0 | 4 | 0 | 4/4 | 1 | 2 | 0 | 2 |
| Leigh syndrome (*MT-ATP6* and *MT-MFT*) | 3 | 1/3 | 1/3 | 31 | 2/2 | 2/2 | 2/3 | 0/1 | 0/1 | 3/3 | 1/1 | 0/2 | - | 0 | 1 | 2/3 | 1 | 1 | 0 | 3/3 | 2 | 1 | 1 | 1 |
| Menkes disease (*ATP7A*) | 3 | 0/3 | 1/1 | 50 | - | - | 1/1 | - | 3/3 | - | 1/1 | 0/2 | - | 0 | 0 | 3/3 | 0 | 3 | 0 | 1/3 | 1 | 0 | 0 | 0 |
| Dopa-responsive dystonia, Sepiapterin reductase deficiency (*SR*) | 2 | 2/2 | 1/2 | 15 | 1/1 | 1/1 | 1/2 | - | 1/1 | 2/2 | - | 2/2 | 2/2 | 0 | 0 | 1/1 | 0 | 1 | 0 | 2/2 | 1 | 2 | 0 | 1 |
| Early infantile epileptic encephalopathy 4 (*STXBP1*) | 2 | 0/2 | 2/2 | 9 | 2/2 | 2/2 | 2/2 | 1/1 | 2/2 | 2/2 | - | - | - | 0 | 0 | 2/2 | 0 | 2 | 0 | 2/2 | 2 | 1 | 1 | 2 |
| L2-hydroxyglutaric aciduria (*L2HGDH*) | 2 | 1/2 | 0/1 | 38 | 2/2 | 2/2 | 1/2 | 2/2 | 1/2 | 2/2 | 1/1 | 1/2 | 0/1 | 1 | 0 | 2/2 | 1 | 1 | 0 | 2/2 | 0 | 1 | 1 | 1 |
| SPG10 (*KIF5A*) | 2 | 0/2 | 0/2 | 55 | 2/2 | 1/1 | 1/2 | - | 2/2 | 1/1 | - | 1/1 | - | 0 | 0 | - | - | - | - | 1/2 | 0 | 0 | 0 | 1 |
| *WARS2* ^f^ | 2 | 2/2 | 0/1 | 1 | 1/1 | 0/1 | 2/2 | 1/1 | 1/1 | 2/2 | 1/1 | 2/2 | 1/1 | 0 | 0 | 1/1 | 0 | 1 | 0 | 2/2 | 0 | 2 | 0 | 1 |
| X-linked adrenoleukodystrophy (*ABCD1*) | 2 | 2/2 | 0/2 | 58 | 1/1 | 1/1 | 0/1 | - | 1/1 | 1/1 | 1/1 | 1/1 | - | 0 | 0 | 1/1 | 1 | 0 | 0 | 1/2 | 0 | 0 | 0 | 1 |
| 5,10-methylenetetrahydrofolate reductase deficiency | 1 | 0/1 | - | 2 | 1/1 | 1/1 | 1/1 | - | - | 1/1 | 1/1 | - | 0/1 | 0 | 0 | - | - | - | - | 1/1 | 0 | 0 | 1 | 0 |
| Argininosuccinate Lyase Deficiency ^g^ | 1 | 0/1 | 1/1 | 61 | 1/1 | 1/1 | 1/1 | 1/1 | 0/1 | - | - | - | - | 0 | 0 | - | - | - | - | 1/1 | 0 | 0 | 1 | 1 |
| *CLTC* ^h^ | 1 | 0/1 | 1/1 | 4 | 1/1 | 1/1 | 0/1 | - | - | 1/1 | 0/1 | 0/1 | - | 0 | 1 | 1/1 | 0 | 1 | 0 | 1/1 | 0 | 0 | 1 | 1 |
| Cornelia de Lange syndrome | 1 | 1/1 | 1/1 | 30 | 1/1 | 1/1 | 0/1 | 1/1 | 0/1 | 1/1 | - | 1/1 | 0/1 | 0 | 0 | - | - | - | - | 1/1 | 1 | 1 | 0 | 1 |
| Cowden syndrome (*PTEN*) | 1 | 0/1 | 0/1 | 66 | - | - | - | - | - | - | - | - | - | 0 | 1 | 1/1 | 1 | 0 | 0 | 0/1 | 0 | 0 | 0 | 0 |
| *MT-CYB* | 1 | 1/1 | 0/1 | 15 | 1/1 | 1/1 | 1/1 | 1/1 | 1/1 | 1/1 | - | - | - | 0 | 0 | - | - | - | - | 1/1 | 1 | 0 | 0 | 1 |
| DOORS syndrome (*ATP6V1B2*) | 1 | 1/1 | 1/1 | 63 | - | 1/1 | 1/1 | - | 1/1 | 1/1 | - | 0/1 | - | 0 | 1 | 0/1 |  |  |  | 1/1 | 1 | 0 | 0 | 1 |
| Dopamine transporter deficiency syndrome (*SLC6A3*) | 1 | - | - | 0.5 | 1/1 | 1/1 | 0/1 | 0/1 | - | 1/1 | - | - | - | 0 | 0 | 1/1 | 0 | 1 | 0 | 1/1 | 0 | 1 | 0 | 1 |
| Early-onset Lafora disease (*EPM2A*) | 1 | 0/1 | 0/1 | 14 | 1/1 | 1/1 | 1/1 | - | - | 1/1 | - | - | - | 1 | 0 | - | - | - | - | 1/1 | 1 | 0 | 0 | 1 |
| HSD10 (*HSD17B10*) | 1 | 0/1 | 1/1 | 17 | 1/1 | 1/1 | 0/1 | - | 1/1 | 1/1 | 1/1 | - | - | 1 | 1 | - | - | - | - | 0/1 | 0 | 0 | 0 | 0 |
| Incontinentia Pigmenti (*IKBKG*) | 1 | 0/1 | 0/1 | 48 | 1/1 | - | - | - | 1/1 | 1/1 | 1/1 | 1/1 | - | 1 | 0 | 1/1 | 0 | 1 | 0 | 0/1 | 0 | 0 | 0 | 0 |
| Leigh-like syndrome (*MT-TI*) | 1 | 1/1 | - | 16 | 1/1 | 1/1 | 0/1 | - | - | 1/1 | - | 1/1 | - | 1 | 1 | - | - | - | - | 0/1 | 0 | 0 | 0 | 0 |
| Mevalonic aciduria | 1 | 1/1 | 0/1 | 32 | 1/1 | 1/1 | 0/1 | - | 1/1 | 1/1 | - | - | - | - | 0 | - | - | - | - | 1/1 | 0 | 1 | 0 | 0 |
| Molybdenum cofactor deficiency type B (*MOCS2*) | 1 | 0/1 | 0/1 | 23 | 1/1 | 1/1 | 0/1 | - | 1/1 | 1/1 | - | 0/1 | - | 0 | 0 | - | - | - | - | 1/1 | 0 | 0 | 0 | 1 |
| Mucolipidosis type II (*GNPTAB*) | 1 | 1/1 | 1/1 | 16 | 1/1 | 1/1 | 1/1 | - | 1/1 | 1/1 | - | - | - | 0 | 0 | - | - | - | - | 1/1 | 0 | 0 | 0 | 1 |
| Myotonic dystrophy type 1 (*DMPK*) | 1 | 0/1 | 0/1 | 53 | 1/1 | 1/1 | 0/1 | 1/1 | 0/1 | 1/1 | 1/1 | 0/1 | - | 0 | 0 | - | - | - | - | 0/1 | 0 | 0 | 0 | 0 |
| *NUS1* | 1 | 1/1 | - | - | 1/1 | 0/1 | 1/1 | 1/1 | - | 1/1 | - | 0/1 | - | 0 | 0 | 1/1 | 0 | 1 | 0 | 1/1 | 0 | 0 | 1 | 1 |
| *PNPLA6* | 1 | 1/1 | 0/1 | 45 | 1/1 | - | 1/1 | - | - | 1/1 | 1/1 | 1/1 | - | 0 | 0 | 1/1 | 0 | 1 | 0 | 1/1 | 0 | 1 | 1 | 1 |
| Rapid onset dystonia-parkinsonism (ATP1A3) | 1 | 0/1 | 1/1 | 10 | 1/1 | 1/1 | 0/1 | - | 1/1 | 1/1 | - | - | - | 1 | 0 | 1/1 | 0 | 1 | 0 | 1/1 | 0 | 1 | 0 | 0 |
| SCA27 (*FGF14*) | 1 | 1/1 | 0/1 | 20 | 1/1 | 1/1 | 1/1 | 1/1 | 1/1 | 1/1 | - | 1/1 | - | 0 | 1 | 1/1 | 0 | 1 | 0 | 1/1 | 0 | 0 | 1 | 0 |
| Seipinopathy (BSCL2) | 1 | 1/1 | 0/1 | - | 1/1 | 1/1 | 1/1 | 0/1 | 1/1 | - | - | 1/1 | - | 0 | 0 | 1/1 | 1 | 0 | 0 | 1/1 | 0 | 0 | 0 | 1 |
| Smith-Magenis Syndrome (*RAI1*) ^e^ | (1) | 1/1 | - | 33 | 1/1 | 1/1 | 0/1 | - | - | - | - | - | - | 0 | 0 | 1/1 | 0 | 1 | 0 | 0/1 | 0 | 0 | 0 | 0 |
| Tay Sachs disease (*HEXA*) | 1 | 1/1 | 0/1 | 2 | 1/1 | 0/1 | 1/1 | 1/1 | - | 0/1 | - | - | - | 0 | 0 | - | - | - | - | 1/1 | 1 | 0 | 1 | 1 |
| **Copy number variant** |  |  |  |  |  |  |  |  |  |  |  |  |  |  |  |  |  |  |  |  |  |  |  |  |
| 22q11.2 deletion syndrome ^j^ | 56 | 35/  49 | 30/  38 | 39 | 46/47 | 46/46 | 44/47 | 21/38 | 32/40 | 20/40 | 19/20 | 32/  34 | 18/40 | 9 | 41 | 56/  56 | 48 | 8 | 0 | >17/56 | 16 | 16 | 0 | 1 |
| Angelman syndrome/ 15q11-13 deletion or uniparental disomy | 2 | 1/2 | 2/2 | 19 | 2/2 | 2/2 | 2/2 | - | - | - | - | 2/2 | - | 0 | 0 | 2/2 | 0 | 2 | 0 | 2/2 | 2 | 0 | 1 | 2 |
| 16p11.2 deletion syndrome | 1 | 1/1 | 1/1 | 17 | 1/1 | 1/1 | 0/1 | 1/1 | 1/1 | - | - | 1/1 | - | 0 | 0 | - | - | - | - | 1/1 | 0 | 0 | 1 | 1 |
| 16p11.2 duplication syndrome | 1 | 1/1 | 1/1 | 35 | 1/1 | 1/1 | 1/1 | - | 0/1 | 1/1 | 1/1 | 0/1 | 0/1 | 0 | 0 | 1/1 | 0 | 1 | 0 | 1/1 | 0 | 0 | 0 | 1 |
| 6p25 deletion | 1 | 0/1 | 1/1 | 20 | 1/1 | 1/1 | 0/1 | 1/1 | 1/1 | 1/1 | 1/1 | 1/1 | - | 0 | 0 | 1/1 | 0 | 1 | 0 | 1/1 | 0 | 0 | 0 | 1 |
| Partial 4q trisomy | 1 | 0/1 | 1/1 | 30 | 1/1 | 1/1 | 1/1 | - | 1/1 | 1/1 | 1/1 | 1/1 | 1/1 | 0 | 0 | - | - | - | - | 1/1 | 0 | 0 | 1 | 1 |
| Partial 6q trisomy ^j^ | 1 | 1/1 | 1/1 | 35 | 1/1 | 1/1 | 1/1 | 1/1 | 1/1 | 1/1 | - | 1/1 | 0/1 | 1 | 0 | 1/1 | 1 | 0 | 0 | 0/1 | 0 | 0 | 0 | 0 |
| **Aneuploid** |  |  |  |  |  |  |  |  |  |  |  |  |  |  |  |  |  |  |  |  |  |  |  |  |
| Down syndrome | 23 | 14/  18 | 6/6 | 32 | 20/22 | 21/23 | 10/23 | 1/2 | 5/5 | 6/6 | - | 9/13 | 0/5 | 18 | 2 | 18/  23 | 13 | 5 | 0 | 17/23 | 14 | 0 | 0 | 5 |
| Klinefelter syndrome ^k^ | 7 | 7/7 | 0/2 | 23 | 6/6 | 7/7 | 6/7 | 1/2 | 3/4 | 2/2 | 3/3 | 2/2 | 0/1 | 0 | 3 | 7/7 | 4 | 3 | 0 | 1/7 | 0 | 1 | 0 | 0 |
| Turner syndrome ^l^ | 2 | 0/2 | 0/1 | 50 | 1/1 | 1/1 | 0/1 | - | - | 2/2 | 1/1 | - | 1/1 | 0 | 0 | - | - | - | - | 1/2 | 0 | 0 | 0 | 1 |

* The numerator represents how many patients were reported to present with a specific feature, and the denominator represents the number of patients with data available.

Additional genetic mutations with potential relevance to parkinsonism were reported: ^a^ *LRRK2*, ^b^ *PRKN*, ^c^ *ADORA* (2 patients), ^d^ *FMR1* premutation, ^e^ One case was known to have a pathogenic variant in both *SLC9A6* and *RAI1*, ^f^ *CHRNA6*, ^g^ atypical 22q11.2 deletion of 108kb involving *PRODH* and *DGCR2*, ^h^ mild phenylalanine hydroxylase deficit with compound heterozygosity for two missense variants, ^i^ *HTRA2* (2 patients), 45,X[3]/46,XX] mosaic Turner syndrome, ^j^ *PRKN*, ^k^ *PRKN*, ^l^ *TAF1* (mosaicism): “XDP-disease-specific change 3”. - = unknown.

**sTable 1. List of included reports describing data on an individual level**

|  | **Study specifications** | | | | | | | **Patient characteristics** | | |
| --- | --- | --- | --- | --- | --- | --- | --- | --- | --- | --- |
| **Search date study number** | **First author** | **Publication year** | **Journal** | **Publication type** | **Countries of study** | **Study design** | **Number of subjects with parkinsonism** | **Age (y)** | **Sex** | **Specific genetic diagnosis** |
| **May 31, 2020** |  |  |  |  |  |  |  |  |  |  |
| 1 | Lipton^2^ | 2009 | Neurology | Letter | U.S.A. | case report | 1 | 17 | male | 16p11.2 deletion |
| 2 | Roeben^3^ | 2019 | Journal of Neurology | Letter | Germany | case report | 1 | 69 | male | 16p11.2 duplication |
| 3 | Baudoin^4^ | 2010 | Movement disord | Abstract | France | case report | 1 | 45 | male | 22q11.2 deletion |
| 4 | Booij^5^ | 2010 | Am J Med Genet Part A | Article | The Netherlands | case report | 1 | 52 | male | 22q11.2 deletion |
| 5 | Boot^6^ | 2015 | Am J Med Genet Part A | Article | Canada, The Netherlands | case series | 2 | 54 | male | 22q11.2 deletion |
| 5 |  |  |  |  |  |  |  | 38 | male | 22q11.2 deletion |
| 6 | Gambardella^7^ | 2018 | Parkinsons Dis | Article | Italy | case report | 1 | 35 | female | 22q11.2 deletion |
| 7 | Hu^8^ | 2019 | Clin Parkinsonism Relat Disord | Article | China | case report | 1 | 49 | male | 22q11.2 deletion |
| 8 | Krahn^9^ | 1998 | Mayo Clin Proc | Article | U.S.A. | case report | 1 | 30 | male | 22q11.2 deletion |
| 9 | Mills^10^ | 2016 | Ann Neurol | Abstract | U.S.A. | case report | 1 | 39 | male | 22q11.2 deletion |
| 10 | Moreira^11^ | 2018 | Movement disord | Abstract | Portugal | case report | 1 | 36 | male | 22q11.2 deletion |
| 11 | Oki^12^ | 2016 | Intern Med | Article | Japan | case report | 1 | 43 | male | 22q11.2 deletion |
| 12 | Pollard^13^ | 2016 | Parkinsonism Relat D | Article | U.S.A. | case report | 1 | 34 | female | 22q11.2 deletion |
| 13 | Rehman^14^ | 2015 | Movement disord | Article | U.S.A. | case report | 1 | 37 | male | 22q11.2 deletion |
| **Study number** | **First author** | **Publication year** | **Journal** | **Publication type** | **Countries of study** | **Study design** | **Subjects with parkinsonism** | **Age (y)** | **Sex** | **Specific genetic diagnosis** |
| 14 | Verhoeven^15^ | 2017 | Eur Arch Psychiatry Clin Neurosci | Article | The Netherlands | hidden case | 2 | 45 | male | 22q11.2 deletion |
| 14 |  |  |  |  |  |  |  | 48 | female | 22q11.2 deletion |
| 15 | Zaleski^16^ | 2009 | Am J Med Genet Part A | Article | U.S.A., Canada | case series | 2 | 46 | male | 22q11.2 deletion |
| 15 |  |  |  |  |  |  |  | 56 | male | 22q11.2 deletion |
| 16 | Foo^17^ | 2016 | Movement disord | Article | Singapore | case report | 1 | 55 | female | 22q11.2 deletion |
| 17 | Clayton^18^ | 1986 | J neurol neurosur psy | Article | U.K. | case report | 1 | 2 | female | 5,10-methylenetetrahydrofolate reductase deficiency |
| 18 | Fan^19^ | 2020 | Movement disord | Article | Taiwan | case series | 1 | 24 | female | 6p25 deletion |
| 19 | Park^20^ | 2020 | BMC Neurology | Article | Korea | case report | 1 | 58 | female | Alexander disease |
| 20 | Sechi^21^ | 2008 | Prog Neuro-Psychoph | Letter | Italy | case report | 1 | 39 | female | Alexander disease |
| 21 | Vázquez-Justes^22^ | 2020 | Clin neurol neurosur | Article | Spain | case report | 1 | 55 | male | Alexander disease |
| 22 | Yoshida^23^ | 2011 | Acta Neurol Scand | Article | Japan | case series | 2 | 73 | female | Alexander disease |
| 22 |  |  |  |  |  |  |  | 67 | male | Alexander disease |
| 23 | Harbord^24^ | 2001 | J clin neurosci | Article | Australia | case series | 2 | 23 | male | Angelman syndrome |
| 23 |  |  |  |  |  |  |  | 43 | female | Angelman syndrome |
| 24 | Woodward^25^ | 2017 | Movement disord | Abstract | U.S.A. | case report | 1 | 61 | female | Argininosuccinate Lyase Deficiency |
| 25 | Akçakaya^26^ | 2019 | Neuromol Med | Article | Turkey | case report | 1 | 34 | male | Beta-propeller protein-associated neurodegeneration |
| 26 | Hornemann^27^ | 2020 | Neuropediatrics | Article | Germany | case report | 1 | 11 | female | Beta-propeller protein-associated neurodegeneration |
| 27 | Kumada^28^ | 2015 | movement disord | Abstract | Japan | case report | 1 | 35 | female | Beta-propeller protein-associated neurodegeneration |
| 28 | Machado^29^ | 2017 | Neurology | Abstract | unknown | case report | 1 | 27 | female | Beta-propeller protein-associated neurodegeneration |
|  |  |  |  |  |  |  |  |  |  |  |
| **Study number** | **First author** | **Publication year** | **Journal** | **Publication type** | **Countries of study** | **Study design** | **Subjects with parkinsonism** | **Age (y)** | **Sex** | **Specific genetic diagnosis** |
| 29 | Ohi^30^ | 2017 | J of Neur Sci | Abstract | Japan | case report | 1 | 38 | female | Beta-propeller protein-associated neurodegeneration |
| 30 | Fonderico^31^ | 2017 | Front Neurol | Article | Italy | case report | 1 | 36 | female | Beta-propeller protein-associated neurodegeneration |
| 31 | Ichinose^32^ | 2014 | Neurol Clin Pract | Article | Japan | case report | 1 | 31 | female | Beta-propeller protein-associated neurodegeneration |
| 32 | Verhoeven^33^ | 2014 | Parkinsonism Related D | Article | The Netherlands | case series | 2 | 52 | female | Beta-propeller protein-associated neurodegeneration |
| 32 |  |  |  |  |  |  |  | 42 | female | Beta-propeller protein-associated neurodegeneration |
| 33 | Zhang^34^ | 2019 | Neurology | Abstract | U.S.A. | case report | 1 | 34 | male | Beta-propeller protein-associated neurodegeneration |
| 34 | Zitser^35^ | 2018 | Movement disorders | Article | Israel | case report | 1 | 34 | female | Beta-propeller protein-associated neurodegeneration |
| 35 | Endo^36^ | 2017 | Neurol Clin Neurosci | Article | Japan | case report | 1 | 40 | female | Beta-propeller protein-associated neurodegeneration |
| 36 | Dotti^37^ | 2000 | Movement disord | Article | Italy | case report | 1 | 52 | male | Cerebrotendinous Xanthomatosis |
| 37 | Fujiyama^38^ | 1991 | Jpn J Med | Article | Japan | case report | 1 | 44 | male | Cerebrotendinous Xanthomatosis |
| 38 | Grandas^39^ | 2002 | Movement disord | Letter | Spain | case report | 1 | 51 | female | Cerebrotendinous Xanthomatosis |
| 39 | Kuwabara^40^ | 1996 | j neurol sci | Article | Japan | case report | 1 | 34 | female | Cerebrotendinous Xanthomatosis |
| 40 | Li^41^ | 2020 | Movement disord | Letter | China | case report | 1 | 40 | male | Cerebrotendinous Xanthomatosis |
| 41 | Mignarri^42^ | 2012 | Parkinsonism Relat D | Letter | Italy | case report | 1 | 67 | female | Cerebrotendinous Xanthomatosis |
| 42 | Pilo de la Fuente^43^ | 2008 | J Neurol | Article | Spain | case report | 1 | 52 | male | Cerebrotendinous Xanthomatosis |
| 43 | Schotsmans^44^ | 2012 | Acta Neurol Belg | Article | Belgian | case report | 1 | 44 | male | Cerebrotendinous Xanthomatosis |
| 44 | Su^45^ | 2010 | Movement disord | Article | Taiwan | case report | 2 | 54 | female | Cerebrotendinous Xanthomatosis |
| 44 |  |  |  |  |  |  |  | 49 | male | Cerebrotendinous Xanthomatosis |
| **Study number** | **First author** | **Publication year** | **Journal** | **Publication type** | **Countries of study** | **Study design** | **Subjects with parkinsonism** | **Age (y)** | **Sex** | **Specific genetic diagnosis** |
| 45 | Wakamatsu^46^ | 1999 | J Neurol Neurosurg Ps | Article | Japan | case series | 3 | 46 | female | Cerebrotendinous Xanthomatosis |
| 45 |  |  |  |  |  |  |  | 43 | female | Cerebrotendinous Xanthomatosis |
| 45 |  |  |  |  |  |  |  | 33 | female | Cerebrotendinous Xanthomatosis |
| 46 | Yunisova^47^ | 2020 | Neurodegener Dis | Article | Turkey | case series | 1 | 58 | female | Cerebrotendinous Xanthomatosis |
| 47 | Zadori^48^ | 2017 | Neurol Sci | Article | Hungary | case series | 2 | 40 | female | Cerebrotendinous Xanthomatosis |
| 47 |  |  |  |  |  |  |  | 40 | female | Cerebrotendinous Xanthomatosis |
| 48 | Rubio Agusti^49^ | 2012 | Movement disorders | Abstract | U.K. | case report | 2 | 49 | male | Cerebrotendinous Xanthomatosis |
| 48 |  |  |  |  |  |  |  | 63 | male | Cerebrotendinous Xanthomatosis |
| 49 | Manti^50^ | 2019 | Parkinsonism relat d | Article | Italy | case report | 1 | 30 | female | *CLTC* |
| 50 | Fernandez^51^ | 2000 | Movement disord | Other | U.S.A. | case report | 1 | 30 | male | Cornelia de Lange syndrome |
| 51 | Rana^52^ | 2018 | J am geriatr soc | Abstract | U.S.A. | case report | 1 | 71 | female | Cowden syndrome |
| 52 | De Coo^53^ | 1999 | Ann neurol | Article | The Netherlands | case series | 1 | 20 | male | *CYTB* |
| 53 | Sedel^54^ | 2006 | Neurology | Article | France | case report | 1 | 29 | male | Dihydropteridine reductase deficiency |
| 54 | Takahashi^55^ | 2017 | Case rep neurol | Article | Japan | case report | 1 | 16 | male | Dihydropteridine reductase deficiency |
| 55 | Porta^56^ | 2012 | Mol genet metab | Article | Italy | case series | 2 | 7 | male | Dihydropteridine reductase deficiency |
| 55 |  |  |  |  |  |  |  | 22 | unknown | Dihydropteridine reductase deficiency |
| 56 | Straniero^57^ | 2017 | Ann neurol | Article | Canada | case series | 3 | 73 | male | *DNAJC12* |
| 56 |  |  |  |  |  |  |  | 59 | female | *DNAJC12* |
| 56 |  |  |  |  |  |  |  | 58 | male | *DNAJC12* |
| 57 | Anikster^58^ | 2017 | Am J Hum Genet | Article | Israel | hidden case | 1 | 13 | female | *DNAJC12* |
| **Study number** | **First author** | **Year of publication** | **Journal** | **Publication type** | **Countries of study** | **Study design** | **Subjects with parkinsonism** | **Age (y)** | **Sex** | **Specific genetic diagnosis** |
| 58 | Koroglu^59^ | 2013 | Parkinsonism relat d | Article | Turkey | case series | 4 | 24 | female | *DNAJC6* |
| 58 |  |  |  |  |  |  |  | 44 | female | *DNAJC6* |
| 58 |  |  |  |  |  |  |  | 31 | female | *DNAJC6* |
| 58 |  |  |  |  |  |  |  | 17 | male | *DNAJC6* |
| 59 | Ng^60^ | 2020 | Movement disord | Article | Pakistan | case series | 6 | 20 | female | *DNAJC6* |
| 59 |  |  |  |  |  |  |  | 12 | male | *DNAJC6* |
| 59 |  |  |  |  |  |  |  | 10 | male | *DNAJC6* |
| 59 |  |  |  |  |  |  |  | 28 | female | *DNAJC6* |
| 59 |  |  |  |  |  |  |  | 19 | female | *DNAJC6* |
| 59 |  |  |  |  |  |  |  | 18 | female | *DNAJC6* |
| 60 | Termsarasab ^61^ | 2015 | Movement disord | Abstract | U.S.A. | case report | 1 | 14 | female | *DNAJC6* |
| 61 | Edvardson^62^ | 2012 | Plos one | Article | Israel | case series | 2 | 13 | male | *DNAJC6* |
| 61 |  |  |  |  |  |  |  | 18 | male | *DNAJC6* |
| 62 | Elsayed^63^ | 2016 | Ann neurol | Letter | France | case report | 1 | 12 | female | *DNAJC6* |
| 63 | Tassin^64^ | 2000 | Brain | Article | France | case series | 3 | 76 | female | Dopa-responsive dystonia |
| 63 |  |  |  |  |  |  |  | 66 | male | Dopa-responsive dystonia |
| 63 |  |  |  |  |  |  |  | 54 | female | Dopa-responsive dystonia |
| 64 | Kikuchi^65^ | 2004 | Movement disord | Article | Japan | case report | 1 | 54 | male | Dopa-responsive dystonia autosomal dominant/ Segawa |
| 65 | Wirth^66^ | 2020 | Movement disord | Article | France | case series | 2 | 29 | male | Dopa-responsive Dystonia-Parkinsonism |
| 65 |  |  |  |  |  |  |  | 57 | female | Dopa-responsive Dystonia-Parkinsonism |
| 66 | Zielonka^67^ | 2015 | J inherit metab dis | Article | Kuwait | case report | 1 | 7 | male | Dopa-responsive dystonia, Sepiapterin reductase deficiency |
| 67 | Bodhireddy^68^ | 1994 | Neurology | Letter | U.S.A. | case report | 1 | 54 | male | Down syndrome |
| 68 | Brandel^69^ | 1994 | Neurology | Letter | France | case report | 1 | 45 | male | Down syndrome |
| 69 | Agarwal^70^ | 2010 | Movement disord | Abstract | U.S.A. | case report | 1 | 21 | female | Down syndrome |
| **Study number** | **First author** | **Publication year** | **Journal** | **Publication type** | **Countries of study** | **Study design** | **Subjects with parkinsonism** | **Age (y)** | **Sex** | **Specific genetic diagnosis** |
| 70 | Marui^71^ | 1999 | Neuropathology | Article | Japan | case report | 1 | 49 | male | Down syndrome |
| 71 | Palat^72^ | 2018 | Case Rep Neurol Med | Article | U.S.A. | case report | 1 | 20 | female | Down syndrome |
| 72 | Singer^73^ | 1990 | Eur j neurol | Article | U.S.A. | case report | 1 | 45 | male | Down syndrome |
| 73 | Storm^74^ | 1990 | Res dev disabil | Article | Germany | case report | 1 | 21 | male | Down syndrome |
| 74 | Sturman^75^ | 1989 | Lancet | Letter | U.K. | case report | 1 | 23 | male | Down syndrome |
| 75 | Camargos^76^ | 2008 | Lancet neurol | Article | Brazil | case series | 4 | 35 | male | Dystonia 16 |
| 75 |  |  |  |  |  |  |  | 34 | male | Dystonia 16 |
| 75 |  |  |  |  |  |  |  | 48 | female | Dystonia 16 |
| 75 |  |  |  |  |  |  |  | 64 | male | Dystonia 16 |
| 76 | Keogh^77^ | 2014 | Neurogenetics | Letter | U.K. | case report | 1 | 12 | female | Early infantile epileptic encephalopathy 4/ *STXBP1* |
| 77 | Rezazadeh^78^ | 2019 | Epilepsy behav | Article | Canada | hidden case | 1 | 46 | female | Early infantile epileptic encephalopathy 4/*STXBP1* |
| 78 | Yildiz^79^ | 2017 | Seizure | Article | Turkey | case report | 1 | 13 | female | Early-onset Lafora disease |
| 79 | Hall^80^ | 2010 | Movement disord | Article | U.S.A. | case report | 1 | 61 | male | Fragile X syndrome |
| 80 | Rosario^81^ | 2018 | Movement disord | Abstract | Portugal | case report | 1 | 22 | female | Hydroxysteroid dehydrogenase type 10 deficiency (HSD10) |
| 81 | Chen^82^ | 2015 | Movement disord | Abstract | U.S.A. | case report | 1 | 48 | female | Incontinentia Pigmenti |
| 82 | Bach^83^ | 2008 | Movement Disord | Letter | Germany | case report | 1 | 27 | male | Klinefelter syndrome |
| 83 | Lee^84^ | 2019 | Acta Neurol Belgica | Letter | Korea | case report | 1 | 60 | male | Klinefelter syndrome |
| 84 | Yu^85^ | 2019 | Movement disord | Abstract | China | case report | 1 |  | male | Klinefelter syndrome |
| 85 | Fabbri^86^ | 2018 | Neurol sci | Letter | Portugal | case report | 1 | 37 | male | Klinefelter syndrome |
| 86 | Owens^87^ | 2004 | J neurol neurosur ps | Letter | U.S.A. | case report | 1 | 54 | male | L2-hydroxyglutaric aciduria |
| 87 | Martikainen^88^ | 2016 | JAMA neurol | Article | U.K. | hidden case | 1 | 40 | female | Leigh syndrome/ *mt-ATP6* |
| 88 | Baumgartner^89^ | 2013 | J neurol sci | Abstract | Austria | case report | 1 | 44 | male | Leigh syndrome/ *mt-ATP6* |
| **Study number** | **First author** | **Publication year** | **Journal** | **Publication type** | **Countries of study** | **Study design** | **Subjects with parkinsonism** | **Age (y)** | **Sex** | **Specific genetic diagnosis** |
| 89 | Hemelsoet^90^ | 2018 | Neurol genet | Article | Belgium | case report | 1 | 44 | female | Leigh syndrome/*mt-FMT* |
| 90 | Martikainen^91^ | 2013 | Mitochondrion | Article | Finland | case report | 1 | 16 | male | Leigh-like syndrome |
| 91 | Zyss^92^ | 2011 | Movement disord | Abstract | France | case report | 1 | 44 | male | Mevalonic aciduria |
| 92 | Alkufri^93^ | 2013 | Movement disord | Article | United Kingdom | case report | 1 | 6 | female | Molybdenum cofactor deficiency type B |
| 93 | Hara^94^ | 2013 | Brain dev-jpn | Article | Japan | case report | 1 | 36 | male | Mucolipidosis type II |
| 94 | Choi^95^ | 2018 | Mov disord | Article | Korea | case report | 1 | 54 | female | Myotonic dystrophy type 1 |
| 95 | Pradotto^96^ | 2014 | Clin neuropathol | Abstract | Italy | case report | 1 | 65 | female | Neurofibromatosis type 1 |
| 96 | Wattanapanom^97^ | 2011 | J am geriatr soc | Abstract | U.S.A. | hidden case | 1 | 70 | male | Neurofibromatosis type 1 |
| 97 | D'Ambrosio^98^ | 1984 | Acta neurol Napoli | Article | Israel | case report | 1 | 54 | female | Neurofibromatosis type 1 |
| 98 | Hattori^99^ | 1998 | Pathol res prac | Article | Japan | case report | 1 | 58 | female | Neurofibromatosis type 1 |
| 99 | Chandra^100^ | 2018 | Movement Disord | Abstract | U.S.A. | case report | 1 | 33 | male | NHE6 - Christianson syndrome in males/ *SLC9A6* |
| 100 | Pescosolido^101^ | 2019 | Mol Neuropsychiatry | Article | U.S.A. | other | 2 | 65 | female | NHE6 - Christianson syndrome in males/ *SLC9A6* |
| 100 |  |  |  |  |  |  |  | 55 | female | NHE6 - Christianson syndrome in males/ *SLC9A6* |
| 101 | Araki^102^ | 2020 | Epilepsy res | Article | Japan | case series | 1 | 77 | male | *NUS1* |
| 102 | Namihira^103^ | 2004 | Psychiat Clin Neuros | Letter | Japan | case report | 1 | 40 | male | partial 6q trisomy |
| 103 | Garraux^104^ | 2012 | Arch neurol | Article | Belgium | case report | 1 | 31 | female | partial 4q trisomy |
| 104 | Daelman^105^ | 2014 | Rev Neurol | Article | France | case series | 1 | 47 | female | phenylketonuria |
| 105 | Evans^106^ | 2004 | Movement disord | Article | U.K. | case report | 1 | 37 | female | phenylketonuria |
| 106 | Velema^107^ | 2015 | JIMD Reports | Article | The Netherlands | case report | 1 | 56 | female | phenylketonuria |
| 107 | Leuzzi^108^ | 1995 | J Inher Metab Dis | Article | Italy | hidden case | 1 | 16 | male | phenylketonuria |
| 108 | Konrad^109^ | 1973 | J of Pediatr | Article | U.S.A. | case series | 1 | 19 | male | Phosphoglycerate kinase deficiency |
| **Study number** | **First author** | **Publication year** | **Journal** | **Publication type** | **Countries of study** | **Study design** | **Subjects with parkinsonism** | **Age (y)** | **Sex** | **Specific genetic diagnosis** |
| 109 | Sakaue^110^ | 2016 | NPJ Parkinson's dis | Article | Japan | case series | 1 | 16 | male | Phosphoglycerate kinase deficiency |
| 110 | Virmani^111^ | 2014 | Movement disord | Article | U.S.A. | case series | 2 | 30 | male | Phosphoglycerate kinase deficiency |
| 110 |  |  |  |  |  |  |  | 24 | male | Phosphoglycerate kinase deficiency |
| 111 | Morales-Briceno^112^ | 2019 | Parkinsonism relat d | Article | Australia | case series | 3 | 34 | male | Phosphoglycerate kinase deficiency |
| 111 |  |  |  |  |  |  |  | 32 | male | Phosphoglycerate kinase deficiency |
| 111 |  |  |  |  |  |  |  | 42 | male | Phosphoglycerate kinase deficiency |
| 112 | Rotstein^113^ | 2012 | Movement disord | Abstract | Israel | case report | 1 | 21 | male | Phosphoglycerate kinase deficiency |
| 113 | Sotiriou^114^ | 2010 | Muscle nerve | Article | U.S.A. | case report | 1 | 25 | male | Phosphoglycerate kinase deficiency |
| 114 | Echaniz-Laguna^115^ | 2019 | J inherit metab dis | Article | France | case report | 1 | 48 | male | Phosphoglycerate kinase deficiency |
| 87 | Martikainen^88^ | 2016 | JAMA neurol | Article | U.K. | cohort | 5 | 48 | male | *POLG* |
| 87 |  |  |  |  |  |  |  | 81 | male | *POLG* |
| 87 |  |  |  |  |  |  |  | 59 | male | *POLG* |
| 87 |  |  |  |  |  |  |  | 63 | male | *POLG* |
| 87 |  |  |  |  |  |  |  | 69 | female | *POLG* |
| 115 | Bandettini di Poggio^116^ | 2013 | BMC med genet | Article | Italy | case report | 1 | 48 | female | *POLG* |
| 116 | De Pue^117^ | 2016 | Eur j neurol | Abstract | Belgium | case report | 1 | 80 | female | *POLG* |
| 117 | Khodadadi^118^ | 2017 | Movement disord | Article | Iran | case series | 2 | 39 | male | *PTRHD1* |
| 117 |  |  |  |  |  |  |  | 37 | male | *PTRHD1* |
| 118 | Jaberi^119^ | 2016 | Movement disord | Article | Iran | case series | 2 | 34 | male | *PTRHD1* |
| 118 |  |  |  |  |  |  |  | 30 | male | *PTRHD1* |
| 119 | Kuipers^120^ | 2018 | Movement disord | Article | Netherlands | case series | 3 | 26 | female | *PTRHD1* |
| 119 |  |  |  |  |  |  |  | 29 | female | *PTRHD1* |
| 119 |  |  |  |  |  |  |  | 44 | female | *PTRHD1* |
| **Study number** | **First author** | **Publication year** | **Journal** | **Publication type** | **Countries of study** | **Study design** | **Subjects with parkinsonism** | **Age (y)** | **Sex** | **Specific genetic diagnosis** |
| 120 | Ortez^121^ | 2013 | Gene | Article | Spain | case report | 1 | 0 (5d) | female | Pyruvate carboxylase deficiency |
| 121 | Ciammola^122^ | 2017 | Parkinsonism relat d | Article |  | case series | 3 | 67 | male | *RAB39B* |
| 121 |  |  |  |  |  |  |  | 94 | male | *RAB39B* |
| 121 |  |  |  |  |  |  |  | 49 | male | *RAB39B* |
| 122 | Guldner^123^ | 2016 | Parkinsonism relat d | Letter | Germany | case series | 1 | 48 | male | *RAB39B* |
| 123 | Lesage^124^ | 2015 | Neurol genet | Article | France | case report | 1 | 39 | male | *RAB39B* |
| 124 | Wilson^125^ | 2014 | Am j human genet | Article | Australia | case series | 2 | 44 | male | *RAB39B* |
| 124 |  |  |  |  |  |  |  | 45 | male | *RAB39B* |
| 125 | Roze^126^ | 2007 | Movement disord | Other | France | case report | 1 | 49 | female | Rett syndrome |
| 126 | Chahil^127^ | 2018 | Cureus | Article | U.S.A. | case report | 1 | 16 | male | Rett syndrome/ *MECP2* |
| 127 | Venkateswaran^128^ | 2014 | Dev med child neurol | Article | Canada | case report | 1 | 15 | female | Rett syndrome/ *MECP2* |
| 128 | Pollini^129^ | 2020 | Movement disord | Letter | Italy | case report | 1 | 17 | male | Rett syndrome/ *MECP2* |
| 129 | Ollivier^130^ | 2015 | Neuromuscular Disord | Article | France | case series | 1 | 46 | male | Seipinopathy |
| 130 | Goizet^131^ | 2009 | Hum mutat | Other | France | case series | 2 | 70 | female | Spastic paraplegia type 10 |
| 130 |  |  |  |  |  |  |  | 41 | female | Spastic paraplegia type 10 |
| 131 | Anheim^132^ | 2009 | J neurol | Article | France | case series | 2 | 28 | female | Spastic paraplegia type 11 |
| 131 |  |  |  |  |  |  |  | 15 | male | Spastic paraplegia type 11 |
| 132 | Guidubaldi^133^ | 2011 | Movement disord | Article | Italy | case report | 1 | 32 | female | Spastic paraplegia type 11 |
| 133 | Kang^134^ | 2004 | Parkinsonism relat d | Article | South Korea | case series | 1 | 16 | female | Spastic paraplegia type 11 |
| 134 | Damasio^135^ | 2014 | Movement disord | Abstract | Portugal | case report | 1 | 39 | male | Spastic paraplegia type 15 |
| 135 | Mallaret^136^ | 2014 | j neurol | Letter | France | case report | 1 | 17 | female | Spastic paraplegia type 15 |
| 136 | Schicks^137^ | 2011 | Movement disord | Letter | Germany | case series | 2 | 31 | male | Spastic paraplegia type 15 |
| **Study number** | **First author** | **Publication year** | **Journal** | **Publication type** | **Countries of study** | **Study design** | **Subjects with parkinsonism** | **Age (y)** | **Sex** | **Specific genetic diagnosis** |
| 136 |  |  |  |  |  |  |  | 20 | male | Spastic paraplegia type 15 |
| 137 | Groth^138^ | 2018 | Tremor Other Hyperkinet Mov | Article | U.S.A. | case report | 1 | 70 | male | Spinocerebellar ataxia 27 |
| 138 | Ebrahimi-Fakhari^139^ | 2018 | Movement disord | Article | U.S.A. | hidden case | 1 | 4 | male | Tay Sachs disease |
| 139 | Nitschke^140^ | 2011 | Schweitz arch neurol | Abstract | Switzerland | case report | 1 | 45 | female | Turner syndrome |
| 140 | Westenberger^141^ | 2013 | Movement disord | Article | Germany | case report | 1 | 57 | female | Turner syndrome, atypical |
| 141 | De Rijk-van Andel^142^ | 2000 | Neurology | Article | The Netherlands | case series | 4 | 0 (4m) | male | Tyrosine hydroxylase deficiency |
| 141 |  |  |  |  |  |  |  | 0 (5m) | unknown | Tyrosine hydroxylase deficiency |
| 141 |  |  |  |  |  |  |  | 0 (3m) | unknown | Tyrosine hydroxylase deficiency |
| 141 |  |  |  |  |  |  |  | 0 (3m) | unknown | Tyrosine hydroxylase deficiency |
| 142 | Pons^143^ | 2010 | Movement disord | Other | Greece | case series | 3 | 2 | unknown | Tyrosine hydroxylase deficiency |
| 142 |  |  |  |  |  |  |  | 0 (5m) | unknown | Tyrosine hydroxylase deficiency |
| 142 |  |  |  |  |  |  |  | 0 (5m) | unknown | Tyrosine hydroxylase deficiency |
| 143 | Grattan-Smith^144^ | 2002 | Movement disord | Article | Australia | case report | 1 | 2 | female | Tyrosine hydroxylase deficiency |
| 144 | Haugarvoll^145^ | 2011 | J parkinson dis | Article | Norway | case report | 1 | 27 | male | Tyrosine hydroxylase deficiency |
| 145 | Ludecke^146^ | 1996 | Hum mol genet | Article | Norway | case report | 1 | 3 | female | Tyrosine hydroxylase deficiency |
| 146 | Swaans^147^ | 2000 | Ann hum genet | Article | The Netherlands, France | case series | 3 | 5 | male | Tyrosine hydroxylase deficiency |
| 146 |  |  |  |  |  |  |  | 9 | male | Tyrosine hydroxylase deficiency |
| 146 |  |  |  |  |  |  |  | 34 | male | Tyrosine hydroxylase deficiency |
| 147 | Burke^148^ | 2018 | Clin genet | Article | U.S.A. | case report | 1 | 9 | male | *WARS2* |
| **Study number and search date** | **First author** | **Publication year** | **Journal** | **Publication type** | **Countries of study** | **Study design** | **Subjects with parkinsonism** | **Age (y)** | **Sex** | **Specific genetic diagnosis** |
| 148 | Galosi^149^ | 2019 | Movement disord | Abstract | Italy | case report | 1 | 11 | male | *WARS2* |
| 149 | Horn^150^ | 2016 | Clin case rep | Article | Norway | case report | 1 | 61 | male | X-linked adrenoleukodystrophy |
| 150 | Serra Soler^151^ | 2017 | Endocrinol diabetes nutr | Letter | Spain | case report | 1 | 61 | male | X-linked adrenoleukodystrophy |
| 151 | Gupta^152^ | 2015 | Parkinsonism Relat D | Letter | U.S.A. | case series | 2 | 20 | male | X-linked parkinsonism with spasticity /*ATP6AP2* |
| 151 |  |  |  |  |  |  |  | 31 | male | X-linked parkinsonism with spasticity/ *ATP6AP2* |
| **June 15, 2021** |  |  |  |  |  |  |  |  |  |  |
| 152 | Meytin^153^ | 2020 | Movement disord | Abstract | U.S.A. | case report | 1 | 50 | male | 22q11.2 deletion |
| 153 | Buongarzone^154^ | 2020 | Movement disord | Abstract | Italy | case series | 3 | 49 | female | *ATP7A* |
| 153 |  |  |  |  |  |  |  | 54 | female | *ATP7A* |
| 153 |  |  |  |  |  |  |  | 50 | female | *ATP7A* |
| 154 | Maric^155^ | 2020 | Eur j med genet | Abstract | Spain | case report | 1 | 10 | female | Beta-propeller protein-associated neurodegeneration |
| 155 | Umehara^156^ | 2020 | Neurol clin neuroschi | Article | Japan | case report | 1 | 46 | female | Beta-propeller protein-associated neurodegeneration |
| 156 | Samanta^157^ | 2020 | J pedriatr neurosci | Article | U.S.A. | case report | 1 | 13 | female | Beta-propeller protein-associated neurodegeneration |
| 157 | Yunisova^47^ | 2020 | Neurodegener dis | Article | Turkey | case series | 1 | 58 | female | Cerebrotendinous xanthomatosis |
| 158 | Munoz-delgado^158^ | 2020 | Movement disord | Abstract | Spain | case report | 1 | 40 | male | Christianson syndrome |
| 159 | Zadori^159^ | 2020 | Front neurol | Article | Hungary | case report | 1 | 72 | male | DOORS syndrome |
| 160 | Jesus^160^ | 2021 | Neurol genet | Article | Spain | case report | 1 | 30 | male | Dopa-responsive dystonia parkinsonism |
| 161 | Sleiman^161^ | 2009 | Neurosci lett | Article | U.K. | case report | 1 | 74 | unknown | Dopa-responsive dystonia parkinsonism |
| **Study number** | **First author** | **Publication year** | **Journal** | **Publication type** | **Countries of study** | **Study design** | **Subjects with parkinsonism** | **Age (y)** | **Sex** | **Specific genetic diagnosis** |
| 162 | Grimes^162^ | 2006 | Movement dis | Article | Canada | case report | 1 | 77 | male | Dopa-responsive dystonia parkinsonism |
| 163 | Kanatani^163^ | 2021 | Brain dev-jpn | Article | Japan | case series | 2 | 20 | male | Dravet syndrome |
| 163 |  |  |  |  |  |  |  | 42 | female | Dravet syndrome |
| 164 | Malaquias^164^ | 2021 | Parkinsonism Relat D | Article | Portugal | case report | 1 | 49 | female | L2-hydroxyglutaric aciduria |
| 165 | Witt^165^ | 2020 | Movement disord | Abstract | Sweden | case report | 1 | 45 | male | *PNPLA6* |
| 166 | Kim^166^ | 2020 | Ann neurol | Article | U.S.A. | case series | 3 | 61 | male | *PPP2R5D* |
| 166 |  |  |  |  |  |  |  | 34 | male | *PPP2R5D* |
| 166 |  |  |  |  |  |  |  | 44 | female | *PPP2R5D* |
| 167 | Hetzelt^167^ | 2021 | Eur j med genet | Article | Germany | case report | 1 | 29 | female | *PPP2R5D* |
| 168 | Nomura^168^ | 2021 | Brain dev-jpn | Article | Japan | case report | 1 | 14 | female | Rapid-onset dystonia-parkinsonism |
| 169 | Araujo^169^ | 2020 | Movement disord | Article | Brazil | case report | 1 | 17 | male | Spastic paraplegia type 15 |
| 170 | Lesage^170^ | 2021 | Front neurol | Article | France | case series | 4 | 25 | male | *SYNJ1* |
| 170 |  |  |  |  |  |  |  | 31 | female | *SYNJ1* |
| 170 |  |  |  |  |  |  |  | 35 | male | *SYNJ1* |
| 170 |  |  |  |  |  |  |  | 61 | female | *SYNJ1* |
| 171 | Krebs^171^ | 2013 | Hum mutat | Article | U.S.A. | case series | 2 | 29 | male | *SYNJ1* |
| 171 |  |  |  |  |  |  |  | 39 | female | *SYNJ1* |
| 172 | Quadri^172^ | 2013 | Hum mutat | Article | Italy | case series | 2 | 47 | male | *SYNJ1* |
| 172 |  |  |  |  |  |  |  | 31 | female | *SYNJ1* |
| 173 | Olgiati^173^ | 2014 | Neurogenetics | Article | Italy | case series | 2 | 31 | male | *SYNJ1* |
| 173 |  |  |  |  |  |  |  | 27 | female | *SYNJ1* |
| 174 | Kirola^174^ | 2016 | Parkinsonism relat d | Article | India | case series | 2 | 32 | male | *SYNJ1* |
| 174 |  |  |  |  |  |  |  | 22 | female | *SYNJ1* |
| 175 | Taghavi^175^ | 2018 | Mol neurobiol | Article | Iran | case series | 2 | 30 | male | *SYNJ1* |
| 175 |  |  |  |  |  |  |  | 47 | female | *SYNJ1* |
| 176 | Ben Romdhan^176^ | 2018 | J mol neurosci | Article | Tunisia | case series | 2 | 23 | male | *SYNJ1* |
| 176 |  |  |  |  |  |  |  | 24 | female | *SYNJ1* |
| 177 | Hong^177^ | 2019 | Parkinsonism relat d | Article | China | case series | 2 | 35 | female | *SYNJ1* |
| 177 |  |  |  |  |  |  |  | 30 | male | *SYNJ1* |
| 178 | Xie^178^ | 2019 | Parkinsonism relat d | Article | China | case series | 2 | 52 | female | *SYNJ1* |
| 178 |  |  |  |  |  |  |  | 54 | male | *SYNJ1* |

Pink: identified through cross-reference check. Grey: overlap in study population with other studies but additional information provided (patients were only included once in the review). Abbreviations: U.S.A.=United States of America, U.K.=United Kingdom, y=years, m=months, d=days.

# **sTable 2. List of included reports describing data at group level**

|  | **Study specifications** | | | | | | | | **Patient characteristics** | | | |
| --- | --- | --- | --- | --- | --- | --- | --- | --- | --- | --- | --- | --- |
| **Search date and study number** | **First Author** | **Year of publication** | **Journal** | **Publication type** | **Countries of study** | **Study design** | **Study design** | **Number of subjects with parkinsonism** | **Available for subgroup with parkinsonism** | **Age (y)** | **Sex (ratio male: total)** | **Specific genetic diagnosis** |
| May 31, 2020 |  |  |  |  |  |  |  |  |  |  |  |  |
| 1 | Boot^179^ | 2018 | Neurology | Article | Canada, The Netherlands, U.K., Japan, Italy, Belgium, France, U.S.A., Germany, Chile | other | retrospective | 45:45 | yes | unknown | 32:45 | 22q11.2 deletion |
| 2 | Boot^180^ | 2020 | Movement disord | Article | Canada | Case-control/ cohort | prospective | 7:82 | partially | x=44.0 | unknown | 22q11.2 deletion |
| 3 | Butcher^181^ | 2018 | Am J Med Genet Part A | Article | Canada | case series | retrospective | 1 | yes | 50 | 1:1 | 22q11.2 deletion |
| 4 | Butcher^182^ | 2013 | JAMA Neurol | Article | Canada | cohort | retrospective | 4:68 | yes | unknown | 3:4 | 22q11.2 deletion |
| 5 | Butcher^183^ | 2017 | Brain | Article | Canada | case-control | cross-sectional | 9:14 | yes | x=43.4 ±6.5 | 5:9 | 22q11.2 deletion |
| 6 | Dufournet^184^ | 2017 | Rev Neurol | Article | France | case series | retrospective | 9:9 | yes | unknown | 8:9 | 22q11.2 deletion |
| 7 | Mok^185^ | 2016 | Lancet Neurol | Article | U.K., The Netherlands, France, Germany | case-control | other | 8:8 | partially | unknown | 6:8 | 22q11.2 deletion |
| 8 | Nishioka^186^ | 2015 | Neurobiol Aging | Article | Japan | cohort | Cross-sectional | 7:7 | yes | M=35 (33-41), x=35±3.5 | 0:7 | Beta-propeller protein-associated neurodegeneration/ *WDR45* |
| 9 | Morales-Briceño^187^ | 2018 | Movement disord | Letter | Australia, Mexico, U.K. | case series | retrospective | 3:3 | yes | M=34 (32-41), x=36.1 ± 3.8 | 1:3 | Beta-propeller protein-associated neurodegeneration/ *WDR45* |
| **Study number** | **First Author** | **Publication year** | **Journal** | **Publication type** | **Countries of study** | **Study design** | **Study design** | **Subjects with parkinsonism** | **Available for subgroup** | **Age (y)** | **Sex (ratio male: total)** | **Specific genetic diagnosis** |
| 10 | Hayflick^188^ | 2013 | Brain | Article | U.S.A., Germany, U.K., France, Italy, Canada, The Netherlands | cohort | retrospective | 19:23 | yes | M=37, x=36±8.1 | 3:19 | Beta-propeller protein-associated neurodegeneration/ *WDR45* |
| 11 | Ohno^189^ | 2001 | J Neurol Sci | Article | Japan | case series | retrospective | 3:3 | no | M=31 (31-34), x=32±1.7 | 0:3 | Cerebrotendinous xanthomatosis |
| 12 | Olgiati^190^ | 2016 | Ann neurol | Article | Italy, The Netherlands, Brazil, Portugal, Spain, and Turkey | cohort | cross-sectional | 5:274 | yes | x=51.4 | 3:5 | *DNAJC6* |
| 13 | Clot^191^ | 2009 | Brain | Article | France, Ireland, Switzerland | cohort | prospective | 4:5 | yes | x=14.3 | 1:4 | Dopa-responsive dystonia (*TH* (n=3); *SPR* (n=1)) |
| 14 | Ng^192^ | 2012 | J inherit metab dis | Abstract | U.K. | case series | retrospective | 1:5 | no | r: 2-9m | unknown | Dopamine transporter deficiency syndrome/ *SLC6A3* |
| 15 | Lai^193^ | 1989 | Arch neurol | Article | U.S.A. | cohort | prospective | 10:49 | yes | x=54 | 8:10 | Down syndrome |
| 16 | Vieregge^194^ | 1991 | J neurol neurosur ps | Article | Germany | cohort | cross-sectional | 5:14 | no | x=59.8 ±6.7 | 34:54 | Down syndrome |
| 17 | Fasano^195^ | 2014 | Neurology | Letter | Canada | cohort | prospective | 11:12 | yes | x=28.1 (r: 20-43) | 4:12 | Dravet syndrome |
| 18 | Utari^196^ | 2010 | J neurodevelop disord | Article | U.S.A. | cohort | retrospective | 4:62 | no | x=49.7 ± 8.0 | 4:4 | Fragile X syndrome |
| 19 | Gitiaux^197^ | 2008 | Movement disord | Article | France | cohort | prospective | 4:16 | yes | 21 | 3:4 | Glutaric aciduria type 1 |
| 20 | Ruottinen^198^ | 1997 | J neurol neurosur ps | Article | Finland | case-control | cross-sectional | 8:9 | no | 19.1 | 8:9 | juvenile neuronal ceroid lipofuscinosis |
| 21 | Aberg^199^ | 2000 | Neurology | Article | Finland | case-control | cross-sectional | ?:17 | no | unknown | unknown | juvenile neuronal ceroid lipofuscinosis |
| **Study number** | **First Author** | **Publication year** | **Journal** | **Publication type** | **Countries of study** | **Study design** | **Study design** | **Subjects with parkinsonism** | **Available for subgroup** | **Age (y)** | **Sex (ratio male: total)** | **Specific genetic diagnosis** |
| 22 | Aberg^200^ | 2001 | Neurology | Article | Finland | case-control | experimental | ?:21 | no | x=15 at start treatment | 9:21 | juvenile neuronal ceroid lipofuscinosis |
| 23 | Hunter^201^ | 1969 | Brit j psychiat | Article | U.K. | case-control | cross-sectional | 3:16 | no | x=37.6±13.7 (r: 17-60) | 16:16 | Klinefelter syndrome |
| 24 | Madubata^202^ | 2015 | Genet med | Article | U.S.A. | case-control | retrospective | 7:8579 | yes | x=56±5.2 | 4:7 | Neurofibromatosis type I |
| 25 | Luoma^203^ | 2004 | Lancet | Article | Finland | case series | retrospective | 13:23 | yes | x= 58.9 (r:40-75) | 6:13 | *POLG* |
| 26 | Orrico^204^ | 2000 | FEBS lett | Letter | Italy | case series | retrospective | 4:12 | yes | r:27-40 | 4:4 | Rett syndrome/ *MECP2* |
| 27 | FitzGerald^205^ | 1990 | Movement disord | Article | U.S.A. | cohort | prospective | ±13:32 | no | r:30m-28y | 0:13 | Rett syndrome/ *MECP2* |
| 28 | Kara^206^ | 2016 | Brain | Article | United Kingdom | cohort | cross-sectional | 5:30 | no | x=14.3 (r 4-27) | unknown | Spastic paraplegia type 11 |
| 29 | Zouari^207^ | 2009 | J neurol sci | Abstract | Tunisia | case series | retrospective | 1:5 | no | 27.5 | unknown | Spastic paraplegia type 11 |
| 30 | Korvatska^208^ | 2013 | Hum Mol Genet | Article | U.S.A. | case series | retrospective | 5:5 | no | r:14-58 | 5:5 | X-linked parkinsonism with spasticity / *ATP6AP2* |

^a^ No additional studies meeting inclusion criteria were found with the search on June 15th, 2021.

Pink: identified through cross-reference check. Grey: overlap in study population with other studies but additional information provided (patients were only included once in the review). Abbreviations: U.S.A.=United States of America, U.K.=United Kingdom, y=years, m=months, M=median, r=range, x=mean with standard deviation.

# **sTable 3. Studies excluded from data-extraction**

| **Search date and study number** | **First author** | **Year of publication** | **Journal** | **Reason for exclusion** |
| --- | --- | --- | --- | --- |
| **May 31,**  **2020** |  |  |  |  |
| 1 | Aggarwal | 2010 | Movement disord | parkinsonism unclear |
| 2 | Air | 2011 | J neurosurg pediat | No clear genetic diagnosis |
| 3 | Al-Thihli | 2010 | j inherit metab dis | parkinsonism unclear |
| 4 | Aljaafari | 2017 | Neurology | parkinsonism unclear |
| 5 | Alvarez | 2018 | European J of Epilepsy | parkinsonism unclear |
| 6 | Baide-Mairena | 2018 | Movement disord | No clear genetic diagnosis |
| 7 | Banka S. et al | 2011 | am j hum genet | parkinsonism unclear |
| 8 | Banuelos | 2017 | F1000res | parkinsonism unclear |
| 9 | Barnes | 2011 | ann neurol | parkinsonism unclear |
| 10 | Barnes | 2011 | ann neurol | parkinsonism unclear |
| 11 | Behnecke | 2018 | med genet | parkinsonism unclear |
| 12 | Belezhanska | 2017 | Arch Balkan Med Union | parkinsonism unclear |
| 13 | Bijarnia-Mahay | 2018 | j inherit metab dis | parkinsonism unclear |
| 14 | Blau | 2015 | mol genet metab | no new data |
| 15 | Bodzioch | 2011 | Movement disord | parkinsonism unclear |
| 16 | Bouchereau | 2018 | mol genet metab | no new data |
| 17 | Brahem | 2017 | Movement disord | Other: not clear which patient had parkinsonism (with or without GND) |
| 18 | Brahm | 2007 | Clinical Schizophrenia and Related Psychoses | No clear genetic diagnosis |
| 19 | Brajkovic | 2010 | Eur J Nucl Med Mol I | No clear genetic diagnosis |
| 20 | Brajkovic | 2012 | Hell J nucl med | No clear genetic diagnosis |
| 21 | Brautigam | 1999 | cin chem | parkinsonism unclear |
| 22 | Buckley | 2017 | NeuroReport | parkinsonism unclear |
| 23 | Burt | 1980 | Eur Neurol | Other: unclear of the patient with Down syndrome had parkinsonism, also for other GNDs outcome measures are on group level |
| 24 | Butcher | 2016 | Biol Psychiat | no new data |
| 25 | Butcher | 2014 | Mov Disord | no new data |
| 26 | Butcher | 2015 | Mov Disord | no new data |
| 27 | Butcher | 2017 | PLOS ONE | no new data |
| 28 | Byrne | 2015 | neuromuscular disord | parkinsonism unclear |
| **Search date and study number** | **First author** | **Year of publication** | **Journal** | **Reason for exclusion** |
| 29 | Carecchio | 2017 | Eur J Neurol | Other: Information on the whole group is provided, including genetic disorders not meeting our definition. |
| 30 | Carecchio | 2011 | movement disord | No clear genetic diagnosis |
| 31 | Castello | 2012 | clin neuropathol | parkinsonism unclear |
| 32 | Chitty | 2016 | aust nz j psychiat | No clear genetic diagnosis |
| 33 | Cubells | 2010 | Neuropsychopharmacol | not relevant |
| 34 | Cukiert | 2009 | epilepsia | No clear genetic diagnosis |
| 35 | de Kuijper | 2013 | res dev disabil | No clear genetic diagnosis |
| 36 | De Lonlay | 2000 | j inherit metab dis | parkinsonism unclear |
| 37 | Demirbas | 2018 | mol genet metab | parkinsonism unclear |
| 38 | Deuel | 2019 | Neurology | parkinsonism unclear |
| 39 | Dhivya | 2016 | Int J Hum Genet | Other: Aim, methods, and results all unclear |
| 40 | Dobyns | 1993 | Neurology | No clear genetic diagnosis |
| 41 | Dufournet | 2015 | Eur J Neurol | no new data |
| 42 | Dulovic | 2016 | movement disord | parkinsonism unclear |
| 43 | Ekinci | 2004 | movement disord | No clear genetic diagnosis |
| 44 | Elahi | 2017 | movement disord | no new data |
| 45 | Fan | 2020 | movement disord | parkinsonism unclear |
| 46 | Fanella | 2019 | J Med Genet | parkinsonism unclear |
| 47 | Finsterer | 2011 | acta neurol belg | Other: heterozygous for autosomal recessive disorder; usually asymptomatic |
| 48 | Fitzgerald | 1990 | neurology | no new data |
| 49 | Fraser | 2019 | Ann Neurol | No clear genetic diagnosis |
| 50 | Galati | 2015 | j neuropsych clin n | No clear genetic diagnosis |
| 51 | Gao | 2020 | movement disord | no new data |
| 52 | Gascon | 1994 | brain & development | parkinsonism unclear |
| 53 | Grant | 1992 | eur j pediatr | No clear genetic diagnosis |
| 54 | Gunzler | 2007 | movement disord | No clear genetic diagnosis |
| 55 | Haack | 2012 | Am J Hum Genet | Other: No patient data (apart from genetic data) is available. |
| 56 | Habermeyer | 2009 | j neuropsych clin n | No clear genetic diagnosis |
| 57 | Hama | 2017 | j neurol sci | No clear genetic diagnosis |
| 58 | Hermann | 2017 | Tremor Other Hyperkinet Mov | parkinsonism unclear |
| 59 | Hernandez Navarro | 2017 | eur j neurol | parkinsonism unclear |
| 60 | Hestnes | 1997 | j neurol neurosur psy | parkinsonism unclear |
| 61 | Hjalgrim | 2011 | epilepsia | parkinsonism unclear |
| 62 | Humphreys | 2016 | can j neurol sci | parkinsonism unclear |
| 63 | Humphreys | 2010 | can j neurol sci | no new data |
| **Search date and study number** | **First author** | **Year of publication** | **Journal** | **Reason for exclusion** |
| 64 | Illsinger | 2011 | eur j paediatr neuro | parkinsonism unclear |
| 65 | Jacobsen | 1998 | j med genet | parkinsonism unclear |
| 66 | Kaleka | 2019 | Cureus | parkinsonism unclear |
| 67 | Kalsner | 2013 | ann neurol | No clear genetic diagnosis |
| 68 | Kara | 2013 | Movement disord | parkinsonism unclear |
| 69 | Kim | 2018 | Int J Neurosci | No clear genetic diagnosis |
| 70 | Klysz | 2014 | neurol neurochir pol | parkinsonism unclear |
| 71 | Konrad | 1973 | J of Pediatr | parkinsonism unclear |
| 72 | Kruer | 2009 | dev med child neurol | No clear genetic diagnosis |
| 73 | Kuiper | 2014 | movement disord | parkinsonism unclear |
| 74 | Kuipers | 2019 | parkinsonism relat d | No/unclear Neurodevelopmental disorder |
| 75 | Larnaout | 2008 | j inherit metab dis | parkinsonism unclear |
| 76 | Laxova | 1985 | am j med genet | No clear genetic diagnosis |
| 77 | Lee | 2012 | movement disord | No/unclear Neurodevelopmental disorder |
| 78 | Leuzzi | 2010 | Clin Genet | parkinsonism unclear |
| 79 | Lindsay | 1996 | Am J Hum Genet | No clear genetic diagnosis |
| 80 | Lohmann | 2019 | eur j hum genet | Other: unclear which participants with genetic variants had parkinsonism |
| 81 | Lorea | 2015 | j inherit metab dis | parkinsonism unclear |
| 82 | Masingue | 2017 | eur j neurol | parkinsonism unclear |
| 83 | Matsuura | 2019 | Movement disord | Other: no GND (specifically mentioned) with parkinsonism. There may be patients in the group "other". |
| 84 | Melberg | 1996 | muscle nerve | No clear genetic diagnosis |
| 85 | Mellick | 2004 | movement disord | No clear genetic diagnosis |
| 86 | Morales | 2017 | Movement disord | No new data |
| 87 | Morton | 1997 | dev med child neurol | parkinsonism unclear |
| 88 | Neri | 2012 | neuromuscular disord | No clear genetic diagnosis |
| 89 | Nissenkorn | 2012 | J Child Neurol | parkinsonism unclear |
| 90 | Olszewska | 2018 | movement disord | no new data |
| 91 | Ortez | 2015 | mol genet metab | parkinsonism unclear |
| 92 | Palumbo | 2016 | Mol Syndromol | parkinsonism unclear |
| 93 | Panteghini | 2016 | Movement disord | parkinsonism unclear |
| 94 | Papendreou | 2017 | dev med child neurol | Other: unclear which participant/genetic disorder had parkinsonism |
| 95 | Park | 2014 | Movement disord | parkinsonism unclear |
| 96 | Peer Zada | 2015 | movement disord | No new data |
| 97 | Pilotto | 2017 | Eur J Neurol | parkinsonism unclear |
| **Search date and study number** | **First author** | **Year of publication** | **Journal** | **Reason for exclusion** |
| 98 | Pilotto | 2017 | J Inborn Errors Metab Screen | parkinsonism unclear |
| 99 | Pilotto | 2019 | Eur J Neurol | parkinsonism unclear |
| 100 | Porta | 2010 | mol genet metab | no new data |
| 101 | Porta | 2009 | Neurology | parkinsonism unclear |
| 102 | Raghavan | 1993 | can j neurol sci | parkinsonism unclear |
| 103 | Ramerman | 2018 | rev des disabil | No clear genetic diagnosis |
| 104 | Riahi | 2016 | Eur J Neurol | No clear genetic diagnosis |
| 105 | Rodan | 2018 | mol genet metab | parkinsonism unclear |
| 106 | Rosini | 2014 | J Neurol | parkinsonism unclear |
| 107 | Rubio-Agusti | 2011 | movement disord | parkinsonism unclear |
| 108 | Russo | 2019 | Movement disord | parkinsonism unclear |
| 109 | Sarpong | 2009 | clin genet | parkinsonism unclear |
| 110 | Scheifes | 2016 | j clin pharmacol | No clear genetic diagnosis |
| 111 | Schicks | 2011 | movement disord | parkinsonism unclear |
| 112 | Schicks | 2010 | movement disord | no new data |
| 113 | Sheehan | 2017 | bmj brit med j | No clear genetic diagnosis |
| 114 | shi | 2016 | movement disorders | parkinsonism unclear |
| 115 | Singh | 1986 | brit j psychiat | parkinsonism unclear |
| 116 | Soto | 2017 | J Inborn Errors Metab Screen | parkinsonism unclear |
| 117 | Stagnaro | 2018 | eur j paediatr neuro | parkinsonism unclear |
| 118 | Stelten | 2018 | Neurology | parkinsonism unclear |
| 119 | Straniero | 2019 | eur j hum genet | no new data |
| 120 | Surtees | 1998 | movement disord | no new data |
| 121 | Tan | 2016 | Lancet Neurol | Not relevant |
| 122 | Tanaka | 1989 | Eur J Pediatr | parkinsonism unclear |
| 123 | Temudo | 2008 | movement disord | parkinsonism unclear |
| 124 | Troncoso | 2015 | j neurol sci | No clear genetic diagnosis |
| 125 | Tufekcioglu | 2016 | Neurocase | parkinsonism unclear |
| 126 | Valadares | 2011 | arq neuro-psiquiat | parkinsonism unclear |
| 127 | Vellingiri | 2018 | Parkinsonism Relat Disord | Other: Aim, methods, and results all unclear |
| 128 | Verhoeven | 2015 | Eur Arch Psychiatry Clin Neurosci | no new data |
| 129 | Wang | 2017 | Stem cel res | not relevant |
| 130 | Waschbisch | 2010 | j neurol sci | parkinsonism unclear |
| 131 | Weinshenker | 2008 | nature | not relevant |
| 132 | Wille | 2018 | neuropediatrics | parkinsonism unclear |
| **Search date and study number** | **First author** | **Year of publication** | **Journal** | **Reason for exclusion** |
| 133 | Wise | 2012 | dev med child neurol | no new data |
| 134 | Yemni | 2019 | nature | No/unclear Neurodevelopmental disorder |
| 135 | Yoon | 2008 | J Neurol Neurosurg Ps | parkinsonism unclear |
| 136 | Zouari | 2018 | movement disord | Other: unclear which participants had a GND with parkinsonism |
| 137 | Zweije-Hofman | 1982 | clin neurol neurosur | parkinsonism unclear |
| **June 15,**  **2021** |  |  |  |  |
| 138 | Agabna | 2021 | dev med child neurol | parkinsonism unclear |
| 139 | De Jesus | 2020 | movement disord | parkinsonism unclear |
| 140 | Di lazarro | 2020 | parkinsonism relat d | Parkinsonism unclear |
| 141 | Eis | 2020 | front neurol | No/unclear Neurodevelopmental disorder |
| 142 | Jan | 2021 | nature | Parkinsonism unclear |
| 143 | Kisa | 2021 | metab brain dis | Parkinsonism unclear |
| 144 | Krbanjevic | 2021 | am j dermatopathol | parkinsonism unclear |
| 145 | Manti | 2020 | movement disord | parkinsonism unclear |
| 146 | Munoz | 2020 | movement disord | Other: unclear which symptoms were present in which patients |
| 147 | Nardecchia | 2020 | movement disord | Parkinsonism unclear |
| 148 | Shabeer | 2021 | J Neurosci Rural Pract | Parkinsonism unclear |
| 149 | Skrahina | 2020 | movement disord | Other: no specific GND was mentioned |
| 150 | Yuan | 2002 | neurodegener dis | parkinsonism unclear |

Abbreviations: GND= genetic neurodevelopmental disorder.

# **sTable 4. Quality assessment of observational cohort and cross-sectional studies**

| First author | Year of publication | Journal | Clear research question | Clear subject population | At least 50% participation rate | Subjects recruited from the same population.  Prespecified in/ exclusion criteria | Sample size justification, power description, variance/effect estimate | Exposure(s) of interest measured prior to the outcome(s) being measured | Sufficient timeframe between exposure and outcome | Different levels of the exposure examined as related to the outcome | Exposure measures clearly defined, valid, reliable, and implemented consistently | Exposure assessed more than once over time | Outcome measures clearly defined, valid, reliable, and implemented consistently | Outcome assessors blinded to the exposure status | Loss to follow-up 20% or less | Potential confounding variables measured and adjusted statistically | Rating |
| --- | --- | --- | --- | --- | --- | --- | --- | --- | --- | --- | --- | --- | --- | --- | --- | --- | --- |
| Boot | 2018 | Neurology | + | + | na | + | na | na | na | na | na | na | + | na | na | na | G |
| Butcher | 2013 | JAMA Neurol | + | + | + | + | + | na | na | na | na | na | + | na | na | na | G |
| Clot | 2009 | Brain | + | + | cd | + | na | na | na | na | na | na | + | na | cd | na | G |
| Fasano | 2014 | Neurology | + | cd | cd | + | na | na | na | na | na | na | + | na | nr | na | F |
| Fitzgerald | 1990 | Movement disord | + | + | + | + | na | na | na | na | na | na | + | na | nr | na | G |
| Gitiaux | 2008 | Movement disord | + | + | cd | + | na | na | na | na | na | na | - | na | nr | na | F |
| Hayflick | 2013 | Brain | + | + | na | + | na | na | na | na | na | na | + | na | na | na | G |
| Kara | 2016 | Brain | + | + | cd | + | na | na | na | na | na | na | + | na | na | na | F |
| Lai | 1989 | Arch neurol | + | + | + | + | na | na | na | na | na | na | + | na | nr | na | G |
| Martikainen | 2016 | JAMA neurol | + | + | + | + | na | na | na | na | na | na | + | na | na | na | G |
| Nishioka | 2015 | Neurobiol Aging | + | + | na | + | na | na | na | na | na | na | + | na | na | na | G |
| Olgiati | 2016 | Ann neurol | + | + | cd | + | na | na | na | na | na | na | + | na | na | na | G |
| Utari | 2010 | J neurodevelop disord | + | + | cd | + | na | na | na | na | na | na | + | na | na | na | G |
| Vieregge | 1991 | J neurol neurosur ps | + | + | cd | cd | na | na | na | na | na | na | + | na | na | na | F |

Abbreviations: yes (+) / no (-) / cannot determine (cd) /not applicable (na) / not reported (nr). G=Good, F=Fair.
Source assessment form: https://www.nhlbi.nih.gov/health-topics/study-quality-assessment-tools.

# **sTable 5. Quality assessment of case-control studies**

| First author | Year of publication | Journal | Clear research question | Clear study population | Sample size justification included | Subjects recruited from same population | Definitions, in/exclusion criteria were valid, reliable, and implemented consistently | Clearly defined cases and controls | Cases and/or controls randomly selected from those eligible | Use of concurrent controls | exposure/risk occurred prior to the development of the condition that defined a participant as a case | Measures of exposure clearly defined, valid, reliable, and implemented consistently | Assessors of exposure were blinded to the case or control status | Confounding variables measured and adjusted statistically or: account for matching during analysis | Rating |
| --- | --- | --- | --- | --- | --- | --- | --- | --- | --- | --- | --- | --- | --- | --- | --- |
| Aberg | 2000 | Neurology | + | + | - | - | + | + | na | na | na | na | na | - | F |
| Aberg | 2001 | Neurology | + | + | - | + | + | + | na | + | na | na | na | - | G |
| Boot | 2020 | Movement Disord | + | + | - | + | + | + | na | na | na | na | na | + | G |
| Butcher | 2017 | Brain | + | + | - | + | + | + | na | na | na | na | na | + | G |
| Hunter | 1969 | Brit j psychiat | - | + | - | + | - | + | na | cd | na | na | na | - | P^a^ |
| Madubata | 2014 | Genet med | + | + | - | + | + | + | na | + | na | na | na | + | G |
| Mok | 2016 | Lancet Neurol | + | + | - | + | + | + | na | na | na | na | na | + | G |
| Ruottinen | 1997 | J neurol neurosur ps | + | + | - | - | + | + | na | na | na | na | na | - | F |

^a^ Considered poor because of a very limited description of aims and methods. However, data regarding parkinsonism in Klinefelter syndrome were clear enough to be used for data-extraction. Abbreviations: yes (+) / no (-) / cannot determine (cd) /not applicable (na) / not reported (nr). G=Good, F=Fair, P=Poor.

Source assessment form: https://www.nhlbi.nih.gov/health-topics/study-quality-assessment-tools.

# **sReferences**

1. Page MJ, McKenzie JE, Bossuyt PM, et al. The PRISMA 2020 statement: an updated guideline for reporting systematic reviews. *BMJ*. 2021;372:n71. doi:10.1136/bmj.n71

2. Lipton J, Rivkin MJ. 16p11.2-related paroxysmal kinesigenic dyskinesia and dopa-responsive parkinsonism in a child. *Neurology*. 2009;73(6):479-480. doi:http://dx.doi.org/10.1212/WNL.0b013e3181b16393

3. Roeben B, Blum D, Gabriel H, Synofzik M. Atypical parkinsonism with severely reduced striatal dopamine uptake associated with a 16p11.2 duplication syndrome. *J Neurol*. Mar 2019;266(3):775-776. doi:10.1007/s00415-019-09182-7

4. Baudoin H, Jacquette A, Grabli D, Whalen S, Lenglet T. The co-occurence of early onset Parkinson's disease (PD) and 22q11.2 deletion syndrome (22qDS): More than a coincidence. *Movement Disorders*. 2010;25(SUPPL. 2):S468-S469. 14th International Congress of Parkinson's Disease and Movement Disorders. Buenos Aires Argentina.

(var.pagings). doi:http://dx.doi.org/10.1002/mds.23162

5. Booij J, van Amelsvoort T, Boot E. Co-occurrence of early-onset Parkinson disease and 22q11.2 deletion syndrome: Potential role for dopamine transporter imaging. *Am J Med Genet A*. Nov 2010;152a(11):2937-8. doi:10.1002/ajmg.a.33665

6. Boot E, Butcher NJ, van Amelsvoort TA, et al. Movement disorders and other motor abnormalities in adults with 22q11.2 deletion syndrome. *Am J Med Genet A*. Mar 2015;167a(3):639-45. doi:10.1002/ajmg.a.36928

7. Gambardella S, Ferese R, Scala S, et al. Mitochondrial Serine Protease HTRA2 p.G399S in a Female with Di George Syndrome and Parkinson's Disease. *Parkinsons Dis*. 2018;2018:5651435. doi:10.1155/2018/5651435

8. Hu ZX, Lu XD, Lou DN, et al. A case report of a Chinese patient with 22q11.2 deletion accompanied with EOPD, severe dystonia and hypocalcemia. *Clinical Parkinsonism and Related Disorders*. 2019;1:72-73. doi:http://dx.doi.org/10.1016/j.prdoa.2019.07.002

9. Krahn LE, Maraganore DM, Michels VV. Childhood-onset schizophrenia associated with parkinsonism in a patient with a microdeletion of chromosome 22. *Mayo Clinic Proceedings*. 1998;73(10):956-959. doi:http://dx.doi.org/10.4065/73.10.956

10. Mills KA, Reich SG. Early onset parkinson's disease and chromosome 22q11 deletion: Case report. *Annals of Neurology*. 2016;80(Supplement 20):S94. 141st Annual Meeting of the American Neurological Association, ANA 2016. United States. doi:http://dx.doi.org/10.1002/ana.24759

11. Moreira F, Bras A, Lopes JR, Januario C. Parkinson's disease with hypocalcaemia: adult presentation of 22q11.2 deletion syndrome. *BMJ Case Rep*. Mar 22 2018;2018doi:10.1136/bcr-2017-223751

12. Oki M, Hori S, Asayama S, Wate R, Kaneko S, Kusaka H. Early-onset Parkinson's Disease Associated with Chromosome 22q11.2 Deletion Syndrome. *Intern Med*. 2016;55(3):303-5. doi:10.2169/internalmedicine.55.5485

13. Pollard R, Hannan M, Tanabe J, Berman BD. Early-onset Parkinson disease leading to diagnosis of 22q11.2 deletion syndrome. *Parkinsonism Relat Disord*. Apr 2016;25:110-1. doi:10.1016/j.parkreldis.2016.01.027

14. Rehman AF, Dhamija R, Williams ES, Barrett MJ. 22q11.2 deletion syndrome presenting with early-onset Parkinson's disease. *Mov Disord*. Aug 2015;30(9):1289-90. doi:10.1002/mds.26305

15. Verhoeven WMA, Egger JIM, De Leeuw N. Psychopathology and differentiated psychopharmacological treatment strategy in subtypes of 22q11.2 deletion syndrome. *European Archives of Psychiatry and Clinical Neuroscience*. 2017;267(1 Supplement 1):S64-S65. 6th European Conference on Schizophrenia Research: Advancing Research - Promoting Recovery. Germany. doi:http://dx.doi.org/10.1007/s00406-017-0824-8

16. Zaleski C, Bassett AS, Tam K, Shugar AL, Chow EW, McPherson E. The co-occurrence of early onset Parkinson disease and 22q11.2 deletion syndrome. *Am J Med Genet A*. Mar 2009;149a(3):525-8. doi:10.1002/ajmg.a.32650

17. Foo JN, Lee J, Tan LC, Liu J, Tan EK. Large 3-Mb deletions at 22q11.2 locus in Parkinson's disease and schizophrenia. *Mov Disord*. Dec 2016;31(12):1924-1925. doi:10.1002/mds.26822

18. Clayton PT, Smith I, Harding B. Subacute combined degeneration of the cord, dementia and Parkinsonism due to an inborn error of folate metabolism. *Journal of Neurology Neurosurgery and Psychiatry*. 1986;49(8):920-927.

19. Fan SP, Lee NC, Lin CH. Novel Phenotype of 6p25 Deletion Syndrome Presenting Juvenile Parkinsonism and Brain Calcification. *Mov Disord*. May 5 2020;doi:10.1002/mds.28079

20. Park J, Park ST, Kim J, Kwon KY. A case report of adult-onset Alexander disease clinically presenting as Parkinson's disease: is the comorbidity associated with genetic susceptibility? *BMC Neurol*. Jan 17 2020;20(1):27. doi:10.1186/s12883-020-1616-8

21. Sechi GP, Conti M, Sau GF, Cocco GA. Valproate-induced parkinsonism, glial cells and Alexander's disease. *Prog Neuropsychopharmacol Biol Psychiatry*. Jul 1 2008;32(5):1351-2. doi:10.1016/j.pnpbp.2008.03.022

22. Vazquez-Justes D, Penalva-Garcia J, Lopez R, Mitjana R, Begue R, Gonzalez-Mingot C. Parkinsonism phenotype in a family with adult onset Alexander disease and a novel mutation of GFAP. *Clin Neurol Neurosurg*. May 6 2020;195:105893. doi:10.1016/j.clineuro.2020.105893

23. Yoshida T, Sasayama H, Mizuta I, et al. Glial fibrillary acidic protein mutations in adult-onset Alexander disease: clinical features observed in 12 Japanese patients. *Acta Neurol Scand*. Aug 2011;124(2):104-8. doi:10.1111/j.1600-0404.2010.01427.x

24. Harbord M. Levodopa responsive Parkinsonism in adults with Angelman Syndrome. *J Clin Neurosci*. Sep 2001;8(5):421-2. doi:10.1054/jocn.2000.0753

25. Woodward K, Bhatti D, Rush E. First reported case of parkinsonism in a patient with argininosuccinate lyase deficiency. *Movement Disorders*. 2017;32(Supplement 2):703-704. 21st International Congress of Parkinson's Disease and Movement Disorders. Canada. doi:http://dx.doi.org/10.1002/mds.27087

26. Akcakaya NH, Salman B, Gormez Z, et al. A Novel and Mosaic WDR45 Nonsense Variant Causes Beta-Propeller Protein-Associated Neurodegeneration Identified Through Whole Exome Sequencing and X chromosome Heterozygosity Analysis. *Neuromolecular Med*. Mar 2019;21(1):54-59. doi:10.1007/s12017-018-08522-6

27. Hornemann F, Le Duc D, Roth C, Pfaffle R, Huhle D, Merkenschlager A. Childhood Dystonia-Parkinsonism Following Infantile Spasms-Clinical Clue to Diagnosis in Early Beta-Propeller Protein-Associated Neurodegeneration. *Neuropediatrics*. Feb 2020;51(1):22-29. doi:10.1055/s-0039-1696688

28. Kumada S, Hayakawa I, Nakata Y, et al. A functional neuroradiological study in an adult case of betapropeller protein-associated neurodegeneration. *Movement Disorders*. 2015;30(SUPPL. 1):S384-S385. 19th International Congress of Parkinson's Disease and Movement Disorders. San Diego, CA United States.

(var.pagings). doi:http://dx.doi.org/10.1002/mds.26295

29. Machado D. Genetic testing for beta-propeller protein-associated neurodegeneration (BPAN). *Neurology*. 2017;88(16 Supplement 1)69th American Academy of Neurology Annual Meeting, AAN 2017. United States.

30. Ohi K, Kageyama Y, Ichikawa K, Nishioka K. Clinical investigation of beta-propeller protein associated neurogeneration (BPAN). *Journal of the Neurological Sciences*. 2017;381(Supplement 1):701-702. 23rd World Congress of Neurology, WCN 2017. Japan. doi:http://dx.doi.org/10.1016/j.jns.2017.08.1975

31. Fonderico M, Laudisi M, Andreasi NG, et al. Patient Affected by Beta-Propeller Protein-Associated Neurodegeneration: A Therapeutic Attempt with Iron Chelation Therapy. *Front Neurol*. 2017;8:385. doi:10.3389/fneur.2017.00385

32. Ichinose Y, Miwa M, Onohara A, et al. Characteristic MRI findings in beta-propeller protein-associated neurodegeneration (BPAN). *Neurol Clin Pract*. Apr 2014;4(2):175-177. doi:10.1212/01.CPJ.0000437694.17888.9b

33. Verhoeven WM, Egger JI, Koolen DA, et al. Beta-propeller protein-associated neurodegeneration (BPAN), a rare form of NBIA: novel mutations and neuropsychiatric phenotype in three adult patients. *Parkinsonism Relat Disord*. Mar 2014;20(3):332-6. doi:10.1016/j.parkreldis.2013.11.019

34. Zhang L, Malhado-Chang N, Shankar S, Hagerman R. Autistic parkinsonism: NBIA with mosaic WDR45 mutation and datscan positive dopa-responsiveness. *Neurology*. 2019;92(15 Supplement 1)71st Annual Meeting of the American Academy of Neurology, AAN 2019. United States.

35. Zitser J, Giladi N, Gurevich T. A Case with Beta-Propeller Protein Associated Neurodegeneration with Smooth Response to Levodopa Treatment. *Movement Disorders Clinical Practice*. 2018;5(3):327-329. doi:http://dx.doi.org/10.1002/mdc3.12604

36. Endo H, Uenaka T, Satake W, et al. Japanese WDR45 de novo mutation diagnosed by exome analysis: A case report. *Neurol Clin Neurosci*. Jul 2017;5(4):131-133. doi:10.1111/ncn3.12132

37. Dotti MT, Federico A, Garuti R, Calandra S. Cerebrotendinous xanthomatosis with predominant parkinsonian syndrome: further confirmation of the clinical heterogeneity. *Mov Disord*. Sep 2000;15(5):1017-9. doi:10.1002/1531-8257(200009)15:5<1017::aid-mds1043>3.0.co;2-f

38. Fujiyama J, Kuriyama M, Yoshidome H, et al. Parkinsonism in cerebrotendinous xanthomatosis. *Jpn J Med*. Mar-Apr 1991;30(2):189-92. doi:10.2169/internalmedicine1962.30.189

39. Grandas F, Martin-Moro M, Garcia-Munozguren S, Anaya F. Early-onset parkinsonism in cerebrotendinous xanthomatosis. *Mov Disord*. Nov 2002;17(6):1396-7. doi:10.1002/mds.10287

40. Kuwabara K, Hitoshi S, Nukina N, et al. PET analysis of a case of cerebrotendinous xanthomatosis presenting hemiparkinsonism. *J Neurol Sci*. Jun 1996;138(1-2):145-9. doi:10.1016/0022-510x(96)00011-1

41. Li J, Xu EH, Mao W, et al. Parkinsonism with Normal Dopaminergic Presynaptic Terminals in Cerebrotendinous Xanthomatosis. *Mov Disord Clin Pract*. Jan 2020;7(1):115-116. doi:10.1002/mdc3.12846

42. Mignarri A, Falcini M, Vella A, et al. Parkinsonism as neurological presentation of late-onset cerebrotendinous xanthomatosis. *Parkinsonism Relat Disord*. Jan 2012;18(1):99-101. doi:10.1016/j.parkreldis.2011.06.004

43. Pilo de la Fuente B, Ruiz I, Lopez de Munain A, Jimenez-Escrig A. Cerebrotendinous xanthomatosis: neuropathological findings. *J Neurol*. Jun 2008;255(6):839-42. doi:10.1007/s00415-008-0729-6

44. Schotsmans K, De Cauwer H, Baets J, et al. Cerebrotendinous xanthomatosis presenting with asymmetric parkinsonism: a case with I-123-FP-CIT SPECT imaging. *Acta Neurol Belg*. Sep 2012;112(3):287-9. doi:10.1007/s13760-012-0064-7

45. Su CS, Chang WN, Huang SH, et al. Cerebrotendinous xanthomatosis patients with and without parkinsonism: clinical characteristics and neuroimaging findings. *Mov Disord*. Mar 15 2010;25(4):452-8. doi:10.1002/mds.22979

46. Wakamatsu N, Hayashi M, Kawai H, et al. Mutations producing premature termination of translation and an amino acid substitution in the sterol 27-hydroxylase gene cause cerebrotendinous xanthomatosis associated with parkinsonism. *J Neurol Neurosurg Psychiatry*. Aug 1999;67(2):195-8. doi:10.1136/jnnp.67.2.195

47. Yunisova G, Tufekcioglu Z, Dogu O, et al. Patients with Lately Diagnosed Cerebrotendinous Xanthomatosis. *Neurodegener Dis*. Apr 29 2020:1-7. doi:10.1159/000506770

48. Zadori D, Szpisjak L, Madar L, et al. Different phenotypes in identical twins with cerebrotendinous xanthomatosis: case series. *Neurol Sci*. Mar 2017;38(3):481-483. doi:10.1007/s10072-016-2776-6

49. Rubio-Agusti I, Kojovic M, Edwards MJ, et al. Atypical parkinsonism and cerebrotendinous xanthomatosis: report of a family with corticobasal syndrome and a literature review. *Mov Disord*. Dec 2012;27(14):1769-74. doi:10.1002/mds.25229

50. Manti F, Nardecchia F, Barresi S, et al. Neurotransmitter trafficking defect in a patient with clathrin (CLTC) variation presenting with intellectual disability and early-onset parkinsonism. *Parkinsonism Relat Disord*. Apr 2019;61:207-210. doi:10.1016/j.parkreldis.2018.10.012

51. Fernandez HH, Friedman JH, Famiglietti EV. Probable Cornelia de Lange syndrome with progressive parkinsonism and dystonia. *Mov Disord*. Jul 2000;15(4):749-51. doi:10.1002/1531-8257(200007)15:4<749::aid-mds1028>3.0.co;2-p

52. Rana S. Multidisciplinary care of a patient with cowden syndrome at a geriatric center. *Journal of the American Geriatrics Society*. 2018;66(Supplement 2):S187. 2018 Annual Scientific Meeting of the American Geriatrics Society, AGS 2018. United States. doi:http://dx.doi.org/10.1111/jgs.15376

53. De Coo IF, Renier WO, Ruitenbeek W, et al. A 4-base pair deletion in the mitochondrial cytochrome b gene associated with parkinsonism/MELAS overlap syndrome. *Ann Neurol*. Jan 1999;45(1):130-3. doi:10.1002/1531-8249(199901)45:1<130::aid-art21>3.3.co;2-q

54. Sedel F, Ribeiro MJ, Remy P, Blau N, Saudubray JM, Agid Y. Dihydropteridine reductase deficiency: levodopa's long-term effectiveness without dyskinesia. *Neurology*. Dec 26 2006;67(12):2243-5. doi:10.1212/01.wnl.0000249335.35585.3e

55. Takahashi Y, Manabe Y, Nakano Y, et al. Parkinsonism in Association with Dihydropteridine Reductase Deficiency. *Case Rep Neurol*. Jan-Apr 2017;9(1):17-21. doi:10.1159/000456610

56. Porta F, Mussa A, Concolino D, Spada M, Ponzone A. Dopamine agonists in dihydropteridine reductase deficiency. *Mol Genet Metab*. Apr 2012;105(4):582-4. doi:10.1016/j.ymgme.2012.01.013

57. Straniero L, Guella I, Cilia R, et al. DNAJC12 and dopa-responsive nonprogressive parkinsonism. *Ann Neurol*. Oct 2017;82(4):640-646. doi:10.1002/ana.25048

58. Anikster Y, Haack TB, Vilboux T, et al. Biallelic Mutations in DNAJC12 Cause Hyperphenylalaninemia, Dystonia, and Intellectual Disability. *Am J Hum Genet*. Feb 2 2017;100(2):257-266. doi:10.1016/j.ajhg.2017.01.002

59. Koroglu C, Baysal L, Cetinkaya M, Karasoy H, Tolun A. DNAJC6 is responsible for juvenile parkinsonism with phenotypic variability. *Parkinsonism Relat Disord*. Mar 2013;19(3):320-4. doi:10.1016/j.parkreldis.2012.11.006

60. Ng J, Cortes-Saladelafont E, Abela L, et al. DNAJC6 Mutations Disrupt Dopamine Homeostasis in Juvenile Parkinsonism-Dystonia. *Mov Disord*. May 30 2020;doi:10.1002/mds.28063

61. Termsarasab P, Pearson TS. Juvenile Parkinsonism and epilepsy due to homozygous mutations in DNAJC6. *Movement Disorders*. 2015;30(SUPPL. 1):S459-S460. 19th International Congress of Parkinson's Disease and Movement Disorders. San Diego, CA United States.

(var.pagings). doi:http://dx.doi.org/10.1002/mds.26295

62. Edvardson S, Cinnamon Y, Ta-Shma A, et al. A deleterious mutation in DNAJC6 encoding the neuronal-specific clathrin-uncoating co-chaperone auxilin, is associated with juvenile parkinsonism. *PLoS One*. 2012;7(5):e36458. doi:10.1371/journal.pone.0036458

63. Elsayed LE, Drouet V, Usenko T, et al. A Novel Nonsense Mutation in DNAJC6 Expands the Phenotype of Autosomal-Recessive Juvenile-Onset Parkinson's Disease. *Ann Neurol*. Feb 2016;79(2):335-7. doi:10.1002/ana.24591

64. Tassin J, Durr A, Bonnet AM, et al. Levodopa-responsive dystonia. GTP cyclohydrolase I or parkin mutations? *Brain*. Jun 2000;123 ( Pt 6):1112-21. doi:10.1093/brain/123.6.1112

65. Kikuchi A, Takeda A, Fujihara K, et al. Arg(184)His mutant GTP cyclohydrolase I, causing recessive hyperphenylalaninemia, is responsible for dopa-responsive dystonia with parkinsonism: a case report. *Mov Disord*. May 2004;19(5):590-3. doi:10.1002/mds.10712

66. Wirth T, Mariani LL, Bergant G, et al. Loss-of-Function Mutations in NR4A2 Cause Dopa-Responsive Dystonia Parkinsonism. *Mov Disord*. May 2020;35(5):880-885. doi:10.1002/mds.27982

67. Zielonka M, Makhseed N, Blau N, Bettendorf M, Hoffmann GF, Opladen T. Dopamine-Responsive Growth-Hormone Deficiency and Central Hypothyroidism in Sepiapterin Reductase Deficiency. *JIMD Rep*. 2015;24:109-13. doi:10.1007/8904_2015_450

68. Bodhireddy S, Dickson DW, Mattiace L, Weidenheim KM. A case of Down's syndrome with diffuse Lewy body disease and Alzheimer's disease. *Neurology*. Jan 1994;44(1):159-61. doi:10.1212/wnl.44.1.159

69. Brandel JP, Marconi R, Serdaru M, Vidailhet M, Agid Y. Down's syndrome and Parkinson's disease. *Neurology*. Dec 1994;44(12):2419-20. doi:10.1212/wnl.44.12.2419-c

70. Agarwal P, Griffith AF, Borromeo-Wesner M. Case report and literature review of levodopa-reponsive young onset Parkinson's disease in a patient with Down's syndrome. *Movement Disorders*. 2010;25(SUPPL. 2):S499-S500. 14th International Congress of Parkinson's Disease and Movement Disorders. Buenos Aires Argentina.

(var.pagings). doi:http://dx.doi.org/10.1002/mds.23162

71. Marui W, Iseki E, Kosaka K, Kato M, Adachi Y, Ueda K. An autopsied case of Down syndrome with Alzheimer pathology and alpha-synuclein immunoreactivity. *Neuropathology*. 1999;19(4):410-416. doi:http://dx.doi.org/10.1046/j.1440-1789.1999.00265.x

72. Palat P, Hickey F, Patel L, Sannar E. Levodopa-Responsive Early-Onset Parkinsonism in Down Syndrome. *Case Rep Neurol Med*. 2018;2018:2314791. doi:10.1155/2018/2314791

73. Singer C, Sanchez-Ramos J, Weiner WJ. Levodopa-responsive parkinsonism in a patient with Down's syndrome. *Eur Neurol*. 1990;30(5):247-8. doi:10.1159/000117355

74. Storm W. Differential diagnosis and treatment of depressive features in Down's syndrome: a case illustration. *Res Dev Disabil*. 1990;11(2):131-7. doi:10.1016/0891-4222(90)90031-3

75. Sturman SG, Williams AC. Parkinsonism and Down syndrome. *Lancet*. Oct 14 1989;2(8668):920-1. doi:10.1016/s0140-6736(89)91581-x

76. Camargos S, Scholz S, Simon-Sanchez J, et al. DYT16, a novel young-onset dystonia-parkinsonism disorder: identification of a segregating mutation in the stress-response protein PRKRA. *Lancet Neurol*. Mar 2008;7(3):207-15. doi:10.1016/s1474-4422(08)70022-x

77. Keogh MJ, Daud D, Pyle A, et al. A novel de novo STXBP1 mutation is associated with mitochondrial complex I deficiency and late-onset juvenile-onset parkinsonism. *Neurogenetics*. 2014;16(1):65-67. doi:http://dx.doi.org/10.1007/s10048-014-0431-z

78. Rezazadeh A, Lira V, Silberberg A, et al. Stxbp1 is associated with bruxism in awake patients. *Neurology*. 2019;92(15 Supplement 1)71st Annual Meeting of the American Academy of Neurology, AAN 2019. United States.

79. Yildiz EP, Yesil G, Ozkan MU, Bektas G, Caliskan M, Ozmen M. A novel EPM2A mutation in a patient with Lafora disease presenting with early parkinsonism symptoms in childhood. *Seizure*. Oct 2017;51:77-79. doi:10.1016/j.seizure.2017.07.011

80. Hall D, Pickler L, Riley K, Tassone F, Hagerman R. Parkinsonism and cognitive decline in a fragile X mosaic male. *Mov Disord*. Jul 30 2010;25(10):1523-4. doi:10.1002/mds.23150

81. Rosario M, Moldovan O, Reimao S, et al. Juvenile parkinsonism associated with a novel HSD17B10 mutation in a patient with HSD10 disease. *Movement Disorders*. 2018;33(Supplement 2):S218. 22nd International Congress of Parkinson's Disease and Movement Disorders, MDS 2018. Hong Kong.

82. Chen JY, Oza VS, Gopi R, Christine CW. Rapidly progressive Parkinsonism in a patient with incontinentia pigmenti. *Movement Disorders*. 2015;30(SUPPL. 1):S476-S477. 19th International Congress of Parkinson's Disease and Movement Disorders. San Diego, CA United States.

(var.pagings). doi:http://dx.doi.org/10.1002/mds.26295

83. Bach JP, Sommer N, Moller JC, Oertel WH, Dodel R, Gasser T. Parkinson's syndrome in a young patient with Klinefelter's syndrome--a case report. *Mov Disord*. Apr 15 2008;23(5):771-2. doi:10.1002/mds.21948

84. Lee KS, Lee JE, Cho AH, Kim JS, Oh YS. A case of Parkinson's disease in a patient with Klinefelter's syndrome. *Acta Neurologica Belgica*. 2019;doi:http://dx.doi.org/10.1007/s13760-019-01232-1

85. Yu W, He Y, Liu Y. Combined Klinefelter syndrome and hereditary early-onset Parkinson's disease: A case report. *Movement Disorders Clinical Practice*. 2019;6(Supplement 1):S82. 6th Asian and Oceanian Parkinson's Disease and Movement Disorders Congress. China. doi:http://dx.doi.org/10.1002/mdc3.12744

86. Fabbri M, Zibetti M, Martone T, Lopiano L. Expanding the spectrum of movement disorders in Klinefelter syndrome. *Neurol Sci*. Jul 2018;39(7):1303-1304. doi:10.1007/s10072-018-3296-3

87. Owens WE, Okun MS. Dystonia, tremor, and parkinsonism in a 54 year old man with 2-hydroxyglutaric aciduria. *J Neurol Neurosurg Psychiatry*. Sep 2004;75(9):1362-3. doi:10.1136/jnnp.2003.033571

88. Martikainen MH, Ng YS, Gorman GS, et al. Clinical, genetic, and radiological features of extrapyramidal movement disorders in mitochondrial disease. *JAMA Neurology*. 2016;73(6):668-674. doi:http://dx.doi.org/10.1001/jamaneurol.2016.0355

89. Baumgartner A, Nia S, Erdler M, et al. A novel heteroplasmic mutation in the mitochondrial ATP6 gene associated with Parkinson's syndrome and epilepsy. *Journal of the Neurological Sciences*. 2013;333(SUPPL. 1):e706. 21st World Congress of Neurology. Vienna Austria.

(var.pagings). doi:http://dx.doi.org/10.1016/j.jns.2013.07.2437

90. Hemelsoet DM, Vanlander AV, Smet J, et al. Leigh syndrome followed by parkinsonism in an adult with homozygous c.626C>T mutation in MTFMT. *Neurology: Genetics*. 2018;4(6):e298. doi:http://dx.doi.org/10.1212/NXG.0000000000000298

91. Martikainen MH, Kytovuori L, Majamaa K. Juvenile parkinsonism, hypogonadism and Leigh-like MRI changes in a patient with m.4296G>A mutation in mitochondrial DNA. *Mitochondrion*. Mar 2013;13(2):83-6. doi:10.1016/j.mito.2013.01.012

92. Zyss J, Vlaicu M, Gerber S, Rodallec M, Gauthier C, Zuber M. Movement disorders in mevalonic aciduria. *Movement Disorders*. 2011;26(SUPPL. 2):S256. 15th International Congress of Parkinson's Disease and Movement Disorders. Toronto, ON Canada.

(var.pagings). doi:http://dx.doi.org/10.1002/mds.23764

93. Alkufri F, Harrower T, Rahman Y, et al. Molybdenum cofactor deficiency presenting with a parkinsonism-dystonia syndrome. *Mov Disord*. Mar 2013;28(3):399-401. doi:10.1002/mds.25276

94. Hara M, Inokuchi T, Taniwaki T, et al. An adult patient with mucolipidosis III alpha/beta presenting with parkinsonism. *Brain Dev*. May 2013;35(5):462-5. doi:10.1016/j.braindev.2012.07.009

95. Choi J, Lee J, Kim H, Jeon B. A patient with myotonic dystrophy type 1 and parkinsonism. *Movement Disorders*. 2018;33(Supplement 2):S803-S804. 22nd International Congress of Parkinson's Disease and Movement Disorders, MDS 2018. Hong Kong.

96. Pradotto L, Mencarelli M, Di Blasio AM, Mauro A. Co-occurrence of Parkinson's disease and neurofibromatosis: A casual association or an underlying unique genetic cause? *Clinical Neuropathology*. 2014;33(3):232. Joint Meeting 50th Congress of the Italian Association of Neuropathology and Clinical Neurobiology, AINPeNC - 40th Congress of the Italian Association for Research on Brain Aging, AIRIC. Verbania Italy.

(var.pagings).

97. Wattanapanom P, May C, Jain R. Weight loss as an atypical presentation of pheochromocytoma in an older man. *Journal of the American Geriatrics Society*. 2011;59(SUPPL. 1):S117. 2011 Annual Scientific Meeting of the American Geriatrics Society. National Harbor, MD United States.

(var.pagings). doi:http://dx.doi.org/10.1111/j.1532-5415.2011.03416.x

98. D'Ambrosio G, Vizioli R. Recklinghausen disease and Parkinson disease: an unusual association. *Acta Neurol (Napoli)*. Aug 1984;6(4):274-9.

99. Hattori S, Kiguchi H, Ishii T, Nakajima T, Yatsuzuka H. Moyamoya disease with concurrent von Recklinghausen's disease and cerebral arteriovenous malformation. *Pathol Res Pract*. 1998;194(5):363-9. doi:10.1016/s0344-0338(98)80061-7

100. Chandra S, Rao K, Ghosh A, Ray J, Northrup H, Stimming E. Parkinsonism in Christianson syndrome: A unique presentation of a unique syndrome. *Movement Disorders*. 2018;33(Supplement 2):S219. 22nd International Congress of Parkinson's Disease and Movement Disorders, MDS 2018. Hong Kong.

101. Pescosolido MF, Kavanaugh BC, Pochet N, et al. Complex Neurological Phenotype in Female Carriers of NHE6 Mutations. *Mol Neuropsychiatry*. Apr 2019;5(2):98-108. doi:10.1159/000496341

102. Araki K, Nakamura R, Ito D, et al. NUS1 mutation in a family with epilepsy, cerebellar ataxia, and tremor. *Epilepsy Res*. May 22 2020;164:106371. doi:10.1016/j.eplepsyres.2020.106371

103. Namihira T, Hattori N, Shiroma S, Miyazato Y. Autosomal recessive juvenile Parkinson's disease with partial trisomy of chromosome 6q syndrome: a case report. *Psychiatry Clin Neurosci*. Dec 2004;58(6):672-3. doi:10.1111/j.1440-1819.2004.01321.x

104. Garraux G, Caberg JH, Vanbellinghen JF, et al. Partial trisomy 4q associated with young-onset dopa-responsive parkinsonism. *Arch Neurol*. Mar 2012;69(3):398-400. doi:10.1001/archneurol.2011.802

105. Daelman L, Sedel F, Tourbah A. Progressive neuropsychiatric manifestations of phenylketonuria in adulthood. *Rev Neurol (Paris)*. Apr 2014;170(4):280-7. doi:10.1016/j.neurol.2013.09.012

106. Evans AH, Costa DC, Gacinovic S, et al. L-Dopa-responsive Parkinson's syndrome in association with phenylketonuria: In vivo dopamine transporter and D2 receptor findings. *Mov Disord*. Oct 2004;19(10):1232-6. doi:10.1002/mds.20146

107. Velema M, Boot E, Engelen M, Hollak C. Parkinsonism in phenylketonuria: a consequence of dopamine depletion? *JIMD Rep*. 2015;20:35-8. doi:10.1007/8904_2014_386

108. Leuzzi V, Trasimeni G, Gualdi GF, Antonozzi I. Biochemical, clinical and neuroradiological (MRI) correlations in late-detected PKU patients. *J Inherit Metab Dis*. 1995;18(5):624-34. doi:10.1007/bf02436009

109. Konrad PN, McCarthy DJ, Mauer AM, Valentine WN, Paglia DE. Erythrocyte and leukocyte phosphoglycerate kinase deficiency with neurologic disease. *J Pediatr*. Mar 1973;82(3):456-60. doi:10.1016/s0022-3476(73)80120-9

110. Sakaue S, Kasai T, Mizuta I, et al. Early-onset parkinsonism in a pedigree with phosphoglycerate kinase deficiency and a heterozygous carrier: do PGK-1 mutations contribute to vulnerability to parkinsonism? *NPJ Parkinsons Dis*. 2017;3:13. doi:10.1038/s41531-017-0014-4

111. Virmani T, Rotstein M, Spiegel R, Akman HO, DiMauro S, Greene PE. Levodopa Responsive Parkinsonism in Two Patients With Phosphoglycerate Kinase Deficiency. *Mov Disord Clin Pract*. Sep 2014;1(3):240-242. doi:10.1002/mdc3.12055

112. Morales-Briceno H, Ha AD, London K, Farlow D, Chang FCF, Fung VSC. Parkinsonism in PGK1 deficiency implicates the glycolytic pathway in nigrostriatal dysfunction. *Parkinsonism Relat Disord*. Jul 2019;64:319-323. doi:10.1016/j.parkreldis.2019.04.004

113. Rotstein M, Spiegel R, DiMauro S. Juvenile parkinsonism and myopathy caused by a T378P mutation of the phosphoglycerate kinase gene PGK1. *Movement Disorders*. 2012;27(SUPPL. 1):S69. 16th International Congress of Parkinson's Disease and Movement Disorders. Dublin Ireland.

(var.pagings). doi:http://dx.doi.org/10.1002/mds.25051

114. Sotiriou E, Greene P, Krishna S, Hirano M, DiMauro S. Myopathy and parkinsonism in phosphoglycerate kinase deficiency. *Muscle Nerve*. May 2010;41(5):707-10. doi:10.1002/mus.21612

115. Echaniz-Laguna A, Nadjar Y, Behin A, et al. Phosphoglycerate kinase deficiency: A nationwide multicenter retrospective study. *J Inherit Metab Dis*. Sep 2019;42(5):803-808. doi:10.1002/jimd.12087

116. Bandettini di Poggio M, Nesti C, Bruno C, Meschini MC, Schenone A, Santorelli FM. Dopamine-agonist responsive Parkinsonism in a patient with the SANDO syndrome caused by POLG mutation. *BMC Med Genet*. Oct 7 2013;14:105. doi:10.1186/1471-2350-14-105

117. De Pue A, Santens P. Late onset ophthalmoplegia and parkinsonism due to a novel POLG mutation. *European Journal of Neurology*. 2016;23(SUPPL. 2):297. 2nd Congress of the European Academy of Neurology. Copenhagen Denmark.

(var.pagings). doi:http://dx.doi.org/10.1111/ene.13092

118. Khodadadi H, Azcona LJ, Aghamollaii V, et al. PTRHD1 (C2orf79) mutations lead to autosomal-recessive intellectual disability and parkinsonism. *Mov Disord*. Feb 2017;32(2):287-291. doi:10.1002/mds.26824

119. Jaberi E, Rohani M, Shahidi GA, et al. Mutation in ADORA1 identified as likely cause of early-onset parkinsonism and cognitive dysfunction. *Mov Disord*. Jul 2016;31(7):1004-11. doi:10.1002/mds.26627

120. Kuipers DJS, Carr J, Bardien S, et al. PTRHD1 Loss-of-function mutation in an african family with juvenile-onset Parkinsonism and intellectual disability. *Mov Disord*. Nov 2018;33(11):1814-1819. doi:10.1002/mds.27501

121. Ortez C, Jou C, Cortes-Saladelafont E, et al. Infantile parkinsonism and GABAergic hypotransmission in a patient with pyruvate carboxylase deficiency. *Gene*. Dec 15 2013;532(2):302-6. doi:10.1016/j.gene.2013.08.036

122. Ciammola A, Carrera P, Di Fonzo A, et al. X-linked Parkinsonism with Intellectual Disability caused by novel mutations and somatic mosaicism in RAB39B gene. *Parkinsonism and Related Disorders*. 2017;44:142-146. doi:http://dx.doi.org/10.1016/j.parkreldis.2017.08.021

123. Guldner M, Schulte C, Hauser AK, Gasser T, Brockmann K. Broad clinical phenotype in Parkinsonism associated with a base pair deletion in RAB39B and additional POLG variant. *Parkinsonism Relat Disord*. Oct 2016;31:148-150. doi:10.1016/j.parkreldis.2016.07.005

124. Lesage S, Bras J, Cormier-Dequaire F, et al. Loss-of-function mutations in RAB39B are associated with typical early-onset Parkinson disease. *Neurol Genet*. Jun 2015;1(1):e9. doi:10.1212/nxg.0000000000000009

125. Wilson GR, Sim JC, McLean C, et al. Mutations in RAB39B cause X-linked intellectual disability and early-onset Parkinson disease with alpha-synuclein pathology. *Am J Hum Genet*. Dec 4 2014;95(6):729-35. doi:10.1016/j.ajhg.2014.10.015

126. Roze E, Cochen V, Sangla S, et al. Rett syndrome: an overlooked diagnosis in women with stereotypic hand movements, psychomotor retardation, Parkinsonism, and dystonia? *Mov Disord*. Feb 15 2007;22(3):387-9. doi:10.1002/mds.21276

127. Chahil G, Yelam A, Bollu PC. Rett Syndrome in Males: A Case Report and Review of Literature. *Cureus*. Oct 4 2018;10(10):e3414. doi:10.7759/cureus.3414

128. Venkateswaran S, McMillan HJ, Doja A, Humphreys P. Adolescent onset cognitive regression and neuropsychiatric symptoms associated with the A140V MECP2 mutation. *Dev Med Child Neurol*. Jan 2014;56(1):91-4. doi:10.1111/dmcn.12334

129. Pollini L, Galosi S, Nardecchia F, et al. Parkinsonism, Intellectual Disability, and Catatonia in a Young Male With MECP2 Variant. *Mov Disord Clin Pract*. Jan 2020;7(1):118-119. doi:10.1002/mdc3.12865

130. Ollivier Y, Magot A, Latour P, et al. Clinical and electrophysiological features in a French family presenting with seipinopathy. *Neuromuscul Disord*. Feb 2015;25(2):161-4. doi:10.1016/j.nmd.2014.10.006

131. Goizet C, Boukhris A, Mundwiller E, et al. Complicated forms of autosomal dominant hereditary spastic paraplegia are frequent in SPG10. *Human mutation*. 2009;30(2):E376-E385. doi:http://dx.doi.org/10.1002/humu.20920

132. Anheim M, Lagier-Tourenne C, Stevanin G, et al. SPG11 spastic paraplegia. A new cause of juvenile parkinsonism. *J Neurol*. Jan 2009;256(1):104-8. doi:10.1007/s00415-009-0083-3

133. Guidubaldi A, Piano C, Santorelli FM, et al. Novel mutations in SPG11 cause hereditary spastic paraplegia associated with early-onset levodopa-responsive Parkinsonism. *Movement Disorders*. 2011;26(3):553-556. doi:http://dx.doi.org/10.1002/mds.23552

134. Kang SY, Lee MH, Lee SK, Sohn YH. Levodopa-responsive parkinsonism in hereditary spastic paraplegia with thin corpus callosum. *Parkinsonism and Related Disorders*. 2004;10(7):425-427. doi:http://dx.doi.org/10.1016/j.parkreldis.2004.05.003

135. Damasio J, Correia AP, Silva J, Alonso I, Bastos Lima A, Magalhaes M. Childhood-onset levodopa-responsive Parkinsonism in hereditary spastic paraplegia 15. *Movement Disorders*. 2014;29(SUPPL. 1):S397. 18th International Congress of Parkinson's Disease and Movement Disorders. Stockholm Sweden.

(var.pagings). doi:http://dx.doi.org/10.1002/mds.25914

136. Mallaret M, Lagha-Boukbiza O, Biskup S, et al. SPG15: A cause of juvenile atypical levodopa responsive Parkinsonism. *Movement Disorders*. 2014;29(SUPPL. 1):S55-S56. 18th International Congress of Parkinson's Disease and Movement Disorders. Stockholm Sweden.

(var.pagings). doi:http://dx.doi.org/10.1002/mds.25914

137. Schicks J, Synofzik M, Petursson H, et al. Atypical juvenile parkinsonism in a consanguineous SPG15 family. *Mov Disord*. Feb 15 2011;26(3):564-6. doi:10.1002/mds.23472

138. Groth CL, Berman BD. Spinocerebellar ataxia 27: A review and characterization of an evolving phenotype. *Tremor and Other Hyperkinetic Movements*. 2018;8doi:http://dx.doi.org/10.7916/D80S0ZJQ

139. Ebrahimi-Fakhari D, Hildebrandt C, Davis PE, Rodan LH, Anselm I, Bodamer O. The Spectrum of Movement Disorders in Childhood-Onset Lysosomal Storage Diseases. *Mov Disord Clin Pract*. Mar-Apr 2018;5(2):149-155. doi:10.1002/mdc3.12573

140. Nitschke S, Hagele-Link S, Bauer R, Mock B, Tettenborn B. Deep brain stimulation of the STN in a patient suffering from Parkinson's disease and Turner syndrome. *Schweizer Archiv fur Neurologie und Psychiatrie*. 2011;162(SUPPL. 4):9S. Gemeinsame Jahrestagung Schweizerische Neurologische Gesellschaft Schweizerische Gesellschaft fur Schlafforschung, Schlafmedizin und Chronobiologie 2011. St. Gallen Switzerland.

(var.pagings).

141. Westenberger A, Rosales RL, Heinitz S, et al. X-linked Dystonia-Parkinsonism manifesting in a female patient due to atypical turner syndrome. *Mov Disord*. May 2013;28(5):675-8. doi:10.1002/mds.25369

142. de Rijk-Van Andel JF, Gabreels FJ, Geurtz B, et al. L-dopa-responsive infantile hypokinetic rigid parkinsonism due to tyrosine hydroxylase deficiency. *Neurology*. Dec 26 2000;55(12):1926-8. doi:10.1212/wnl.55.12.1926

143. Pons R, Serrano M, Ormazabal A, et al. Tyrosine hydroxylase deficiency in three Greek patients with a common ancestral mutation. *Movement Disorders*. 2010;25(8):1086-1090. doi:http://dx.doi.org/10.1002/mds.23002

144. Grattan-Smith PJ, Wevers RA, Steenbergen-Spanjers GC, Fung VS, Earl J, Wilcken B. Tyrosine hydroxylase deficiency: clinical manifestations of catecholamine insufficiency in infancy. *Mov Disord*. Mar 2002;17(2):354-9. doi:10.1002/mds.10095

145. Haugarvoll K, Bindoff LA. A novel compound heterozygous tyrosine hydroxylase mutation (p.R441P) with complex phenotype. *J Parkinsons Dis*. 2011;1(1):119-22. doi:10.3233/jpd-2011-11006

146. Lüdecke B, Knappskog PM, Clayton PT, et al. Recessively inherited L-DOPA-responsive parkinsonism in infancy caused by a point mutation (L205P) in the tyrosine hydroxylase gene. *Hum Mol Genet*. Jul 1996;5(7):1023-8. doi:10.1093/hmg/5.7.1023

147. Swaans RJ, Rondot P, Renier WO, Van Den Heuvel LP, Steenbergen-Spanjers GC, Wevers RA. Four novel mutations in the tyrosine hydroxylase gene in patients with infantile parkinsonism. *Ann Hum Genet*. Jan 2000;64(Pt 1):25-31. doi:10.1017/s0003480000007922

148. Burke EA, Frucht SJ, Thompson K, et al. Biallelic mutations in mitochondrial tryptophanyl-tRNA synthetase cause Levodopa-responsive infantile-onset Parkinsonism. *Clin Genet*. Mar 2018;93(3):712-718. doi:10.1111/cge.13172

149. Galosi S, Martinelli S, Cordeddu V, et al. Digenic inheritance of WARS2 and CHRNA6 mutations in infantile Parkinsonism. *Movement Disorder*. 2019;34(Supplement 2):S209. 2019 International Parkinson and Movement Disorder Society, MDS 2019. France.

150. Horn MA, Mikaelsen KB, Ferdinandusse S, et al. Mild phenotype in an adult male with X-linked adrenoleukodystrophy - case report. *Clin Case Rep*. Feb 2016;4(2):177-81. doi:10.1002/ccr3.434

151. Serra Soler G, Gogorza Perez MS, Jimenez Portilla A, Pereg Macazaga V. Primary adrenal insufficiency due to X-linked adrenoleukodystrophy diagnosed in adulthood. *Endocrinol Diabetes Nutr*. Oct 2017;64(8):458-459. Insuficiencia suprarrenal debida a adrenoleucodistrofia ligada al cromosoma X diagnosticada en la edad adulta. doi:10.1016/j.endinu.2017.07.001

152. Gupta HV, Vengoechea J, Sahaya K, Virmani T. A splice site mutation in ATP6AP2 causes X-linked intellectual disability, epilepsy, and parkinsonism. *Parkinsonism Relat Disord*. Dec 2015;21(12):1473-5. doi:10.1016/j.parkreldis.2015.10.001

153. Meytin L. S-PR, Swan M. . Myoclonus and parkinsonism in 22q11.2 syndrome-expanding the phenotype. *Mov Disord Clin Pract*. 2020;(7(Supplement 1)):S83. doi:doi:10.1002/mdc3.12905

154. Buongarzone G, Minafra B, Errichiello E, et al. 13.3.1. Movement disorders in a family carrying ATP7A variant. *Movement Disorders*. 2020;35(SUPPL 1):S89. MDS International Congress. Virtual. doi:http://dx.doi.org/10.1002/mds.28268

155. Maric N, Luna J, Codina M, Armengol L. Severe scoliosis in the patient with a novel variant p. Pro237ArgfsTer7 in the WDR45 gene. *Eur J Hum Genet*. 2020;(28(SUPPL 1)):453-454. doi: doi:10.1038/s41431-020-00739-z

156. Umehara F, Iwama K, Mizuguchi T, Matsumoto N. Basal ganglia calcification in a patient with static encephalopathy of childhood with neurodegeneration in adulthood/β-propeller protein-associated neurodegeneration. *Neurology and Clinical Neuroscience*. 2020;8(5):332-334. doi:https://doi.org/10.1111/ncn3.12427

157. Samanta D, Ramakrishnaiah R. Early-Onset Parkinsonism and Halo Sign: Beta-propeller Proteinassociated Neurodegeneration. *J Pediatr Neurosci*. Jul-Sep 2020;15(3):325-327. doi:10.4103/jpn.JPN_62_20

158. Munoz-Delgado L, Jesus S, Macias-Garcia D, et al. Atypical parkinsonism due to a mutation in the SLC9A6 gene. . *Mov Disord*. 2020;(35(SUPPL 1)):S516-S517. doi:doi:10.1002/mds.28268

159. Zadori D, Szalardy L, Maszlag-Torok R, et al. Clinicopathological Relationships in an Aged Case of DOORS Syndrome With a p.Arg506X Mutation in the ATP6V1B2 Gene. *Frontiers in Neurology*. 2020;11:767. doi:http://dx.doi.org/10.3389/fneur.2020.00767

160. Jesús S, Hinarejos I, Carrillo F, et al. NR4A2 Mutations Can Cause Intellectual Disability and Language Impairment With Persistent Dystonia-Parkinsonism. *Neurol Genet*. Feb 2021;7(1):e543. doi:10.1212/nxg.0000000000000543

161. Sleiman PM, Healy DG, Muqit MM, et al. Characterisation of a novel NR4A2 mutation in Parkinson's disease brain. *Neurosci Lett*. Jun 26 2009;457(2):75-9. doi:10.1016/j.neulet.2009.03.021

162. Grimes DA, Han F, Panisset M, et al. Translated mutation in the Nurr1 gene as a cause for Parkinson's disease. *Mov Disord*. Jul 2006;21(7):906-9. doi:10.1002/mds.20820

163. Kanatani M, Adachi T, Sakata R, et al. Dravet syndrome with parkinsonian symptoms and intact dopaminergic neurons: A case report. *Brain Dev*. Mar 2021;43(3):486-489. doi:10.1016/j.braindev.2020.10.015

164. Malaquias MJ, Costa D, Pinto E, et al. Parkinsonism and iron deposition in two adult patients with L-2-hydroxiglutaric aciduria. *Parkinsonism Relat Disord*. May 2021;86:45-47. doi:10.1016/j.parkreldis.2021.03.025

165. Witt J, Davis M. PNPLA6-related disorder with expanded phenotype including parkinsonism, dystonia, and abnormal dopamine transporter imaging. *Mov Disord*. 2020;(35(SUPPL 1)):S575. doi:doi:10.1002/mds.28268

166. Kim CY, Wirth T, Hubsch C, et al. Early-Onset Parkinsonism Is a Manifestation of the PPP2R5D p.E200K Mutation. *Ann Neurol*. Nov 2020;88(5):1028-1033. doi:10.1002/ana.25863

167. Hetzelt K, Kerling F, Kraus C, et al. Early-onset parkinsonism in PPP2R5D-related neurodevelopmental disorder. *Eur J Med Genet*. Jan 2021;64(1):104123. doi:10.1016/j.ejmg.2020.104123

168. Nomura S, Kashiwagi M, Tanabe T, et al. Rapid-onset dystonia-parkinsonism with ATP1A3 mutation and left lower limb paroxysmal dystonia. *Brain Dev*. Apr 2021;43(4):566-570. doi:10.1016/j.braindev.2020.12.009

169. Araujo FMM, Junior WM, Tomaselli PJ, Pimentel Â V, Macruz Brito MC, Tumas V. SPG15: A Rare Correlation with Atypical Juvenile Parkinsonism Responsive to Levodopa. *Mov Disord Clin Pract*. Oct 2020;7(7):842-844. doi:10.1002/mdc3.13027

170. Lesage S, Mangone G, Tesson C, et al. Clinical Variability of SYNJ1-Associated Early-Onset Parkinsonism. Brief Research Report. *Frontiers in Neurology*. 2021-March-25 2021;12(366)doi:10.3389/fneur.2021.648457

171. Krebs CE, Karkheiran S, Powell JC, et al. The Sac1 domain of SYNJ1 identified mutated in a family with early-onset progressive Parkinsonism with generalized seizures. *Hum Mutat*. Sep 2013;34(9):1200-7. doi:10.1002/humu.22372

172. Quadri M, Fang M, Picillo M, et al. Mutation in the SYNJ1 gene associated with autosomal recessive, early-onset Parkinsonism. *Hum Mutat*. Sep 2013;34(9):1208-15. doi:10.1002/humu.22373

173. Olgiati S, De Rosa A, Quadri M, et al. PARK20 caused by SYNJ1 homozygous Arg258Gln mutation in a new Italian family. *Neurogenetics*. Aug 2014;15(3):183-8. doi:10.1007/s10048-014-0406-0

174. Kirola L, Behari M, Shishir C, Thelma BK. Identification of a novel homozygous mutation Arg459Pro in SYNJ1 gene of an Indian family with autosomal recessive juvenile Parkinsonism. *Parkinsonism Relat Disord*. Oct 2016;31:124-128. doi:10.1016/j.parkreldis.2016.07.014

175. Taghavi S, Chaouni R, Tafakhori A, et al. A Clinical and Molecular Genetic Study of 50 Families with Autosomal Recessive Parkinsonism Revealed Known and Novel Gene Mutations. *Mol Neurobiol*. Apr 2018;55(4):3477-3489. doi:10.1007/s12035-017-0535-1

176. Ben Romdhan S, Sakka S, Farhat N, Triki S, Dammak M, Mhiri C. A Novel SYNJ1 Mutation in a Tunisian Family with Juvenile Parkinson's Disease Associated with Epilepsy. *J Mol Neurosci*. Oct 2018;66(2):273-278. doi:10.1007/s12031-018-1167-2

177. Hong D, Cong L, Zhong S, et al. Clonazepam improves the symptoms of two siblings with novel variants in the SYNJ1 gene. *Parkinsonism Relat Disord*. May 2019;62:221-225. doi:10.1016/j.parkreldis.2018.11.020

178. Xie F, Chen S, Cen ZD, et al. A novel homozygous SYNJ1 mutation in two siblings with typical Parkinson's disease. *Parkinsonism Relat Disord*. Dec 2019;69:134-137. doi:10.1016/j.parkreldis.2019.11.001

179. Boot E, Butcher NJ, Udow S, et al. Typical features of Parkinson disease and diagnostic challenges with microdeletion 22q11.2. *Neurology*. Jun 5 2018;90(23):e2059-e2067. doi:10.1212/wnl.0000000000005660

180. Boot E, Mentzel TQ, Palmer LD, et al. Age-Related Parkinsonian Signs in Microdeletion 22q11.2. *Mov Disord*. May 9 2020;doi:10.1002/mds.28080

181. Butcher NJ, Boot E, Lang AE, et al. Neuropsychiatric expression and catatonia in 22q11.2 deletion syndrome: An overview and case series. *Am J Med Genet A*. Oct 2018;176(10):2146-2159. doi:10.1002/ajmg.a.38708

182. Butcher NJ, Kiehl TR, Hazrati LN, et al. Association between early-onset Parkinson disease and 22q11.2 deletion syndrome: identification of a novel genetic form of Parkinson disease and its clinical implications. *JAMA Neurol*. Nov 2013;70(11):1359-66. doi:10.1001/jamaneurol.2013.3646

183. Butcher NJ, Marras C, Pondal M, et al. Neuroimaging and clinical features in adults with a 22q11.2 deletion at risk of Parkinson's disease. *Brain*. May 1 2017;140(5):1371-1383. doi:10.1093/brain/awx053

184. Dufournet B, Nguyen K, Charles P, et al. Parkinson's disease associated with 22q11.2 deletion: Clinical characteristics and response to treatment. *Rev Neurol (Paris)*. Jun 2017;173(6):406-410. doi:10.1016/j.neurol.2017.03.021

185. Mok KY, Sheerin U, Simon-Sanchez J, et al. Deletions at 22q11.2 in idiopathic Parkinson's disease: a combined analysis of genome-wide association data. *Lancet Neurol*. May 2016;15(6):585-96. doi:10.1016/s1474-4422(16)00071-5

186. Nishioka K, Oyama G, Yoshino H, et al. High frequency of beta-propeller protein-associated neurodegeneration (BPAN) among patients with intellectual disability and young-onset parkinsonism. *Neurobiol Aging*. May 2015;36(5):2004.e9-2004.e15. doi:10.1016/j.neurobiolaging.2015.01.020

187. Morales-Briceno H, Sanchez-Hernandez BE, Meyer E, et al. Beta-propeller-associated neurodegeneration can present with dominant or isolated parkinsonism. *Mov Disord*. Apr 2018;33(4):654-656. doi:10.1002/mds.27294

188. Hayflick SJ, Kruer MC, Gregory A, et al. beta-Propeller protein-associated neurodegeneration: a new X-linked dominant disorder with brain iron accumulation. *Brain*. Jun 2013;136(Pt 6):1708-17. doi:10.1093/brain/awt095

189. Ohno T, Kobayashi S, Hayashi M, Sakurai M, Kanazawa I. Diphenylpyraline-responsive parkinsonism in cerebrotendinous xanthomatosis: long-term follow up of three patients. *J Neurol Sci*. Jan 1 2001;182(2):95-7. doi:10.1016/s0022-510x(00)00441-x

190. Olgiati S, Quadri M, Fang M, et al. DNAJC6 Mutations Associated With Early-Onset Parkinson's Disease. *Ann Neurol*. Feb 2016;79(2):244-56. doi:10.1002/ana.24553

191. Clot F, Grabli D, Cazeneuve C, et al. Exhaustive analysis of BH4 and dopamine biosynthesis genes in patients with Dopa-responsive dystonia. *Brain*. Jul 2009;132(Pt 7):1753-63. doi:10.1093/brain/awp084

192. Ng J, Meyer E, Li Y, et al. Expansion of the genotypic and phenotypic spectrum of hereditary dopamine transporter deficiency syndrome. *Journal of Inherited Metabolic Disease*. 2012;35(1 SUPPL. 1):S19. Annual Symposium of the Society for the Study of Inborn Errors of Metabolism, SSIEM 2012. Birmingham United Kingdom.

(var.pagings). doi:http://dx.doi.org/10.1007/s10545-012-9512-z

193. Lai F, Williams RS. A prospective study of Alzheimer disease in Down syndrome. *Arch Neurol*. Aug 1989;46(8):849-53. doi:10.1001/archneur.1989.00520440031017

194. Vieregge P, Ziemens G, Freudenberg M, Piosinski A, Muysers A, Schulze B. Extrapyramidal features in advanced Down's syndrome: clinical evaluation and family history. *J Neurol Neurosurg Psychiatry*. Jan 1991;54(1):34-8. doi:10.1136/jnnp.54.1.34

195. Fasano A, Borlot F, Lang AE, Andrade DM. Antecollis and levodopa-responsive parkinsonism are late features of dravet syndrome. *Neurology*. 2014;82(24):2250-2251. doi:http://dx.doi.org/10.1212/WNL.0000000000000521

196. Utari A, Adams E, Berry-Kravis E, et al. Aging in fragile X syndrome. *J Neurodev Disord*. Jun 2010;2(2):70-76. doi:10.1007/s11689-010-9047-2

197. Gitiaux C, Roze E, Kinugawa K, et al. Spectrum of movement disorders associated with glutaric aciduria type 1: a study of 16 patients. *Mov Disord*. Dec 15 2008;23(16):2392-7. doi:10.1002/mds.22313

198. Ruottinen HM, Rinne JO, Haaparanta M, et al. [18F]fluorodopa PET shows striatal dopaminergic dysfunction in juvenile neuronal ceroid lipofuscinosis. *J Neurol Neurosurg Psychiatry*. Jun 1997;62(6):622-5. doi:10.1136/jnnp.62.6.622

199. Aberg L, Liewendahl K, Nikkinen P, Autti T, Rinne JO, Santavuori P. Decreased striatal dopamine transporter density in JNCL patients with parkinsonian symptoms. *Neurology*. Mar 14 2000;54(5):1069-74. doi:10.1212/wnl.54.5.1069

200. Aberg LE, Rinne JO, Rajantie I, Santavuori P. A favorable response to antiparkinsonian treatment in juvenile neuronal ceroid lipofuscinosis. *Neurology*. May 8 2001;56(9):1236-9. doi:10.1212/wnl.56.9.1236

201. Hunter H. A controlled study of the psychopathology and physical measurements of Klinefelter's syndrome. *Br J Psychiatry*. Apr 1969;115(521):443-8. doi:10.1192/bjp.115.521.443

202. Madubata CC, Olsen MA, Stwalley DL, Gutmann DH, Johnson KJ. Neurofibromatosis type 1 and chronic neurological conditions in the United States: an administrative claims analysis. *Genet Med*. Jan 2015;17(1):36-42. doi:10.1038/gim.2014.70

203. Luoma P, Melberg A, Rinne JO, et al. Parkinsonism, premature menopause, and mitochondrial DNA polymerase gamma mutations: clinical and molecular genetic study. *Lancet*. Sep 4-10 2004;364(9437):875-82. doi:10.1016/s0140-6736(04)16983-3

204. Orrico A, Lam C, Galli L, et al. MECP2 mutation in male patients with non-specific X-linked mental retardation. *FEBS Lett*. Sep 22 2000;481(3):285-8. doi:10.1016/s0014-5793(00)01994-3

205. FitzGerald PM, Jankovic J, Percy AK. Rett syndrome and associated movement disorders. *Mov Disord*. 1990;5(3):195-202. doi:10.1002/mds.870050303

206. Kara E, Tucci A, Manzoni C, et al. Genetic and phenotypic characterization of complex hereditary spastic paraplegia. *Brain*. Jul 2016;139(Pt 7):1904-18. doi:10.1093/brain/aww111

207. Zouari M, Nehdi H, Ben Hamed M, Hentati F, Amouri R. Hereditary spastic paraplegia with thin corpus callosum: Clinical, radiological and genetic study. *Journal of the Neurological Sciences*. 2009;285(SUPPL. 1):S106. 19th World Congress of Neurology. Bangkok Thailand.

(var.pagings).

208. Korvatska O, Strand NS, Berndt JD, et al. Altered splicing of ATP6AP2 causes X-linked parkinsonism with spasticity (XPDS). *Hum Mol Genet*. Aug 15 2013;22(16):3259-68. doi:10.1093/hmg/ddt180
